# Discovery of *Poaceae*-based virion-assembly inhibitor for managing potato virus Y

**DOI:** 10.1186/s12870-025-07404-x

**Published:** 2025-10-06

**Authors:** Yuefei Long, Chunni Zhao, Huan Wu, Zhongjie Shen, Baoan Song, Deyu Hu

**Affiliations:** State Key Laboratory of Green Pesticide, Center for R&D of Fine Chemicals of Guizhou University, Guiyang, 550025 P. R. China

**Keywords:** Agrochemical, Benzoxazinoid, Coat protein, Potato virus Y, Viral assembly

## Abstract

**Background:**

The chemical management of potato virus Y (PVY)-induced viral diseases in *Solanaceae* crops remains a persistent challenge. Successful systemic infection and transmission of PVY depend on the formation of intact virions, suggesting that targeted inhibition of coat protein (CP)-mediated encapsidation of viral RNA could disrupt viral assembly. However, reported inhibitors capable of disrupting such a process remain scarce. The present study reports a series of analogues derived from benzoxazinoids—allelopathic secondary metabolites of *Poaceae* plants—and systematically evaluates their anti-PVY activity. Furthermore, the preliminary mode of action of the most potent compound to impair viral assembly is elucidated.

**Result:**

Thirty-four benzoxazinoids (L1-L34) containing sulfonamide moieties were synthesized via a three-step reaction protocol and evaluated for their virucidal activity using the half-leaf local-lesion assay. Most compounds demonstrated promising inactivation potency, with derivative L5 exhibiting a lower EC_50_ value (169.4 µg/mL) than the commercial control ribavirin (244.7 µg/mL). Molecular docking, dynamics simulations, and bio-layer interferometry revealed that VAL211 (V211) on the PVY CP likely serves as the critical binding residue for L5. Notably, in *Agrobacterium*-mediated infection assays, the V211A mutant virus exhibited markedly attenuated fluorescence intensity, while confocal microscopy confirmed unimpaired viral cell-to-cell movement. Conversely, transmission electron microscopy revealed a significant reduction in both viral particle quantity and length, suggesting that L5 inhibited CP-mediated viral RNA assembly.

**Conclusion:**

The study successfully developed L5, a benzoxazinoid-derived compound originating from secondary metabolites of *Poaceae* plants, which exhibited exceptional anti-PVY activity through serving as an inhibitor of virion assembly. These findings position L5 as a promising lead compound for the rational development of novel virion assembly inhibitors targeting CP-dependent processes in PVY and related plant viruses.

**Supplementary Information:**

The online version contains supplementary material available at 10.1186/s12870-025-07404-x.

## Background

Potato (*Solanum tuberosum*) and other *Solanaceae* crops, including tomato (*Solanum lycopersicum*), pepper (*Capsicum annuum*), and eggplant (*Solanum melongena*), are cornerstone species for global food security, collectively feeding over a billion people and contributing significantly to dietary diversity and economic stability [1, 2]. As the world’s third most consumed food crop, potato alone provides essential carbohydrates, vitamins, and minerals to humans [3]. However, the productivity of these crops is severely threatened by potato virus Y (PVY), a member of the *Potyvirus* genus and one of the most economically damaging plant pathogens worldwide [4]. PVY infection causes systemic necrosis, leaf mottling, and tuber deformation, reducing potato yields by up to 80% depending on viral strains and environmental conditions [5]. The virus is transmitted non-persistently by aphids, enabling rapid field-to-field spread, while its genetic plasticity facilitates the emergence of recombinant strains (e.g., PVY^NTN^ and PVY^N−Wi^) that evade host resistance [6, 7]. Compounding these challenges, current management strategies, such as chemical-based vector control and transgenic resistance, face limitations due to aphid resistance to insecticides, environmental concerns, and viral adaptability [8, 9].The urgency to develop alternative PVY control measures is underscored in this context.

Elucidating the infection mechanisms of PVY is critical for designing effective control method [10]. The PVY life cycle typically involves the following stages: virions are transmitted into plant cells via aphid vectors or mechanical inoculation, followed by decapsidation to release the viral genome and initiate translation. The resulting polyprotein is proteolytically processed by viral proteases into functional proteins, including the cylindrical inclusion protein, coat protein (CP), viral protein genome-linked, nuclear inclusion-a protease, etc. Some of these proteins orchestrate critical steps in viral replication, such as the assembly of viral replication complexes to ensure RNA synthesis. Newly synthesized genomic RNA is subsequently packaged into virions through interactions with CP and other components. These virions then traffic to adjacent cells via plasmodesmata (PD), mediated by viral movement proteins, and ultimately achieve systemic infection through long-distance transport in the phloem. The intact virion morphology of PVY is of importance for its phloem-mediated long-distance transport within host plants, suggesting that disrupting virion assembly may offer a strategic opportunity for antiviral intervention [11]. Nonetheless, research on PVY assembly inhibitors remains scarce, with limited studies exploring small molecules capable of interfering with CP-RNA interactions [12, 13].

**Fig. 1:**
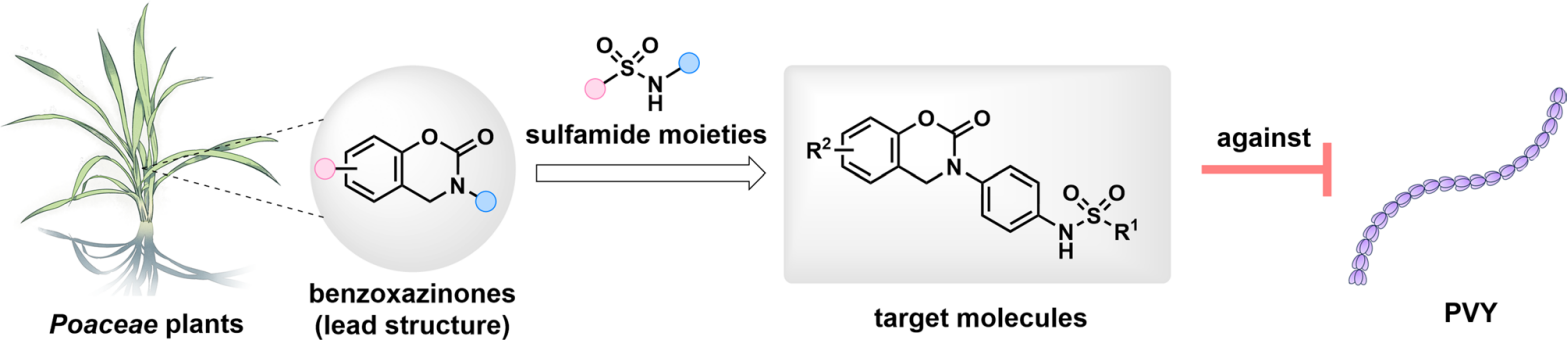
Design and synthesis of *Poaceae*-based antiviral compounds against PVY

Benzoxazinones, a class of allelopathic secondary metabolites predominantly found in gramineous plants (*Poaceae*), serve as defensive compounds against pathogenic microorganisms and competing plants [14]. These compounds exhibit diverse bioactivities like antifungal, phytotoxic, and insecticidal properties, and have garnered significant attention in agrochemical design, but relatively few studies have documented their antiviral properties [15–18]. Thus, the present employs benzoxazinone as a lead scaffold and introduces sulfonamide moieties via molecular hybridization strategy [19]. A series of novel hybrid molecules are designed, synthesized, and systematically evaluated for their anti-PVY efficacy (Fig. 1). Furthermore, their impacts on viral movement and assembly are investigated to elucidate potential mechanisms of action.

## Materials and methods

### Chemicals and instruments

All chemical reagents and solvents utilized in this study were commercially sourced and employed without further purification. Nuclear magnetic resonance spectra (^1^H, ^13^C, ^19^F) were recorded on either a Bruker Ascend-400 spectrometer (Bruker, Germany) or JEOL ECX-500 system (JEOL, Japan) using deuterated dimethyl sulfoxide as solvent. High-resolution mass spectrometry analyses were performed on an LTQ Orbitrap XL mass analyzer (Thermo Fisher Scientific, USA). Phase transition temperatures were determined using a SGWX-4B melting point apparatus (Shanghai Precision Instruments, China). Protein purification of PVY coat protein was conducted with an AKTA purifier UPC10 chromatography system (Cytiva, USA). Biomolecular interaction analyses were carried out using an Octet RED96 bio-layer interferometry platform (Sartorius, Germany). Fluorescent imaging of PVY-GFP infected *Nicotiana benthamiana* (*N. benthamiana*) leaves was accomplished with a LSM 880 confocal microscope (Zeiss, Germany), while viral particle morphology characterization was performed using a Talos F200C transmission electron microscope (Thermo Fisher Scientific, USA).

### General procedures for the preparation of the intermediate compounds A1-A11

The Hinsberg reaction [20] was conducted by dissolving p-phenylenediamine in dichloromethane (25 mL) with triethylamine as base. A sulfonyl chloride derivative was dissolved separately in dichloromethane (10 mL). Under ice-cooling, the sulfonyl chloride solution was gradually introduced into the diamine solution. The reaction system was then warmed to ambient temperature with continuous stirring until completion. After confirming reaction completion by TLC monitoring, dichloromethane was evaporated under vacuum. The residue was treated with chilled water under stirring for 10 h at room temperature. The formed precipitate was filtered under reduced pressure, air-dried, and chromatographically purified for subsequent use.

### General procedures for the preparation of the intermediate compounds C1-C34

Compounds A1-A11 and salicylaldehyde derivatives B1-B5 were charged into anhydrous methanol (20 mL). Acetic acid catalysis enabled Schiff base formation through 10-minute room-temperature agitation. Subsequent introduction of sodium cyanoborohydride achieved imine reduction to secondary amine products [21]. TLC-confirmed reaction completion triggered methanol elimination via rotary evaporation. The crude material underwent extraction with ethyl acetate (3 × 20 mL) against saturated sodium bicarbonate solution. Combined organic phases were dehydrated with anhydrous sodium sulfate prior to solvent removal, yielding chromatographically purified intermediates for downstream syntheses.

### General procedures for the preparation of the titlecompounds L1 − L34

A solution of compounds C1-C34 in dichloromethane (20 mL) was activated with catalytic DMAP under ambient stirring (3 min). Following controlled addition of *N*,*N*’-carbonyldiimidazole, the reaction proceeded at 25 °C with TLC monitoring. Post-reaction workup involved brine washing (3 × 15 mL) of the organic phase. The separated dichloromethane layer was adsorbed onto chromatographic silica gel through solvent evaporation, enabling direct dry-load purification via flash chromatography to isolate final products [22].

### Plant material

All plant seeds, including *Chenopodium amaranticolor* (*C. amaranticolor*), *N. benthamiana* and *Nicotiana tabacum* cv. K326 (*N. tabacum*), used in the experiments were supplied by the State Key Laboratory of Green Pesticide, Guizhou University.

### Virus extraction

PVY-infected *N. tabacum* leaves were cryogenically pulverized in liquid nitrogen. Leaf tissue homogenate preparation employed 0.5 M phosphate buffer (pH 7.4) at 2× mass/volume ratio, followed by organic extraction with chloroform/n-butanol (1:9 v/v). The lysate was clarified through multilayer cheesecloth filtration and subjected to differential centrifugation (8,000 × g, 20 min, 4 °C). The clarified supernatant underwent virion precipitation with Triton X-100 (1% v/v), PEG 6000 (40 g/L), and NaCl (0.2 M) during 6 h incubation. Dual centrifugation cycles (20,000 × g, 2 h, 4 °C) yielded viral pellets that were resuspended in 5 mL PBS (10 mM, pH 7.4) for overnight hydration. Final purification was achieved by pelleting cellular debris (10,000 × g, 20 min, 4 °C), retaining the supernatant containing enriched PVY virions [23].

### Antiviral activity assay

The antiviral efficacy (including curative, protective, and inactivating activities) of target compounds against PVY was evaluated using *C. amaranticolor* as a local lesion host according to established protocols [24]. The PVY strain (from our laboratory repository) was routinely propagated in *N*. *tabacum.* under controlled conditions prior to bioassays.

### Curative activity of compounds against PVY

Abrasive particles were evenly dusted onto *C. amaranticolor* leaves. Diluted PVY suspension was applied to the leaf surface, and mechanical friction between leaves was performed to ensure thorough viral contact. After inoculation, plants were maintained for 1.5 h, followed by rinsing with water to remove residual carborundum. Leaves were air-dried, and 500 µg/mL test compound solution was quantitatively applied to the right half of each leaf using a brush, while the left half was treated with a 1% Tween 80 solution as a blank control. Treated plants were incubated in a growth chamber (25 °C, 10,000 lx). After a 5–7-day latent period, characteristic necrotic lesions in each treatment zone were counted. Three independent biological replicates were performed, and the virus inhibition rate was calculated based on mean lesion numbers [24].

### Protective activity of compounds against PVY

A 500 µg/mL compound solution and a 1% Tween 80 control solution were prepared. The test compound solution was uniformly brushed onto the right half of *C. amaranticolor* leaves, while the left half received the control solution. Plants were pre-incubated in a growth chamber (25 °C, 10,000 lx) for 24 h. Subsequently, leaves were dusted with 200–300 mesh carborundum, and a 0.01 M PBS-diluted PVY suspension was frictionally inoculated by gently rubbing leaves together. Post-inoculation, plants were held for 1.5 h, rinsed to remove carborundum, and returned to the growth chamber. Necrotic lesions were quantified after 5–7 days. Three biological replicates were conducted, and the virus inhibition rate was determined using mean lesion counts [24].

### Inactivation activity of compounds against PVY

A 50 mL centrifuge tube was used to mix 1:1 (v/v) 1000 µg/mL compound solution with 2× working concentration PVY stock. The mixture was vortexed thoroughly and reacted for 30 min. The reaction solution was then applied to the right half of leaves, while the left half received an equal volume of untreated PVY stock as a control. Post-inoculation, plants were maintained for 1.5 h, rinsed to remove residual carborundum, and air-dried. Plants were incubated in a growth chamber (25 °C, 10,000 lx). Necrotic lesions were counted after 5–7 days. Three independent replicates were performed, and virus inhibition rates were calculated based on mean lesion numbers [24].

#### Molecular docking and molecular dynamics simulation

The crystal structure of PVY CP (PDB ID: 6HXX, Chain A) was retrieved from the RCSB Protein Data Bank. Using AutoDock Tools 1.5.7, molecular docking was performed to analyze interactions between compound L5 and PVY CP, following established protocols [25].

### Plasmid construction for prokaryotic expression

The coding sequence of PVY CP was cloned into the expression vector pET-32a(+)[26], and the resulting plasmid was designated as pET-PVY-CP^wt^. Based on pET-PVY CP^wt^, site directed mutagenesis was performed to substitute the codon for valine at position 211 with alanine, generating the plasmid pET-PVY CP^V211A^.

### Expression and purification of PVY CP^wt^ and PVY CP^V211A^

The constructed PVY CP and PVY CP^V211A^ expression plasmids were transformed into *Escherichia coli* BL21(DE3) competent cells. The transformed cells were plated on LB agar plates containing 50 µg/mL ampicillin and incubated to select positive single colonies. A single colony was inoculated into liquid LB medium for large-scale culture. Protein expression was induced with 1 M IPTG at low temperature for 12 h. After induction, the bacterial cells were harvested by centrifugation and lysed via sonication. The lysate was purified using a His-Trap™ HP column for affinity chromatography. The His and S tags were enzymatically cleaved using enterokinase, and the PVY CP protein was separated from the His tag using purification resin. Finally, the purified protein was identified and validated by sodium dodecyl sulfate-polyacrylamide gel electrophoresis analysis [27, 28].

### Bio-layer interferometry assays

The PVY CP^WT^ and PVY CP^V211A^ proteins were biotinylated and immobilized onto SSA biosensors. The binding affinity between compound L5 and PVY CP^WT^ and PVY CP^V211A^ was measured on an Octet RED 96 system [29].

### Construction of infective clones

A reported GFP-containing pCamPVY-GFP plasmid based on PVY isolate Guizhou (GenBank: MN381731) was employed [30]. pCamPVY^V211A^-GFP was obtained by replacing the codon of V211 in pCamPVY-GFP using site-directed mutagenesis technique [31]. The constructed plasmids were sequenced. The primer sequences required for the mutation are listed in Table S1.

### Transient expression of *Agrobacterium*

The plasmids pCamPVY-GFP and pCamPVY^V211A^-GFP were transformed into *Agrobacterium* tumefaciens strain GV3101. The transformed cells were cultured on YEP solid medium at 28 °C for 24–48 h, and positive single colonies were selected and inoculated into liquid YEP medium for large-scale culture. After centrifugation, the bacterial pellets were resuspended in MMA buffer (10 mM MgCl_2_, 10 mM MES, and 100 μM AS), adjusted to an OD_600_ of 0.5, and incubated in the dark for at least 4 h. Finally, the bacterial suspension was infiltrated into four-leaf-stage *N. benthamiana* plants. Inoculated leaves were observed, harvested, and stored at − 80 °C for further analysis [32].

### RNA extraction, cDNA synthesis, and RT-qPCR

Total RNA isolation from *Agrobacterium*-infiltrated *N. benthamiana* leaves (7 dpi) was conducted with Trizol reagent (TransGen Biotech, China) following the manufacturer’s protocols. RNA quantification was performed spectrophotometrically, with 1 µg aliquots subjected to cDNA synthesis using random hexamers and a PrimeScript RT reagent kit (Takara, China). For quantitative PCR analysis, SYBR Green master mix (Takara, China) was employed with gene-specific primers (Table S1), using the endogenous actin gene as normalization control.

### Western blot

Protein extracts were prepared from upper systemic leaves of PVY-GFP and PVY^V211A^-GFP mutants harvested at 7 dpi. Western blot was performed with commercial antibodies: primary anti-PVY CP mouse monoclonal antibody (YouLong Biotech) and HRP-conjugated goat anti-mouse IgG secondary antibody (Shenggong Biotech). Protein signals were captured through chemiluminescent detection using a ChemiDoc MP system (Bio-Rad, USA).

### Confocal microscopy

Viral movement dynamics of PVY-GFP and PVY^V211A^-GFP mutants in *N. benthamiana* were assessed through confocal microscopy imaging. *Agrobacterium*-mediated leaf infiltration with pCamPVY constructs was monitored using an LSM 880 CLSM system (Zeiss). GFP signals were acquired under 488 nm excitation with 520–540 nm emission filters. Image quantification was conducted with ZEN 2.1 software (Zeiss) for spatial-temporal analysis of viral spread patterns.

### Transmission electron microscope

PVY virions were deposited on 200-mesh carbon-coated copper grids, followed by negative staining with 1% phosphotungstic acid (pH 7.4) and ambient drying. Ultrastructural analysis was conducted using TEM (Talos F200C, Thermo Fisher Scientific) operated at 120 kV accelerating voltage.

## Results

### Chemistry

**Fig. 2:**
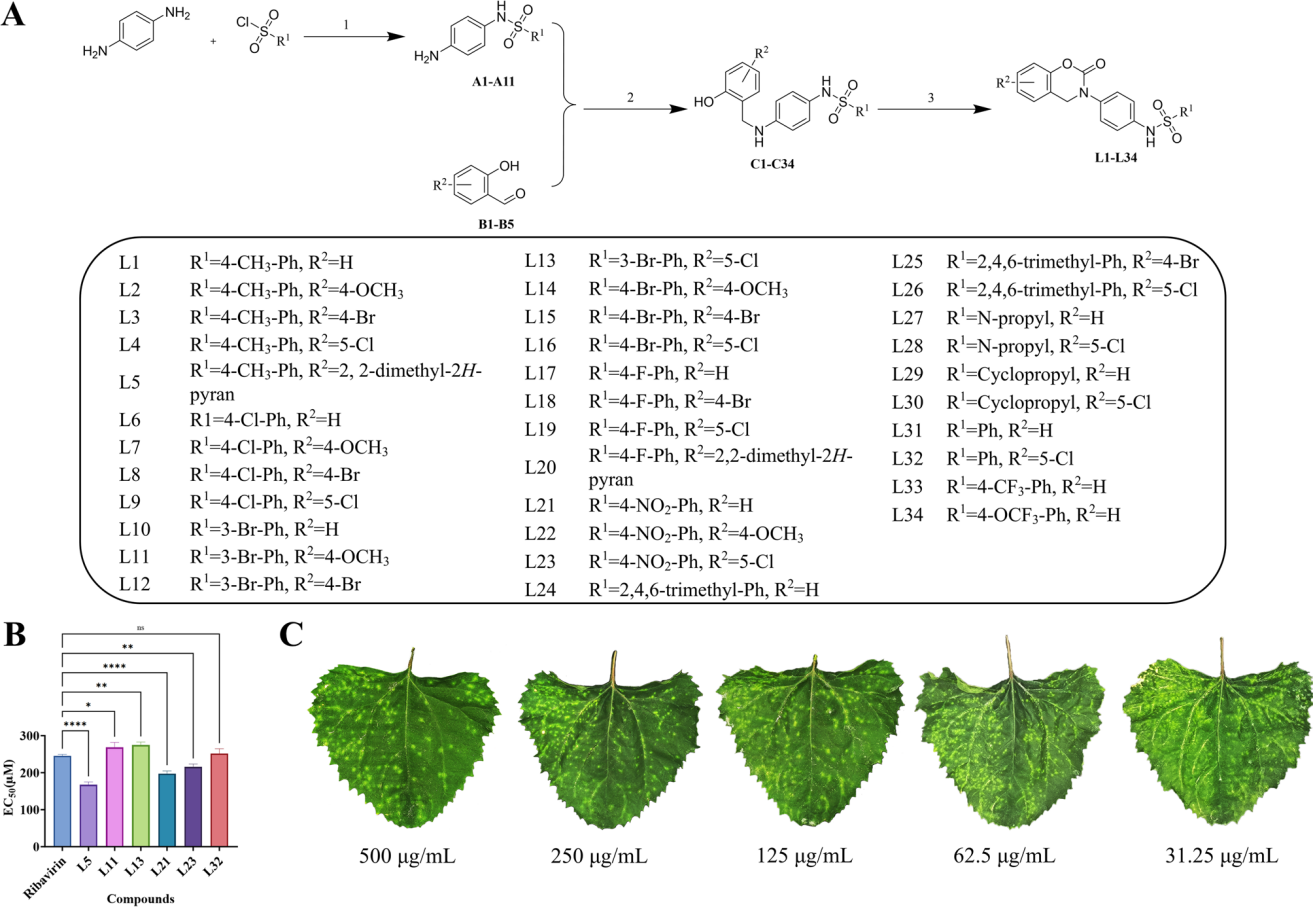
Synthetic route of the target compound and inactivation EC_50_ values of partial compounds. **A** Manufacturing pathways for the target molecules. Reagent and conditions: (1) TEA, DCM, 0℃ 30 min then at rt 3 h (2) NaBH_3_CN, CH_3_COOH, at rt 4 h (3) DMAP, CDI, at rt 30 min. **B** The EC₅₀ values of compound L5, L11, L13, L21, L23, L32 against PVY, with ribavirin as the reference drug. Data are presented as mean ± SD from three biological replicates per treatment, with statistical significance indicated by different letters (*p* < 0.05, one-way ANOVA). **C** The inactive activity of L5 against PVY at different concentrations

The title compounds possessed a benzoxazinone skeleton structure with a wide range of biological activities. A sulfonamide unit, which exhibited antiviral activity, had been introduced as a bridging moiety. A straightforward three-step synthetic route to the target compounds was developed. First, intermediates A1-A11 were synthesized via the Hinsberg reaction. Next, the Schiff base intermediate was reduced by sodium cyanoborohydride to afford secondary amines, yielding intermediates C1-C34. Finally, under CDI-mediated conditions, the target compounds L1-L34 were obtained via formylation-cyclization (Fig. 2A). All target compounds were fully characterized by ¹H NMR, ¹³C NMR, ¹⁹F NMR, and HRMS (available in Supplementary Material 1).

### Anti-PVY bioassay

The antiviral efficacy against PVY was evaluated at 500 µg/mL through three administration modalities: protection, therapy, and viral inactivation. *C. amaranticolor* was used as the local lesion assay host, with ribavirin as the positive control [33]. As shown in Table 1, most target compounds exhibited anti-PVY activity, with some compounds showing even higher activity than the positive control. To further assess the inactivation activity, the EC_50_ values of compounds L5, L11, L13, L21, L23, and L32 were determined and presented in a bar chart (Fig. 2B) and Table 2. Compound L5 demonstrated the lowest EC_50_ value (169.4 µg/mL), with its concentration-dependent activity shown in Fig. 2C. Preliminary structure-activity relationships revealed that phenyl substitutions at R^1^ conferred better activity than aliphatic substitutions (L31 > L27, L29; L32 > L28, L30), and methyl groups on the phenyl ring were more favorable than halogens for antiviral activity (L4 > L9, L16, L19).

**Table 1:** Antiviral activities of the target compounds against PVY

Comp.		Protective activity (%)^a^	Curative activity (%)^a^	Inactivating activity (%)^a^
L1	R^1^ = 4-CH_3_-Ph, R^2^ = H	45.23 ± 3.24	49.93 ± 2.40	62.11 ± 4.13
L2	R^1^ = 4-CH_3_-Ph, R^2^ = 4-OCH_3_	30.12 ± 3.39	28.14 ± 2.45	41.75 ± 4.24
L3	R^1^ = 4-CH_3_-Ph, R^2^ = 4-Br	43.33 ± 2.72	45.46 ± 1.21	58.16 ± 4.45
L4	R^1^ = 4-CH_3_-Ph, R^2^ = 5-Cl	47.23 ± 3.24	48.35 ± 5.12	67.34 ± 3.36
L5	R^1^ = 4-CH_3_-Ph, R^2^ = 2, 2-dimethyl-2*H*-pyran	53.45 ± 1.64	51.78 ± 6.34	73.99 ± 2.08
L6	R1 = 4-Cl-Ph, R^2^ = H	52.18 ± 2.56	55.14 ± 2.08	54.21 ± 4.32
L7	R^1^ = 4-Cl-Ph, R^2^ = 4-OCH_3_	46.67 ± 4.67	47.24 ± 2.12	63.12 ± 3.29
L8	R^1^ = 4-Cl-Ph, R^2^ = 4-Br	37.13 ± 3.24	40.19 ± 2.24	63.69 ± 1.98
L9	R^1^ = 4-Cl-Ph, R^2^ = 5-Cl	48.35 ± 5.12	40.90 ± 3.14	61.12 ± 4.29
L10	R^1^ = 3-Br-Ph, R^2^ = H	41.15 ± 4.44	50.74 ± 2.62	37.31 ± 3.15
L11	R^1^ = 3-Br-Ph, R^2^ = 4-OCH_3_	47.25 ± 4.42	57.5 ± 3.24	68.23 ± 3.14
L12	R^1^ = 3-Br-Ph, R^2^ = 4-Br	53.55 ± 3.24	44.5 ± 2.45	57.66 ± 5.12
L13	R^1^ = 3-Br-Ph, R^2^ = 5-Cl	45.3 ± 3.21	44.23 ± 4.34	69.24 ± 4.23
L14	R^1^ = 4-Br-Ph, R^2^ = 4-OCH_3_	47.61 ± 5.12	41.24 ± 2.67	61.33 ± 2.72
L15	R^1^ = 4-Br-Ph, R^2^ = 4-Br	48.15 ± 3.12	50.95 ± 6.12	62.18 ± 1.75
L16	R^1^ = 4-Br-Ph, R^2^ = 5-Cl	58.13 ± 3.21	39.7 ± 2.24	60.72 ± 3.14
L17	R^1^ = 4-F-Ph, R^2^ = H	34.5 ± 3.34	47.12 ± 4.32	42.85 ± 4.24
L18	R^1^ = 4-F-Ph, R^2^ = 4-Br	48.96 ± 2.72	49.40 ± 5.12	66.61 ± 5.12
L19	R^1^ = 4-F-Ph, R^2^ = 5-Cl	39.15 ± 3.12	36.12 ± 2.77	39.32 ± 3.12
L20	R^1^ = 4-F-Ph, R^2^ = 2,2-dimethyl-2*H*-pyran	29.21 ± 1.45	48.44 ± 3.78	55.72 ± 2.42
L21	R^1^ = 4-NO_2_-Ph, R^2^ = H	48.5 ± 4.31	49.35 ± 5.24	68.67 ± 3.14
L22	R^1^ = 4-NO_2_-Ph, R^2^ = 4-OCH_3_	57.13 ± 5.43	58.41 ± 5.34	61.24 ± 5.23
L23	R^1^ = 4-NO_2_-Ph, R^2^ = 5-Cl	48.07 ± 5.12	55.33 ± 2.42	69.15 ± 6.45
L24	R^1^ = 2,4,6-trimethyl-Ph, R^2^ = H	59.21 ± 2.12	32.56 ± 3.32	55.44 ± 2.12
L25	R^1^ = 2,4,6-trimethyl-Ph, R^2^ = 4-Br	37.66 ± 6.21	36.53 ± 4.11	52.46 ± 5.23
L26	R^1^ = 2,4,6-trimethyl-Ph, R^2^ = 5-Cl	47.55 ± 3.14	51.07 ± 5.12	63.25 ± 3.66
L27	R^1^ = *N*-propyl, R^2^ = H	46.66 ± 6.23	48.44 ± 5.31	54.33 ± 3.24
L28	R^1^ = *N*-propyl, R^2^ = 5-Cl	48.84 ± 4.12	43.13 ± 1.56	61.12 ± 4.12
L29	R^1^ = Cyclopropyl, R^2^ = H	42.15 ± 2.56	38.14 ± 4.24	49.44 ± 5.31
L30	R^1^ = Cyclopropyl, R^2^ = 5-Cl	51.46 ± 4.14	42.12 ± 3.23	58.36 ± 5.24
L31	R^1^ = Ph, R^2^ = H	45.56 ± 4.23	49.77 ± 4.50	55.98 ± 3.14
L32	R^1^ = Ph, R^2^ = 5-Cl	48.14 ± 4.32	44.11 ± 3.25	70.86 ± 2.22
L33	R^1^ = 4-CF_3_-Ph, R^2^ = H	54.06 ± 4.11	50.33 ± 3.12	65.13 ± 3.87
L34	R^1^ = 4-OCF_3_-Ph, R^2^ = H	47.5 ± 6.21	40.24 ± 1.45	63.63 ± 5.12
Ribavirin^b^	-	46.3 ± 3.21	49.23 ± 2.12	64.95 ± 3.21

**Table 2:** Calculated EC_50_ values based on the inactivation activity of the compounds

Compounds	EC_50_ for inactivating activity (µg/mL)
L5	169.4 ± 7.23
L11	268.3 ± 2.15
L13	284.4 ± 9.34
L21	205.7 ± 11.25
L23	214.3 ± 9.52
L32	253.5 ± 15.34
Ribavirin^b^	244.7 ± 4.37

^a^Average of three replicates

^b^Ribavirin used as positive control

### Molecular docking

**Fig. 3:**
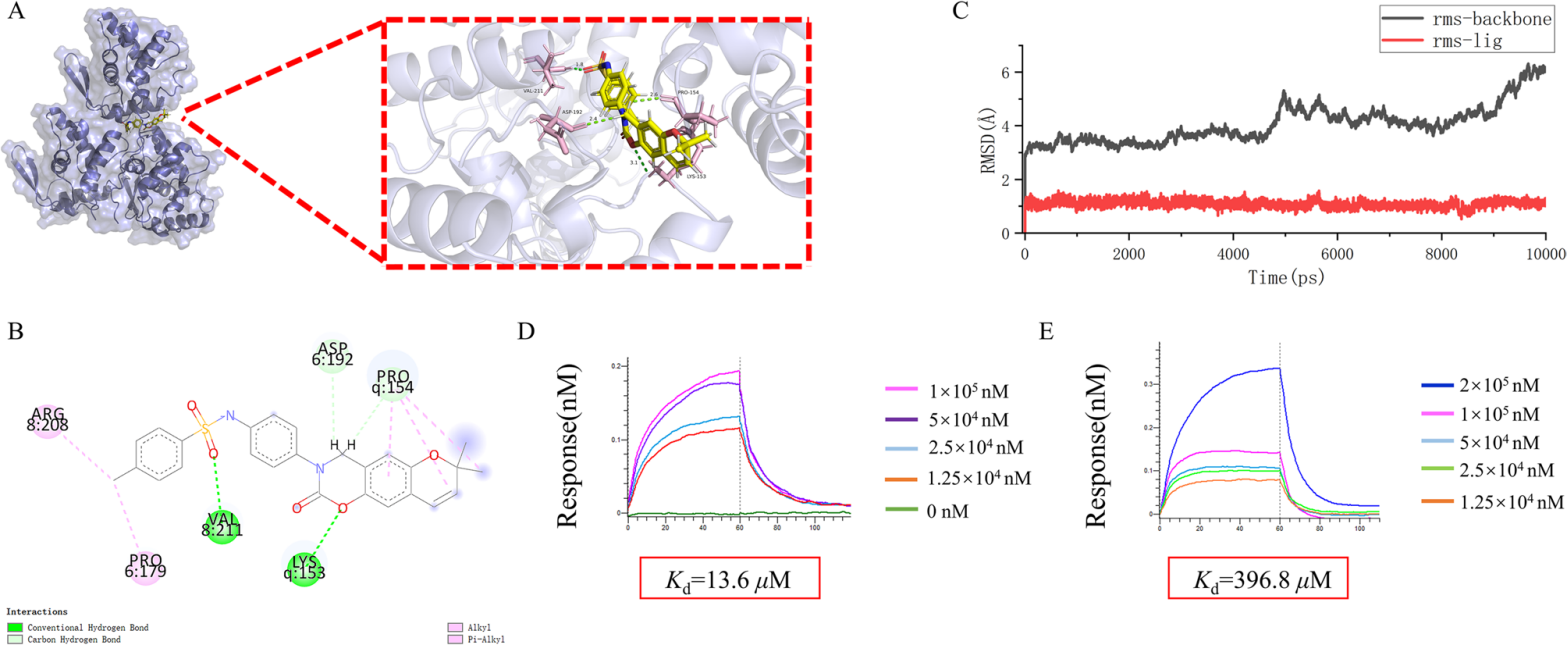
In vitro binding assay for L5 with PVY CP. **A** Molecule docking results of L5 with PVY CP. **B** 2D ligand-protein interaction diagram of compound L5 bound to PVY CP. **C** RMSD analysis of compouL5 PVY CP. (**D**,** E**) Binding affinity of compound L5 to PVY^WT^ and PVY CP^V211A^ was determined by bio-layer interferometry assay

Based on the anti-PVY activity evaluation, we found that compound L5 exhibited excellent activity. To verify its interaction with PVY CP and identify binding sites, molecular docking was performed using Autodock (Fig. 3A). The docking score of −6.61 kcal/mol indicated stable binding between L5 and PVY CP. Detailed structural analysis identified multiple interaction modes: hydrogen bonds with Val211 (1.8 Å) and Lys153 (3.1 Å), ionic interactions with Pro154 (2.6 Å) and Asp192 (2.4 Å), along with hydrophobic contacts involving Pro179 and Arg208 (Fig. 3B). Molecular dynamics simulations (10,000 ps, Ledock) further confirmed the complex’s stability, showing rapid convergence to equilibrium with minimal RMSD fluctuations (Fig. 3C). These results collectively establish that L5 achieves robust binding within PVY CP’s conserved region through multiple interaction forces, with the particularly strong hydrogen bond to Val211 emerging as the critical binding determinant for its antiviral activity.

### Effect of mutations at the PVY CP V211 site on binding

To verify that V211 is the key amino acid residue of PVY CP for compound L5, an in vitro validation method was used to determine the difference in binding strength between L5 and PVY CP^WT^ versus PVY CP^V211A^ via Bio-Layer Interferometry (BLI). As shown in the Fig. 3D and E, the results indicate that compound L5 exhibits strong affinity for PVY CP^WT^ with a *K*_d_ value of 13.6 *µ*M. Additionally, the interaction affinity between the target compound and PVY CP^V211A^ was measured, yielding a *K*_d_ value of 396.8 *µ*M, representing a 29.8-fold difference. These experimental results demonstrate that mutation of the PVY CP V211 site significantly weakens the binding strength of L5 to PVY CP, suggesting that valine at position 211 is likely the critical amino acid residue for L5’s action on PVY CP.

### Effect of PVY CP^V211A^ locus on PVY invasion

**Fig. 4:**
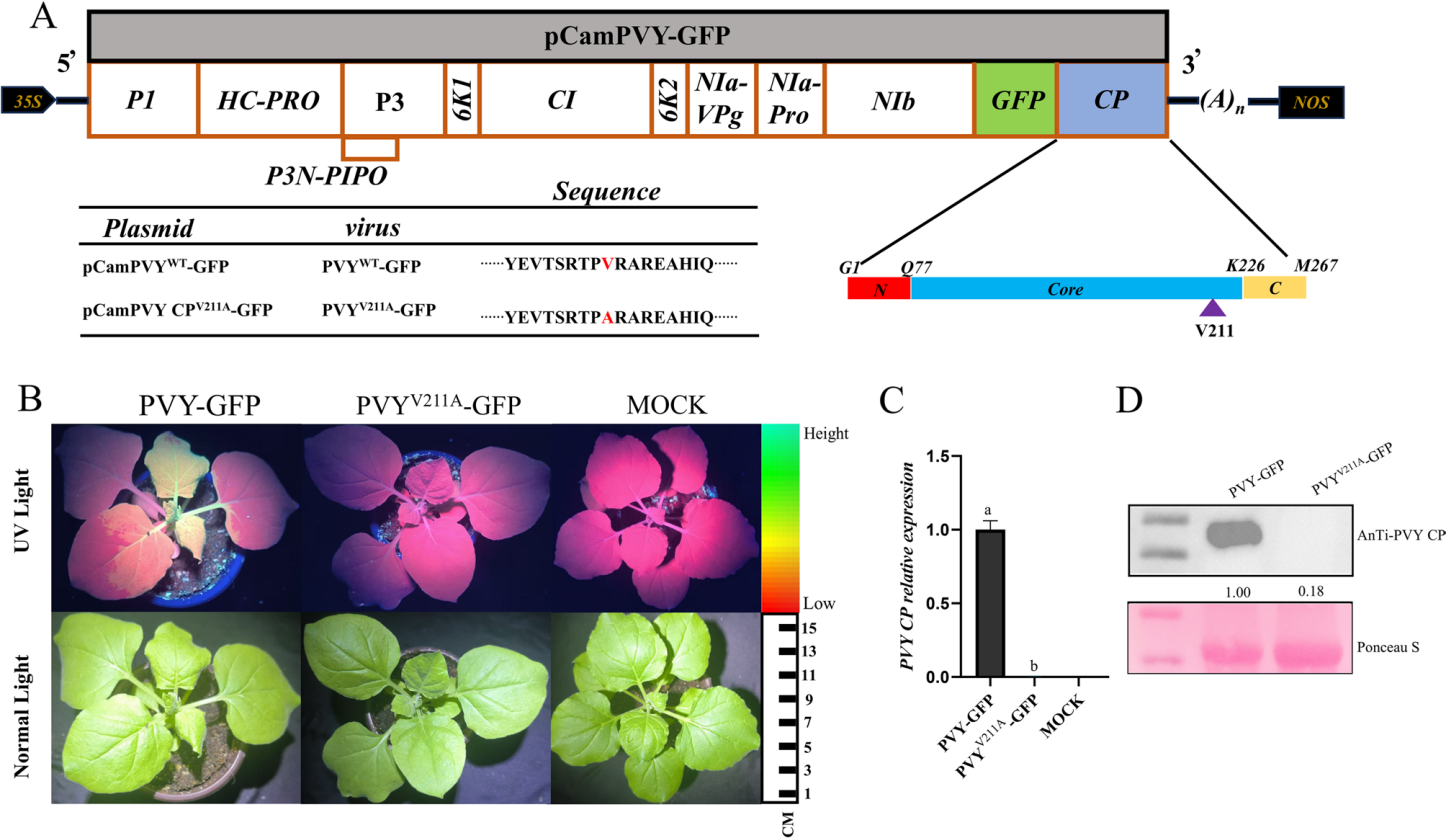
Effect of mutation on Val 211 in CP on PVY infection. **A** Illustrative representation of the pCamPVY-GFP genome structure, highlighting the Val 211 residue (marked by purple arrows) within the core domain of PVY CP. **B** Symptoms (lower panel) and green fluorescence (upper panel) under UV light of *N. benthamiana* infiltrated by wild-type and mutated PVY. **C** The accumulation levels of viral RNAs in systemically infected leaves of *N. benthamiana*. inoculated with PVY-GFP and PVY CP^V211A^-GFP were analyzed by RT-qPCR at 7 dpi. RT-qPCR data were normalized using Actin as an internal control. Data arepresented as mean ± SD from three biological replicates per treatment, with statistical significance indicated by different letters (*p* < 0.05, one-way ANOVA). **D** Western blot analysis was performed to detect the accumulation levels of viral proteins in systemically infected leaves of *N. benthamiana*. inoculated with PVY-GFP and PVY CP^V211A^-GFP at 7 dpi. Ponceau S staining of RuBisCO was used as a loading control

To functionally characterize V211 in PVY CP as the target site of L5, we conducted in vivo validation. The infectious clone pCamPVY-GFP was engineered, with PCR-mediated site-directed mutagenesis generating pCamPVY^V211A^-GFP (Fig. 4A). Both constructs were transformed into *Agrobacterium* GV3101 and infiltrated into 4-6-week-old *N*. *benthamiana*. At 7 dpi, systemic leaves infected with wild-type PVY-GFP showed intense GFP signals, while V211A mutant-infected plants displayed only background fluorescence (Fig. 4B). Western blot and RT-qPCR confirmed abundant PVY CP accumulation in wild-type infections versus trace levels in V211A mutants (Fig. 4C, D). These data demonstrate that V211 mutation severely compromises viral accumulation. Collectively, our results establish V211 as an essential residue for both **L5** binding and systemic PVY infection in plants.

### Effect of the PVY CP V211 locus on the movement of PVY in cells

**Fig. 5:**
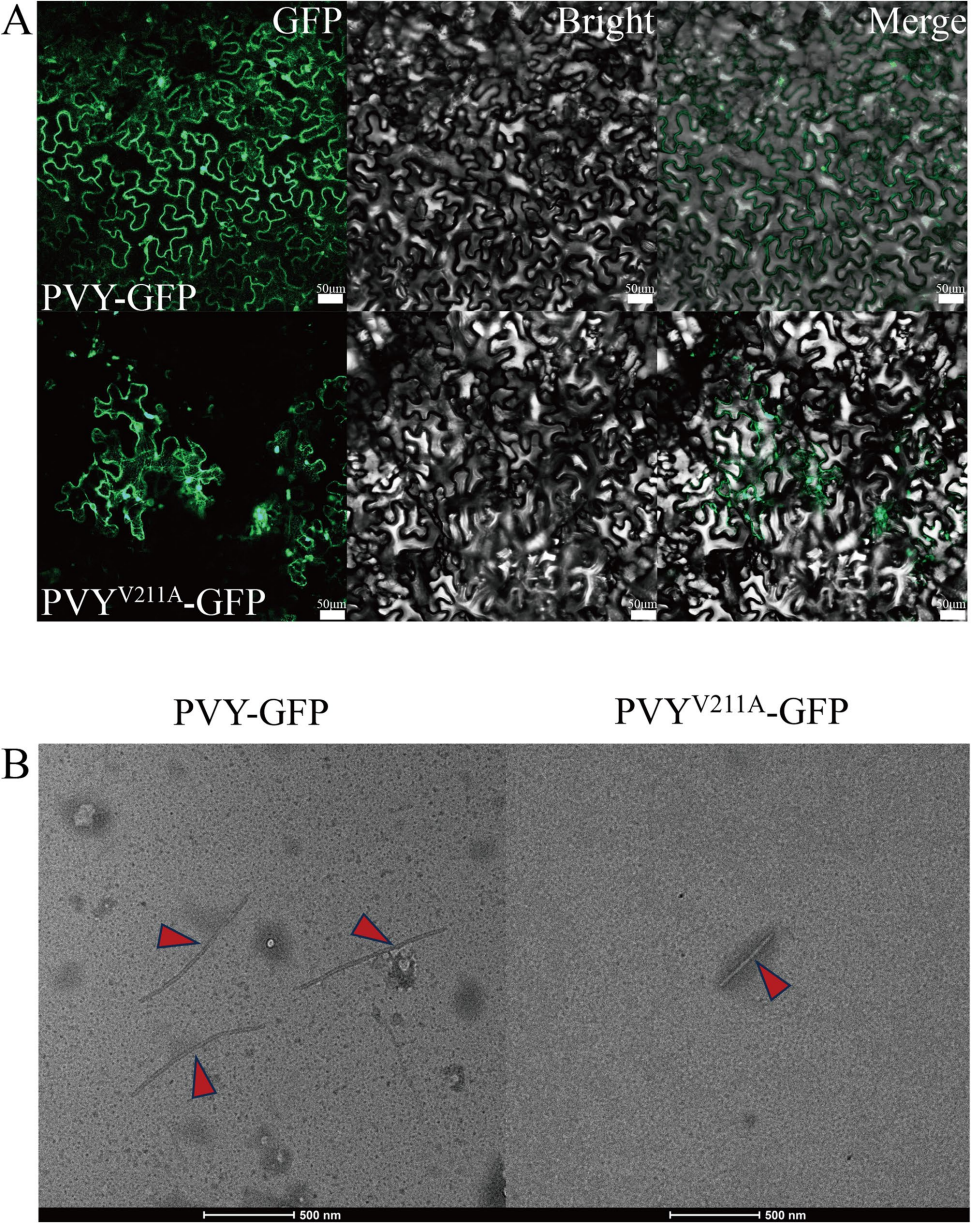
Impact of the Val211 amino acid residue mutation in PVY CP on viral movement and assembly. **A** Detection of intercellular movement of wild-type and V211-mutant PVY strains in infiltrated leaves of *N. benthamiana.* at 5 dpi. (Scale bar = 50 μm). **B** Particles of PVY-GFP, PVY^V211A^-GFP under transmission electron microscope (Scale bar = 500 nm)

The systemic movement of PVY requires coordinated plasmodesmal and phloem-mediated transport, a process modulated by coat protein functions [34–36]. To assess V211 residue effects on viral trafficking, *Agrobacterium* suspensions (OD_600_ = 0.0005, 1:1000 dilution) carrying PVY-GFP or PVY^V211A^-GFP were infiltrated into 4-week-old *N. benthamiana*. Fluorescent imaging analysis at 5 dpi revealed distinct movement patterns: PVY^V211A^-GFP exhibited diffuse fluorescence patterns (Fig. 5A) [36]demonstrating preserved intercellular mobility despite V211 substitution. These findings suggest the CP mutation does not impair short-distance viral spread in somatic tissues, maintaining PVY infectivity.

### Effect of the PVY CP V211 locus on PVY assembly

Viral particle assembly represents the final stage of viral infection within host cells [37]. The coat protein of PVY facilitates this process through self-interaction or binding to (+)ssRNA[38]. To assess the role of V211 in PVY assembly, wild-type PVY-GFP and mutant PVY^V211A^-GFP were agroinfiltrated into mature leaves of *N. benthamiana*. Viral particles were subsequently purified using established protocols, stained with 1% phosphotungstic acid, and visualized via transmission electron microscopy. TEM analysis revealed that PVY^V211A^-GFP particles were significantly shorter in length compared to wild-type PVY particles (Fig. 5B) These results demonstrate that V211 in PVY CP is a critical residue governing proper viral assembly.

## Discussion

Viral particle assembly represents a terminal yet critical phase in the viral life cycle, constituting the ultimate prerequisite for intercellular translocation [10]. This maturation process fundamentally determines virion stability. Our recent investigations have shown that targeting the degradation of viral proteins can be achieved by disrupting their interaction with RNA [39]. While mature virion formation substantially potentiates both cell-to-cell movement and systemic spread of potyviruses, it should be emphasized that residual mobility persists in ribonucleoprotein complexes [40]. Notably, Wan et al. provided evidence for xylem-mediated long-distance transport of Turnip mosaic virus replication complex [41]. However, it is undeniable that the CP plays a multifunctional role in mediating vector transmission, movement, assembly, and host interaction, making anti-potyvirus strategies targeting CP widely recognized [37].

Emerging evidence suggests that perturbation of viral protein functionality through small molecule interventions represents a promising antiviral paradigm [42, 43]. A plethora of natural products have been explored as lead structures for the derivation of antiviral purposes [12, 13]. Particularly, dithioacetal-modified vanillin derivatives have demonstrated broad-spectrum antiviral efficacy against cucumber mosaic virus, PVY, and tobacco mosaic virus [44]. The commercial antiviral agent dufulin, derived from *α*-aminophosphonic acid analogs, has shown significant protective potential against viral phytopathogens [45, 46]. Despite these advances, benzoxazinone-based antiviral agents remain underrepresented. Although Xue et al. documented moderate anti-TMV activity in myricetin-benzoxazinone hybrids, the benzoxazinone moiety served as a secondary scaffold with undefined mechanistic underpinnings [17].

In the present investigation, we strategically employed benzoxazinone as the core scaffold, incorporating antimicrobial sulfonamide moieties to generate novel target compounds (Fig. 1B). Among these derivatives, compound **L5** exhibited superior inactivation efficacy against PVY (Table 1), a phenomenon typically associated with its direct effect on viral functional components. Contrastingly, protective activity generally correlates with phytochemical-induced immune activating [43]. A recent study demonstrated that phthalide derivatives can act on PVY Nia to exert curative effect, inhibiting viral replication [32]. Molecular docking and binding affinity analyses preliminary confirmed the interaction between **L5** with the V211 residue of PVY CP. Given CP’s central role in virion morphogenesis, viral trafficking, and systemic infection, we systematically investigated its antiviral mechanisms. *Agrobacterium*-mediated infectivity assays revealed near-complete loss of systemic spread capacity in the V211A mutant (Fig. 4C, D). Confocal microscopy analyses detected comparable multicellular fluorescence patterns in wild-type and mutant strains, indicating preserved cell-to-cell movement capacity (Fig. 5A). The attenuated fluorescence intensity observed in V211A mutants may reflect compensatory ribonucleoprotein complex-mediated movement mechanisms, albeit significantly less efficient than particle-dependent translocation. TEM further demonstrated substantial reduction and structural aberrations in V211A viral particles, suggesting impaired assembly process (Fig. 5B).

## Conclusion

In summary, this study developed a novel benzoxazinoid-derived compound L5, structurally derived from secondary metabolites of *Poaceae* plants. In virucidal assays, L5 exhibited a lower EC_50_ value compared to the commercial control ribavirin. Molecular docking, dynamics simulations, and bio-layer interferometry identified V211 as the critical binding site, with the V211A mutant showing reduction in viral particle quantity and shorter particle length compared to wild-type PVY, while unimpaired viral cell-to-cell movement suggested L5’s interference with CP-RNA assembly rather than viral movement processes. These results establish L5 as a promising lead compound for rationally designing CP-dependent virion assembly inhibitors, providing a strategic framework for combating PVY and related plant viruses through targeted disruption of viral encapsidation mechanisms.

## Supplementary Information

Supplementary Material 1

## Data Availability

All of the datasets are included within the article and its additional files.

## Abbreviations

NMR: Nuclear magnetic resonance
HRMS: High-resolution mass spectra
GFP: Green fluorescent protein
DCM: Dichloromethane
TLC: Thin layer chromatography
PEG: Polyethylene glycol
PDB: Protein data bank
RCSB: Research collaboratory for structural bioinformatics
LB: Luria-bertan
IPTG: Isopropyl*β*-*D*-thiogalactoside
SSA: Streptococcal superantigen
SDS-PAGE: Sodium dodecyl sulfate-polyacrylamide gel electrophoresis
YEP: Yeast extract peptone
MES: 2- (*N*-Morpholino) ethanesulfonic acid
AS: Acetosyringone
OD: Optical density
KD: Dissociation constant
RMSD: Root mean square deviation
RT-qPCR: Reverse transcription quantitative PCR
WB: Western blot
ssRNA: Single-stranded ribonucleic acid
RNA: Ribonucleic Acid
TMV: Tobacco Mosaic Virus
PBS: Phosphate-Buffered Saline
TEA: Triethylamine
EC_50_: Half Maximal Effective Concentration
CDI: *N*,*N*'-Carbonyldiimidazole
DMAP: 4-Dimethylaminopyridine
