# Supplementary material for: Discovery of Poaceae-based virion-assembly inhibitor for managing potato virus Y
Source: BMC Plant Biol. 2025 Oct 6;25:1314. doi: 10.1186/s12870-025-07404-x (PMC12502192; doi:10.1186/s12870-025-07404-x)
Supplement: Supplementary file 1 — Supplementary Material 1 [file 12870_2025_7404_MOESM1_ESM.pdf]

Supplementary Material 1

Discovery of *Poaceae*-based virion-assembly inhibitor for managing potato virus Y

Yuefei Long[1], Chunni Zhao[2], Huan Wu[3], Zhongjie Shen[4], Baoan Song*, and Deyu Hu*

[a] *State Key Laboratory of Green Pesticide, Center for R&D of Fine Chemicals of Guizhou University, Guiyang 550025, P. R. China.*

Contents

1. Table S1 **3**

2. Characterizations of products **4**

3. ^{1}H NMR, ^{13}C NMR , ^{19}F NMRand HRMS data **16**

Table S1. Primers used in this study, related to Figure 4A and 4C.

primer	Sequences 5'→3'
PVY CP^{V211A}-GFP-F	ACCAGCGAGGGCTAGGGAAGCGCACATTCAAA
PVY CP^{V211A}-GFP-R	GCCCTCGCTGGTGTTCGTGATGTGACCTCATAAA
qPCR-PVY CP-F	TGGCGAGGTTCCATTTTCA
qPCR-PVY CP-R	CATAGGAGAAACTGAGATGCCAACT
qPCR-Actin-F	CACACTGGAGTGATGGTTGG
qPCR-Actin-R	GGTGTGGTGCCAAATCTTCT

2. Characterizations of products

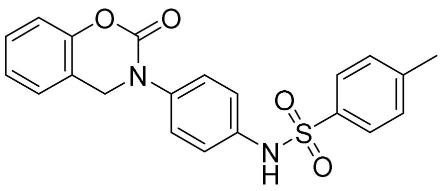

4-methyl-*N*-(4-(2-oxo-2*H*-benzo[*e*][1,3]oxazin-3(4*H*)-yl)phenyl)benzenesulfonamide(L1), Yield: 44.56%; white solid; m. p. 228.4-229.3 °C; ^{1}HNMR (500 MHz, DMSO-d_6) δ 10.41 (s, 1H, -SO_2NH-), 7.65 (d, *J* = 8.3 Hz, 2H, Ar-H), 7.32 (d, *J* = 8.2 Hz, 2H, Ar-H), 7.28 (d, *J* = 8.8 Hz, 3H, Ar-H), 7.22 (dd, *J* = 7.6, 1.6 Hz, 1H, Ar-H), 7.14 – 7.11 (m, 1H, Ar-H), 7.10 (d, *J* = 8.9 Hz, 2H, Ar-H), 7.06 (dd, *J* = 8.3, 1.1 Hz, 1H, Ar-H), 4.76 (s, 2H, -CH_2-), 2.30 (s, 3H, -CH_3). ^{13}C NMR (151 MHz, DMSO-d_6) δ 150.04, 149.81, 143.95, 138.14, 137.16, 136.80, 130.34, 129.34, 127.25, 126.93, 126.52, 124.80, 120.44, 119.35, 116.00, 50.15, 21.50. HRMS (ESI): calcd for $C_{21}H_{19}N_2O_4S$([M+H]$^+$), 395.10600; found,395.10495.

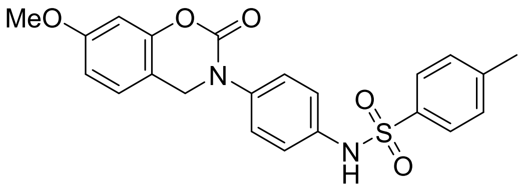

***N*-(4-(6-methoxy-2-oxo-2*H*-benzo[*e*][1,3]oxazin-3(4*H*)-yl)phenyl)-4-methylbenzenesulfonamide(L2),** Yield: 42.31%; yellow solid; m. p. 160.7-161.5 °C; ^{1}H NMR (500 MHz, DMSO-d_6) δ 10.43 (s, 1H, -SO_2NH-), 7.66 (d, J = 8.5 Hz, 2H, Ar-H), 7.32 (d, J = 7.8 Hz, 2H, Ar-H), 7.28 (d, J = 9.0 Hz, 2H, Ar-H), 7.11 (d, J = 8.4 Hz, 3H, Ar-H), 6.71 (d, J = 8.5 Hz, 1H, Ar-H), 6.67 (s, 1H, Ar-H), 4.67 (s, 2H, -CH_2-), 3.71 (s, 3H, -CH_3), 2.30 (s, 3H, -CH_3). ^{13}C NMR (126 MHz, DMSO-d_6) δ 160.18, 150.65, 149.97, 143.98, 138.25, 137.14, 136.79, 130.36, 127.27, 127.16, 126.93, 120.43, 111.14, 111.02, 101.42, 56.04, 49.84, 21.51. HRMS (ESI): calcd for $C_{22}H_{21}N_2O_5S$ ([M+H]$^+$),425.11675; found,425.11588.

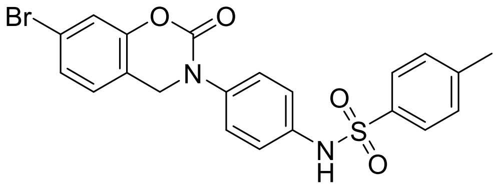

***N*-(4-(7-bromo-2-oxo-2*H*-benzo[*e*][1,3]oxazin-3(4*H*)-yl)phenyl)-4-methylbenzenesulfonamide(L3),** Yield: 70.16%; yellow solid; m. p. 188.7-190.5 °C; ^{1}H NMR (500 MHz, DMSO-d_6) δ 10.40 (s, 1H, -SO_2NH-), 7.65 (d, *J* = 8.2 Hz, 2H, Ar-H), 7.36 (s, 1H, Ar-H), 7.34 – 7.31 (m, 3H, Ar-H), 7.28 (d, *J* = 8.9 Hz, 2H, Ar-H), 7.18 (d, *J* = 8.2 Hz, 1H, Ar-H), 7.09 (d, *J* = 10.1 Hz, 2H, Ar-H), 4.72 (s, 2H, -CH_2-), 2.29 (s, 3H, -CH_3). ^{13}C NMR (151 MHz, DMSO-d_6) δ 150.61, 149.45, 143.97, 137.95, 137.13, 136.92, 130.35, 128.35, 127.63, 127.26, 126.97, 121.13, 120.42, 118.96, 118.89, 49.91, 21.51. HRMS (ESI): calcd for $C_{21}H_{18}BrN_2O_4S$ ([M+H]$^+$),473.01652; found,473.01501.

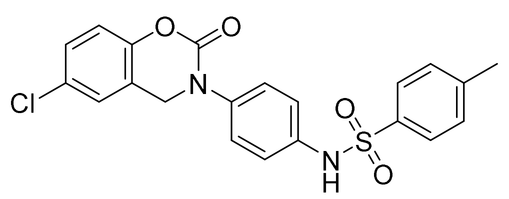

***N*-(4-(6-chloro-2-oxo-2*H*-benzo[*e*][1,3]oxazin-3(4*H*)-yl)phenyl)-4-methyl-benzenesulfonamide(L4),** Yield: 47.78%; white solid; m. p. 201.4-202.9 °C; ^{1}H NMR (500 MHz, DMSO-d_6) δ 10.42 (s, 1H, -SO_2NH-), 7.69 (d, *J* = 8.1 Hz, 2H, Ar-H), 7.38 (s, 4H, Ar-H), 7.31 (d, *J* = 8.7 Hz, 2H, Ar-H), 7.13 (d, *J* = 7.5 Hz, 3H, Ar-H), 4.79 (s, 2H, -CH_2-), 2.34 (s, 3H, -CH_3). ^{13}C NMR (126 MHz, DMSO-d_6) δ 149.61, 148.74, 143.97, 137.93, 137.15, 136.98, 130.36, 129.17, 128.45, 127.26, 127.04, 126.28, 121.51, 120.46, 117.98, 49.91, 21.52. HRMS (ESI): calcd for $C_{21}H_{18}ClN_2O_4S$([M+H]$^+$),429.06703; found,429.06570.

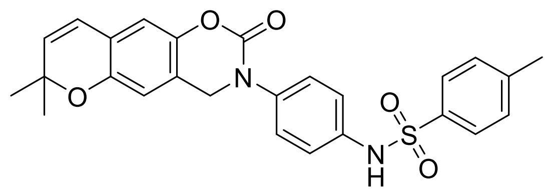

***N*-(4-(7,7-dimethyl-2-oxo-4,7-dihydrochromeno[7,6-*e*][1,3]oxazin-3(2*H*)-yl)phenyl)-4-methylbenzenesulfonamide(L5),** Yield: 40.78%; white solid; m. p. 215.3-217.9 °C; ^{1}H NMR (500 MHz, DMSO-d_6) δ 10.34 (s, 1H, -SO_2NH-), 7.65 (d, *J* = 8.2 Hz, 2H, Ar-H), 7.32 (d, *J* = 8.5 Hz, 2H, Ar-H), 7.27 (d, *J* = 8.9 Hz, 2H, Ar-H), 7.08 (d, *J* = 6.0 Hz, 2H, Ar-H), 6.93 (d, *J* = 8.4 Hz, 1H, Ar-H), 6.59 – 6.49 (m, 2H, Ar-H), 5.81 (d, *J* = 10.0 Hz, 1H, Ar-H), 4.64 (s, 2H, -CH_2-), 2.30 (s, 3H, -CH_3), 1.34 (s, 6H, -CH_3). ^{13}C NMR (126 MHz, DMSO-d_6) δ 152.82, 149.73, 145.03, 143.96, 138.19, 137.17, 132.32, 130.36, 127.26, 126.88, 126.02, 120.43, 115.19, 112.45, 111.37, 109.22, 76.74, 49.93, 27.81, 21.51. HRMS (ESI): calcd for $C_{26}H_{25}N_2O_5S$([M+H]$^+$),477.14787; found,477.14658.

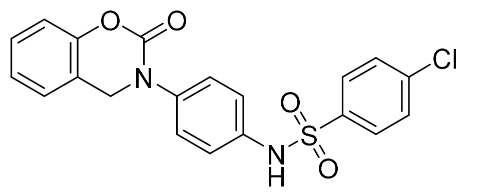

4-chloro-*N*-(4-(2-oxo-2*H*-benzo[*e*][1,3]oxazin-3(4*H*)-yl)phenyl)benzenesulfonamide(L6), Yield: 45.44%; yellow solid; m. p. 195.7-197.5 °C; ^{1}H NMR (600 MHz, DMSO-d_6) δ 10.52 (s, 1H, -SO_2NH-), 7.75 (d, *J* = 6.7 Hz, 2H, Ar-H), 7.62 (d, *J* = 8.7 Hz, 2H, Ar-H), 7.31 (d, *J* = 8.9 Hz, 2H, Ar-H), 7.29 (d, *J* = 7.4 Hz, 1H, Ar-H), 7.22 (d, *J* = 7.6 Hz, 1H, Ar-H), 7.14 – 7.11 (m, 1H, Ar-H), 7.09 (d, *J* = 8.8 Hz, 2H, Ar-H), 7.06 (d, *J* = 8.2 Hz, 1H, Ar-H), 4.77 (s, 2H, -CH_2-). ^{13}C NMR (126 MHz, DMSO-d_6) δ 150.05, 149.82, 138.82, 138.61, 138.46, 136.27, 130.12, 129.36, 129.17, 127.00, 126.54, 124.83, 121.02,

119.37, 116.02, 50.10. HRMS (ESI): calcd for $C_{20}H_{16}N_2O_4SCl([M+H]^+)$,415.05138; found,459.04987.

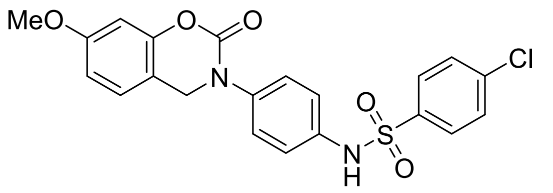

4-chloro-*N*-(4-(6-methoxy-2-oxo-2*H*-benzo[*e*][1,3]oxazin-3(4*H*)-yl)phenyl)benzenesulfonamide(L7), Yield: 54.79%; white solid; m. p. 202.6-204.3 °C; ^{1}H NMR (500 MHz, DMSO-d_6) δ 10.52 (s, 1H, -SO_2NH-), 7.76 (d, J = 6.2 Hz, 2H, Ar-H), 7.62 (d, J = 8.7 Hz, 2H, Ar-H), 7.30 (s, 2H, Ar-H), 7.12 (d, J = 11.8 Hz, 2H, Ar-H), 7.09 (d, J = 3.3 Hz, 1H, Ar-H), 6.71 (d, J = 8.4 Hz, 1H, Ar-H), 6.68 (s, 1H, Ar-H), 4.69 (s, 2H, -CH_2-), 3.72 (s, 3H, -CH_3). ^{13}C NMR (126 MHz, DMSO-d_6) δ 160.18, 150.64, 149.96, 138.79, 138.69, 138.46, 136.25, 130.11, 129.17, 127.17, 126.98, 121.01, 111.16, 101.42, 56.04, 49.77. HRMS (ESI): calcd for $C_{21}H_{18}ClN_2O_5S$ ($[M+H]^+$),445.06195; found,445.06073.

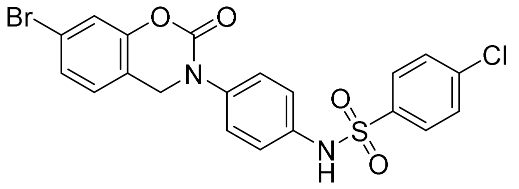

***N*-(4-(7-bromo-2-oxo-2*H*-benzo[*e*][1,3]oxazin-3(4*H*)-yl)phenyl)-4-chlorobenzenesulfonamide(L8),** Yield: 63.15%; yellow solid; m. p. 186.7-188.3 °C; ^{1}H NMR (500 MHz, DMSO-d_6) δ 10.42 (s, 1H, -SO_2NH-), 7.75 (d, J = 8.6 Hz, 2H, Ar-H), 7.62 (d, J = 8.5 Hz, 2H, Ar-H), 7.36 (s, 1H, Ar-H), 7.34 (d, J = 7.9 Hz, 1H, Ar-H), 7.30 (d, J = 8.9 Hz, 2H, Ar-H), 7.19 (d, J = 8.1 Hz, 1H, Ar-H), 7.09 (d, J = 8.9 Hz, 2H, Ar-H), 4.78 (s, 2H, -CH_2-). ^{13}C NMR (151 MHz, DMSO-d_6) δ 150.61, 149.44, 138.79, 138.47, 138.39, 136.39, 130.11, 129.16, 128.36, 127.65, 127.03, 121.13, 121.00, 118.97, 118.90, 49.85. HRMS (ESI): calcd for $C_{20}H_{15}BrClN_2O_4S$ ($[M+H]^+$),492.96189; found,492.96831.

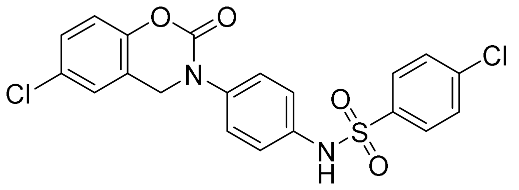

4-chloro-*N*-(4-(6-chloro-2-oxo-2*H*-benzo[*e*][1,3]oxazin-3(4*H*)-yl)phenyl)benzenesulfonamide(L9), Yield: 72.81%; white solid; m. p. 213.3-215.9 °C; ^{1}H NMR (500 MHz, DMSO-d_6) δ 10.51 (s, 1H, -SO_2NH-), 7.90 (t, J = 1.9 Hz, 1H, Ar-H), 7.81 (d, J = 10.1 Hz, 1H, Ar-H), 7.74 (d, J = 6.9 Hz, 1H, Ar-H), 7.50 (t, J = 8.0 Hz, 1H, Ar-H), 7.34 (d, J = 4.0 Hz, 2H, Ar-H), 7.33 (s, 1H, Ar-H), 7.31 (s, 1H, Ar-H), 7.13 – 7.09 (m, 3H, Ar-H), 4.77 (s, 2H, -CH_2-). 13C NMR (126 MHz, DMSO-d6) δ 149.61, 148.72, 138.79, 138.48, 138.36, 136.46, 130.12, 129.18, 128.47, 127.11, 126.29, 121.50, 121.01, 117.98,

49.84. HRMS (ESI): calcd for $C_{20}H_{15}C_{12}N_2O_4S$([M+H]+), 449.01241; found, 449.01147.

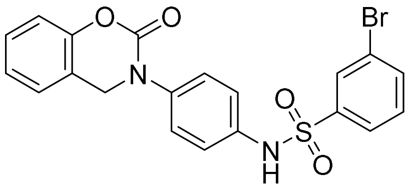

3-bromo-*N*-(4-(2-oxo-2*H*-benzo[*e*][1,3]oxazin-3(4*H*)-yl)phenyl)benzenesulfonamide(L10), Yield: 56.24%; white solid; m. p. 172.1-173.8 °C; ^{1}H NMR (600 MHz, DMSO-d_6) δ 10.53 (s, 1H, -SO_2NH-), 7.89 (t, *J* = 1.8 Hz, 1H, Ar-H), 7.82 (d, *J* = 8.0 Hz, 1H, Ar-H), 7.74 (d, *J* = 7.9 Hz, 1H, Ar-H), 7.50 (t, *J* = 8.0 Hz, 1H, Ar-H), 7.32 (d, *J* = 8.8 Hz, 2H, Ar-H), 7.29 (d, *J* = 7.3 Hz, 1H, Ar-H), 7.22 (d, *J* = 7.6 Hz, 1H, Ar-H), 7.13 (d, *J* = 7.5 Hz, 1H, Ar-H), 7.10 (d, *J* = 8.9 Hz, 2H, Ar-H), 7.06 (d, *J* = 9.4 Hz, 1H, Ar-H), 4.72 (s, 2H, -CH_2-). ^{13}C NMR (126 MHz, DMSO-d_6) δ 150.05, 149.82, 141.96, 138.74, 136.49, 136.14, 132.22, 129.53, 129.37, 127.04, 126.55, 126.28, 124.83, 122.77, 121.17, 119.37, 116.02, 50.10. HRMS (ESI): calcd for $C_{20}H_{16}N_2O_4SBr([M+H]^+)$, 459.00087; found,459.99954.

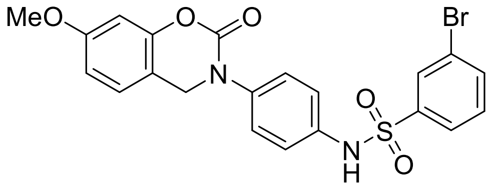

3-bromo-*N*-(4-(6-methoxy-2-oxo-2*H*-benzo[*e*][1,3]oxazin-3(4*H*)-yl)phenyl)benzenesulfonamide(L11), Yield: 44.34%; yellow solid; m. p. 127.4-129.1 °C;^{1}H NMR (500 MHz, DMSO-d_6) δ 10.55 (s, 1H, -SO_2NH-), 7.90 (s, 1H, Ar-H), 7.81 (d, *J* = 13.9 Hz, 1H, Ar-H), 7.74 (d, *J* = 7.8 Hz, 1H, Ar-H), 7.50 (t, *J* = 8.0 Hz, 1H, Ar-H), 7.32 (d, *J* = 8.9 Hz, 2H, Ar-H), 7.13 (d, *J* = 4.0 Hz, 1H, Ar-H), 7.11 (d, *J* = 8.7 Hz, 2H, Ar-H), 6.71 (d, *J* = 8.4 Hz, 1H, Ar-H), 6.68 (s, 1H, Ar-H), 4.69 (s, 2H, -CH_2-), 3.71 (s, 3H, -CH_3). ^{13}C NMR (126 MHz, DMSO-d_6) δ 160.18, 150.63, 149.96, 141.90, 138.84, 136.51, 136.09, 132.21, 129.54, 127.17, 127.03, 126.28, 122.78, 121.15, 111.16, 111.02, 101.42, 56.05, 49.76. HRMS (ESI): calcd for $C_{21}H_{18}BrN_2O_5S$ ([M+H]$^+$),489.01143; found,456.08499.

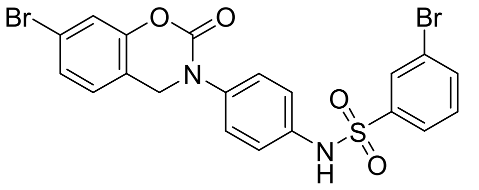

3-bromo-*N*-(4-(7-bromo-2-oxo-2*H*-benzo[*e*][1,3]oxazin-3(4*H*)-yl)phenyl)benzenesulfonamide(L12), Yield: 53.45%; yellow solid; m. p. 161.9-163.2 °C; ^{1}H NMR (500 MHz, DMSO-d_6) δ 10.57 (s, 1H, -SO_2NH-), 7.91 (s, 1H, Ar-H), 7.81 (d, *J* = 7.8 Hz, 1H, Ar-H), 7.74 (d, *J* = 7.8 Hz, 1H, Ar-H), 7.50 (t, *J* = 7.9 Hz, 1H, Ar-H), 7.35 (d, *J* = 12.5 Hz, 2H, Ar-H),

7.32 (d, J = 2.2 Hz, 2H, Ar-H), 7.19 (d, J = 8.1 Hz, 1H, Ar-H), 7.12 (d, J = 8.9 Hz, 2H, Ar-H), 4.74 (s, 2H, $-CH_2-$). ^{13}C NMR (126 MHz, DMSO-d_6) δ 150.59, 149.45, 141.89, 138.52, 136.51, 136.25, 132.21, 129.54, 128.36, 127.65, 127.07, 126.28, 122.78, 121.15, 121.12, 49.84. HRMS (ESI): calcd for $C_{20}H_{15}Br_2N_2O_4S$([M+H]+), 536.91138; found, 536.91046.

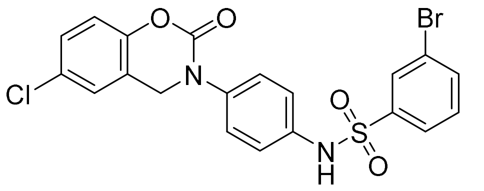

3-bromo-*N*-(4-(6-chloro-2-oxo-2*H*-benzo[*e*][1,3]oxazin-3(4*H*)-yl)phenyl)benzenesulfonamide(L13), Yield: 46.21%; yellow solid; m. p. 219.6-221.9 °C; ^1H NMR (500 MHz, DMSO-d_6) δ 10.56 (s, 1H, $-SO_2NH-$), 7.91 (s, 1H, Ar-H), 7.82 (d, J = 8.0 Hz, 1H, Ar-H), 7.75 (d, J = 7.6 Hz, 1H, Ar-H), 7.50 (t, J = 8.0 Hz, 1H, Ar-H), 7.33 (s, 4H, Ar-H), 7.12 (s, 3H, Ar-H), 4.77 (s, 2H, $-CH_2-$).^{13}C NMR (126 MHz, DMSO-d_6) δ 149.60, 148.71, 141.89, 138.51, 136.52, 136.29, 132.22, 129.55, 129.17, 128.48, 127.15, 126.28, 122.79, 121.48, 121.15, 117.97, 49.84. HRMS (ESI): calcd for $C_{20}H_{15}BrClN_2O_4S$ ($[M+H]^+$),492.96189; found,492.96085.

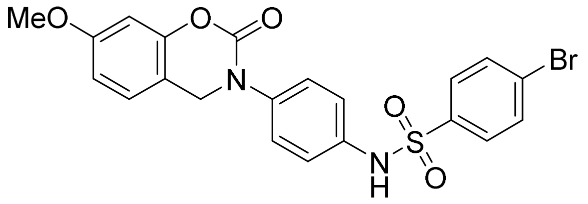

4-bromo-*N*-(4-(6-methoxy-2-oxo-2*H*-benzo[*e*][1,3]oxazin-3(4*H*)-yl)phenyl)benzenesulfonamide(L14), Yield: 47.87%; yellow solid; m. p. 175.9-177.1 °C; ^1H NMR (500 MHz, DMSO-d_6) δ 10.46 (s, 1H, $-SO_2NH-$), 7.76 (d, J = 8.6 Hz, 2H, Ar-H), 7.68 (d, J = 8.7 Hz, 2H, Ar-H), 7.31 (d, J = 8.8 Hz, 2H, Ar-H), 7.11 (d, J = 14.9 Hz, 2H, Ar-H), 7.08 (s, 1H, Ar-H), 6.71 (d, J = 8.4 Hz, 1H, Ar-H), 6.68 (s, 1H, Ar-H), 4.69 (s, 2H, $-CH_2-$), 3.72 (s, 3H, $-CH_3$). ^{13}C NMR (126 MHz, DMSO-d_6) δ 160.18, 150.64, 149.96, 139.21, 138.69, 136.22, 133.05, 129.24, 127.51, 127.17, 126.99, 121.00, 111.16, 111.03, 101.42, 56.05, 49.77. HRMS (ESI): calcd for $C_{21}H_{18}BrN_2O_5S$([M+H]+), 489.01143; found, 489.01019.

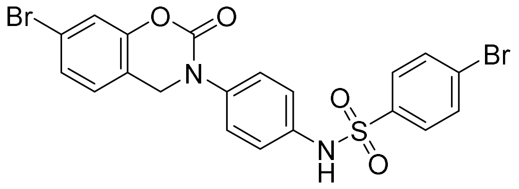

4-bromo-*N*-(4-(7-bromo-2-oxo-2*H*-benzo[*e*][1,3]oxazin-3(4*H*)-yl)phenyl)benzenesulfonamide(L15), Yield: 62.43%; yellow solid; m. p. 206.2-208.9 °C; ^1H NMR (500 MHz, DMSO-d_6) δ 10.54 (s, 1H, $-SO_2NH-$), 7.76 (d, J = 8.7 Hz, 2H, Ar-

H), 7.67 (d, J = 8.7 Hz, 2H, Ar-H), 7.36 (d, J = 1.9 Hz, 1H, Ar-H), 7.32 (d, J = 1.9 Hz, 1H, Ar-H), 7.30 (d, J = 8.9 Hz, 2H, Ar-H), 7.19 (d, J = 8.1 Hz, 1H, Ar-H), 7.09 (d, J = 8.8 Hz, 2H, Ar-H), 4.73 (s, 2H, $-CH_2-$). ^{13}C NMR (151 MHz, DMSO-d_6) δ 150.61, 149.44, 139.21, 138.39, 136.38, 133.04, 129.22, 128.36, 127.65, 127.50, 127.03, 121.13, 121.00, 118.96, 118.90, 49.85. HRMS (ESI): calcd for $C_{20}H_{15}Br_2N_2O_4S$ ([M+H]$^+$), 536.91138; found,536.90802.

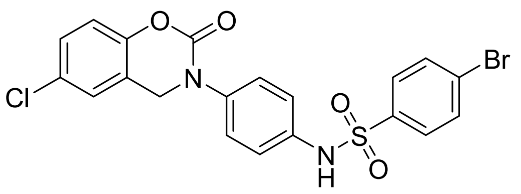

4-bromo-*N*-(4-(7-chloro-2-oxo-2*H*-benzo[*e*][1,3]oxazin-3(4*H*)-yl)phenyl)benzenesulfonamide(L16), Yield: 53.45%; yellow solid; m. p. 221.4-224.1 °C; ^1H NMR (600 MHz, DMSO-d_6) δ 10.60 (s, 1H, $-SO_2NH-$), 7.76 (d, J = 8.7 Hz, 2H, Ar-H), 7.68 (d, J = 8.6 Hz, 2H, Ar-H), 7.35 (d, J = 6.3 Hz, 2H, Ar-H), 7.30 (d, J = 8.9 Hz, 2H, Ar-H), 7.12 (s, 1H, Ar-H), 7.10 (s, 1H, Ar-H), 7.09 (s, 1H, Ar-H), 4.76 (s, 2H, $-CH_2-$). ^{13}C NMR (151 MHz, DMSO-d_6) δ 149.60, 148.71, 139.20, 138.36, 136.43, 133.05, 129.23, 128.46, 127.51, 127.13, 126.31, 126.25, 121.51, 121.00, 118.00, 49.84. HRMS (ESI): calcd for $C_{20}H_{15}BrClN_2O_4S$ ([M+H]$^+$),492.96189; found,492.96051.

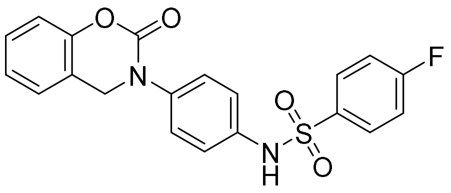

4-fluoro-*N*-(4-(2-oxo-2*H*-benzo[*e*][1,3]oxazin-3(4*H*)-yl)phenyl)benzenesulfonamide(L17), Yield: 57.95%; brown solid; m. p. 203.4-205.1 °C; ^1H NMR (600 MHz, DMSO-d_6) δ 10.47 (s, 1H, $-SO_2NH-$), 7.82 (dd, J = 8.9, 5.1 Hz, 2H, Ar-H), 7.38 (t, J = 8.8 Hz, 2H, Ar-H), 7.31 (d, J = 8.9 Hz, 2H, Ar-H), 7.29 (d, J = 7.4 Hz, 1H, Ar-H), 7.22 (dd, J = 7.6, 1.5 Hz, 1H, Ar-H), 7.13 (d, J = 8.6 Hz, 1H, Ar-H), 7.10 (d, J = 8.8 Hz, 2H, Ar-H), 7.06 (d, J = 8.2 Hz, 1H, Ar-H), 4.77 (s, 2H, $-CH_2-$). ^{13}C NMR (151 MHz, DMSO-d_6) δ 165.73, 150.05, 149.81, 138.51, 136.41, 136.32, 130.28, 126.94, 126.65, 124.92, 120.98, 120.75, 119.36, 117.17, 115.91, 50.11. ^{19}F NMR (471 MHz, DMSO-d_6) δ -105.04. HRMS (ESI): calcd for $C_{20}H_{16}FN_2O_4S$([M+H]$^+$),399.08093; found,399.07983.

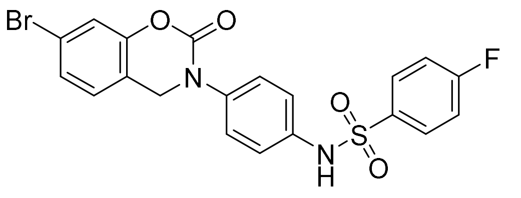

***N*-(4-(7-bromo-2-oxo-2*H*-benzo[*e*][1,3]oxazin-3(4*H*)-yl)phenyl)-4-fluorobenzenesulfonamide(L18),** Yield: 43.77%;

brown solid; m. p. 198.8-200.3 °C; ^{1}H NMR (500 MHz, DMSO-d_6) δ 10.56 – 10.42 (s, 1H, -SO$_2$NH-), 7.82 (dd, *J* = 8.0, 4.2 Hz, 2H, Ar-H), 7.42 – 7.35 (m, 3H, Ar-H), 7.34 (d, *J* = 8.1 Hz, 1H, Ar-H), 7.30 (d, *J* = 8.8 Hz, 2H, Ar-H), 7.19 (d, *J* = 9.3 Hz, 1H, Ar-H), 7.10 (dd, *J* = 8.9, 2.6 Hz, 2H, Ar-H), 4.74 (s, 2H, -CH$_2$-). ^{13}C NMR (126 MHz, DMSO-d6) δ 163.90, 150.60, 149.46, 138.29, 136.55, 136.30, 130.34, 130.26, 128.37, 127.65, 127.03, 120.87, 118.97, 117.27, 117.09, 49.87. ^{19}F NMR (471 MHz, DMSO-d_6) δ -105.59. HRMS (ESI): calcd for $C_{20}H_{15}BrFN_2O_4S$ ([M+H]$^+$),476.99144; found,476.99011.

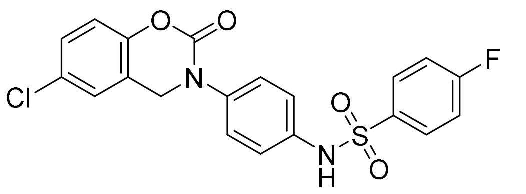

***N*-(4-(6-chloro-2-oxo-2*H*-benzo[*e*][1,3]oxazin-3(4*H*)-yl)phenyl)-4-fluorobenzenesulfonamide(L19),** Yield: 64.33%; white solid; m. p. 181.5-183.7 °C; ^{1}H NMR (500 MHz, DMSO-d_6) δ 10.46 (s, 1H, -SO$_2$NH-), 7.86 – 7.80 (m, 2H, Ar-H), 7.39 (t, *J* = 8.8 Hz, 2H, Ar-H), 7.35 (s, 2H, Ar-H), 7.30 (d, *J* = 8.4 Hz, 2H, Ar-H), 7.11 (d, *J* = 8.6 Hz, 3H, Ar-H), 4.76 (s, 2H, -CH$_2$-). ^{13}C NMR (126 MHz, DMSO-d_6) δ 165.91, 149.61, 148.72, 138.27, 136.60, 130.27, 129.17, 128.46, 127.10, 126.28, 122.37, 121.49, 120.88, 117.97, 117.26, 49.86. ^{19}F NMR (471 MHz, DMSO-d_6) δ -105.92, -105.60. HRMS (ESI): calcd for $C_{20}H_{15}FClN_2O_4S$ ([M+H]$^+$),433.04196; found,433.04068.

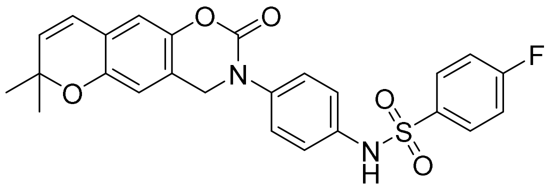

***N*-(4-(7,7-dimethyl-2-oxo-4,7-dihydrochromeno[7,6-*e*][1,3]oxazin-3(2*H*)-yl)phenyl)-4-fluorobenzenesulfonamide(L20),** Yield: 44.19%; white solid; m. p. 189.2-191.4 °C; ^{1}H NMR (500 MHz, DMSO-d_6) δ 10.51 (s, 1H, -SO$_2$NH-), 7.86 (dt, *J* = 7.2, 3.0 Hz, 2H, Ar-H), 7.42 (t, *J* = 8.4 Hz, 2H, Ar-H), 7.33 (d, *J* = 8.3 Hz, 2H, Ar-H), 7.13 (d, *J* = 8.6 Hz, 2H, Ar-H), 6.98 (d, *J* = 8.3 Hz, 1H, Ar-H), 6.58 (t, *J* = 8.8 Hz, 2H, Ar-H), 5.85 (d, *J* = 10.0 Hz, 1H, Ar-H), 4.69 (s, 2H, -CH$_2$-), 1.38 (s, 6H, -CH$_3$). ^{13}C NMR (151 MHz, Ethanol-d_6) δ 164.33, 162.67, 151.42, 148.32, 143.62, 137.13, 134.95, 130.92, 128.91, 125.52, 124.61, 119.48, 115.84, 115.68, 113.78, 111.05, 109.97, 107.81, 75.33, 48.48, 26.40. ^{19}F NMR (471 MHz, DMSO-d_6) δ -105.61. HRMS (ESI): calcd for $C_{25}H_{22}FN_2O_5S$([M+H]+), 481.12280; found, 481.12149.

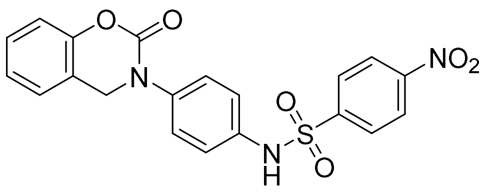

4-nitro-*N*-(4-(2-oxo-2*H*-benzo[*e*][1,3]oxazin-3(4*H*)-yl)phenyl)benzenesulfonamide(L21), Yield: 58.75%; yellow solid; m. p. 254.3-256.1 °C; ^{1}H NMR (500 MHz, DMSO-d_6) δ 10.77 (s, 1H, -SO_2NH-), 8.36 (d, J = 8.9 Hz, 2H, Ar-H), 8.01 (d, J = 8.8 Hz, 2H, Ar-H), 7.33 (d, J = 8.8 Hz, 2H, Ar-H), 7.28 (d, J = 8.1 Hz, 1H, Ar-H), 7.22 (d, J = 9.3 Hz, 1H, Ar-H), 7.15 – 7.10 (m, 3H, Ar-H), 7.06 (d, J = 8.2 Hz, 1H, Ar-H), 4.77 (s, 2H, -CH_2-). ^{13}C NMR (126 MHz, DMSO-d_6) δ 150.46, 150.05, 149.79, 145.38, 138.92, 135.81, 129.37, 128.84, 127.12, 126.54, 125.35, 124.84, 121.32, 119.34, 116.03, 50.09. HRMS (ESI): calcd for $C_{20}H_{16}N_3O_6S$([M+H]$^+$),426.07543; found,426.07422.

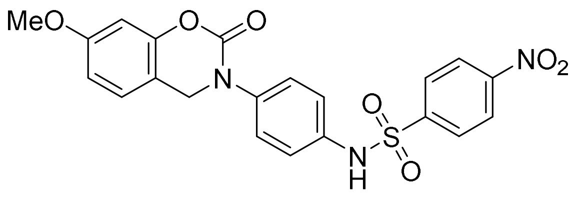

N-(4-(6-methoxy-2-oxo-2H-benzo[e][1,3]oxazin-3(4H)-yl)phenyl)-4-nitrobenzenesulfonamide(L22), Yield: 47.25%; yellow solid; m. p. 223.9-225.4 °C; ^{1}H NMR (600 MHz, DMSO-d_6) δ 10.77 (s, 1H, -SO2NH-), 8.36 (d, J = 8.8 Hz, 2H, Ar-H), 8.00 (d, J = 8.8 Hz, 2H, Ar-H), 7.31 (d, J = 8.9 Hz, 2H, Ar-H), 7.11 (t, J = 8.0 Hz, 3H, Ar-H), 6.71 (s, 1H, Ar-H), 6.67 (s, 1H, Ar-H), 4.69 (s, 2H, -CH_2-), 3.71 (s, 3H, -CH_3). 13C NMR (151 MHz, DMSO-d6) δ 160.19, 150.61, 150.44, 149.95, 145.39, 138.97, 135.83, 128.83, 127.17, 127.09, 125.35, 121.31, 111.17, 111.01, 101.42, 56.05, 49.76. HRMS (ESI): calcd for C21 H18 N3 O7 S ([M+H]+), 456.08600; found, 456.08499.

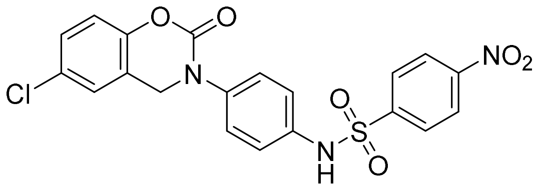

***N*-(4-(6-chloro-2-oxo-2*H*-benzo[*e*][1,3]oxazin-3(4*H*)-yl)phenyl)-4-nitrobenzenesulfonamide(L23),** Yield: 41.77%; yellow solid; m. p. 208.9-211.2 °C; ^{1}H NMR (500 MHz, DMSO-d_6) δ 10.51 (s, 1H, -SO_2NH-), 8.36 (d, J = 8.9 Hz, 2H, Ar-H), 8.01 (d, J = 8.7 Hz, 2H, Ar-H), 7.36 (s, 1H, Ar-H), 7.34 (s, 1H, Ar-H), 7.31 (d, J = 9.0 Hz, 2H, Ar-H), 7.12 (dt, J = 6.7, 1.5 Hz, 2H, Ar-H), 7.00 (s, 1H, Ar-H), 4.76 (s, 2H, -CH_2-).^{13}C NMR (126 MHz, DMSO-d_6) δ 150.43, 149.60, 148.70, 145.47, 138.58, 136.17, 129.18, 128.83, 128.46, 127.22, 126.29, 125.34, 122.16, 121.30, 117.99, 49.84. HRMS (ESI): calcd for $C_{20}H_{15}ClN_3O_6S$ ([M+H]$^+$),460.03646; found,460.03506.

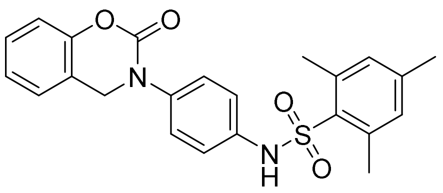

2,4,6-trimethyl-*N*-(4-(2-oxo-2*H*-benzo[*e*][1,3]oxazin-3(4*H*)-yl)phenyl)benzenesulfonamide(L24), Yield: 42.67%; white solid; m. p. 205.9-207.5 ℃; ^{1}H NMR (500 MHz, DMSO-d_6) δ 10.32 (s, 1H, -SO_2NH-), 7.30 (d, *J* = 7.3 Hz, 1H, Ar-H), 7.27 (d, *J* = 8.8 Hz, 2H, Ar-H), 7.21 (d, *J* = 7.7 Hz, 1H, Ar-H), 7.12 (t, *J* = 8.1 Hz, 1H, Ar-H), 7.05 (d, *J* = 9.3 Hz, 1H, Ar-H), 6.98 (d, *J* = 3.8 Hz, 3H, Ar-H), 6.96 (s, 1H, Ar-H), 4.75 (s, 2H, -CH_2-), 2.54 (s, 6H, -CH_3), 2.19 (s, 3H, -CH_3). ^{13}C NMR (126 MHz, DMSO-d_6) δ 150.06, 149.82, 142.78, 139.19, 137.70, 136.78, 134.27, 132.47, 129.35, 127.02, 126.53, 124.81, 122.15, 119.35, 116.02, 50.19, 23.00, 20.93. HRMS (ESI): calcd for $C_{23}H_{23}N_2O_4S$([M+H]+),423.13730; found,423.13586.

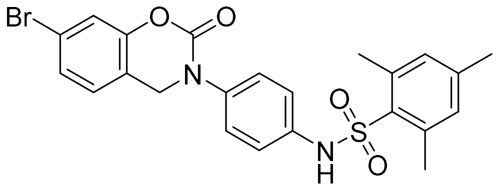

***N*-(4-(7-bromo-2-oxo-2*H*-benzo[*e*][1,3]oxazin-3(4*H*)-yl)phenyl)-2,4,6-trimethylbenzenesulfonamide(L25),** Yield: 74.32%; white solid; m. p. 206.2-208.9 ℃; ^{1}H NMR (500 MHz, DMSO-d_6) δ 10.38 (s, 1H, -SO_2NH-), 7.36 (s, 1H, Ar-H), 7.33 (d, *J* = 6.1 Hz, 1H, Ar-H), 7.27 (d, *J* = 8.9 Hz, 2H, Ar-H), 7.18 (d, *J* = 8.1 Hz, 1H, Ar-H), 6.99 (s, 2H, Ar-H), 6.98 – 6.96 (m, 2H, Ar-H), 4.71 (s, 2H, -CH_2-), 2.54 (s, 6H, -CH_3), 2.19 (s, 3H, -CH_3). ^{13}C NMR (126 MHz, DMSO-d_6) δ 150.61, 149.46, 142.79, 139.19, 137.46, 136.92, 134.23, 132.47, 128.36, 127.64, 127.05, 121.13, 119.29, 118.96, 118.88, 49.94, 23.01, 20.93. HRMS (ESI): calcd for $C_{23}H_{22}BrN_2O_4S$ ([M+H]$^+$),501.04782; found,501.02639.

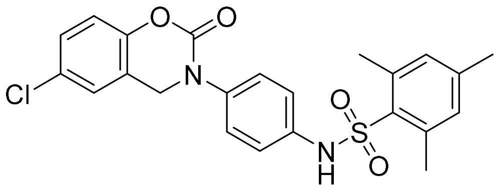

***N*-(4-(6-chloro-2-oxo-2*H*-benzo[*e*][1,3]oxazin-3(4*H*)-yl)phenyl)-2,4,6-trimethylbenzenesulfonamide(L26),** Yield: 49.79%; white solid; m. p. 229.1-231.4 ℃; ^{1}H NMR (600 MHz, DMSO-d_6) δ 10.37 (s, 1H, -SO_2NH-), 7.35 (d, *J* = 9.0 Hz, 2H, Ar-H), 7.26 (d, *J* = 8.9 Hz, 2H, Ar-H), 7.10 (s, 1H, Ar-H), 6.99 (s, 2H, Ar-H), 6.98 (s, 1H, Ar-H), 6.96 (s, 1H, Ar-H), 4.74 (s, 2H, -CH_2-), 2.54 (s, 6H, -CH_3), 2.19 (s, 3H, -CH_3). ^{13}C NMR (126 MHz, DMSO-d6) δ 149.62, 148.73, 142.79, 139.19, 137.44, 132.47, 129.17, 128.44, 127.12, 126.28, 121.51, 119.31, 117.99, 49.92, 23.00, 20.93. HRMS (ESI): calcd

for C23 H22 Cl N2 O4 S ([M+H]+), 457.09833; found, 457.09717.

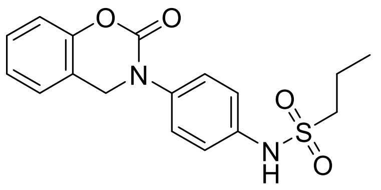

***N*-(4-(2-oxo-2*H*-benzo[*e*][1,3]oxazin-3(4*H*)-yl)phenyl)propane-1-sulfonamide(L27),** Yield: 58.19%; white solid; m. p. 129.9-131.5 °C; ^{1}H NMR (600 MHz, DMSO-d_6) δ 9.89 (s, 1H, -SO_2NH-), 7.38 (d, *J* = 8.9 Hz, 2H, Ar-H), 7.33 – 7.29 (m, 1H, Ar-H), 7.25 (d, *J* = 7.6 Hz, 1H, Ar-H), 7.21 (d, *J* = 8.8 Hz, 2H, Ar-H), 7.14 (t, *J* = 8.1 Hz, 1H, Ar-H), 7.08 (d, *J* = 9.3 Hz, 1H, Ar-H), 4.83 (s, 2H, -CH_2-), 3.07 – 3.03 (m, 2H, -CH_2-), 1.66 (h, *J* = 7.5 Hz, 2H, -CH_2-), 0.91 (t, *J* = 7.5 Hz, 3H, -CH_3). ^{13}C NMR (151 MHz, DMSO-d_6) δ 150.13, 149.88, 138.01, 137.46, 129.37, 127.19, 126.57, 124.82, 120.23, 119.40, 116.05, 52.96, 50.31, 17.39, 13.08. HRMS (ESI): calcd for $C_{20}H_{19}N_2O_4S$([M+H]$^+$),347.10600; found,347.10471.

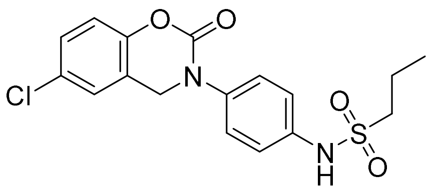

***N*-(4-(6-chloro-2-oxo-2*H*-benzo[*e*][1,3]oxazin-3(4*H*)-yl)phenyl)propane-1-sulfonamide(L28),** Yield: 52.74%; white solid; m. p. 169.6-171.4 °C; ^{1}H NMR (500 MHz, DMSO-d_6) δ 9.92 (s, 1H, -SO_2NH-), 7.38 (s, 2H, Ar-H), 7.37 (s, 2H, Ar-H), 7.22 (s, 1H, Ar-H), 7.21 (s, 1H, Ar-H), 7.13 (d, *J* = 8.3 Hz, 1H, Ar-H), 4.81 (s, 2H, -CH_2-), 3.08 – 3.03 (m, 2H, -CH_2-), 1.66 (h, *J* = 7.6 Hz, 2H, -CH_2-), 0.91 (t, *J* = 7.5 Hz, 3H, -CH_3). ^{13}C NMR (126 MHz, DMSO-d_6) δ 149.68, 148.78, 137.75, 137.65, 129.19, 128.47, 127.30, 126.32, 121.52, 120.21, 118.00, 52.96, 50.06, 17.41, 13.08. HRMS (ESI): calcd for $C_{17}H_{18}ClN_2O_4S$ ([M+H]$^+$),381.06703; found,381.06558.

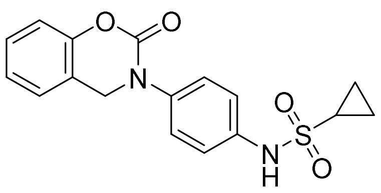

***N*-(4-(2-oxo-2*H*-benzo[*e*][1,3]oxazin-3(4*H*)-yl)phenyl)cyclopropanesulfonamide(L29),** Yield: 75.33%; white solid; m. p. 139.7-141.6 °C; H NMR (500 MHz, DMSO-d_6) δ 9.84 (s, 1H, -SO_2NH-), 7.39 (s, 1H, Ar-H), 7.37 (s, 1H, Ar-H), 7.31 (s, 1H, Ar-H), 7.25 (t, J = 9.1 Hz, 3H, Ar-H), 7.15 (s, 1H, Ar-H), 7.09 (s, 1H, Ar-H), 4.83 (s, 2H, -CH2), 2.61 (t, 1H, -CH), 0.92 (d, 4H, -CH2). ^{13}C NMR (151 MHz, DMSO-d_6) δ 150.11, 149.88, 138.20, 137.41, 129.36, 127.03, 126.56, 124.81, 121.01, 119.39, 116.03, 50.30, 30.12, 5.56. HRMS (ESI): calcd for $C_{17}H_{17}N_2O_4S$([M+H]$^+$),345.09035; found,345.08908.

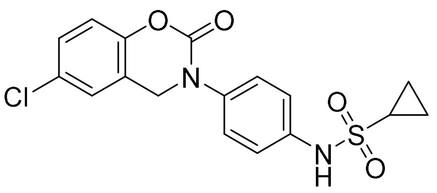

***N*-(4-(6-chloro-2-oxo-2*H*-benzo[*e*][1,3]oxazin-3(4*H*)-yl)phenyl)cyclopropanesulfonamide(L30),** Yield: 68.69%; white solid; m. p. 198.4-200.9 °C; ^{1}H NMR (500 MHz, DMSO-d_6) δ 9.86 (s, 1H, -SO_2NH-), 7.39 (s, 2H, Ar-H), 7.37 (s, 2H, Ar-H), 7.26 (s, 1H, Ar-H), 7.24 (s, 1H, Ar-H), 7.13 (d, J = 8.8 Hz, 1H, Ar-H), 4.82 (s, 2H, -CH_2-), 2.62 (p, J = 6.5 Hz, 1H, -CH-), 0.92 (d, J = 6.4 Hz, 4H, -CH_2-).^{13}C NMR (126 MHz, DMSO-d_6) δ 149.68, 148.79, 137.94, 137.60, 129.19, 128.46, 127.16, 126.34, 121.53, 120.98, 118.01, 50.05, 30.13, 5.58. HRMS (ESI): calcd for $C_{17}H_{16}ClN_2O_4S$ ([M+H]$^+$),379.05138; found,379.05054

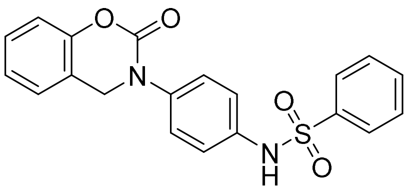

***N*-(4-(2-oxo-2*H*-benzo[*e*][1,3]oxazin-3(4*H*)-yl)phenyl)benzenesulfonamide(L31),** Yield: 42.47%; white solid; m. p. 181.7-182.9 °C; ^{1}H NMR (600 MHz, DMSO-d_6) δ 10.45 (s, 1H, -SO_2NH-), 7.77 (d, J = 7.1 Hz, 2H, Ar-H), 7.59 (t, J = 7.4 Hz, 1H, Ar-H), 7.53 (t, J = 7.6 Hz, 2H, Ar-H), 7.29 (d, J = 8.9 Hz, 3H, Ar-H), 7.22 (d, J = 7.6 Hz, 1H, Ar-H), 7.13 (dd, J = 7.5, 1.1 Hz, 1H, Ar-H), 7.09 (d, J = 8.8 Hz, 2H, Ar-H), 7.06 (d, J = 7.1 Hz, 1H, Ar-H), 4.76 (s, 2H, -CH_2-). ^{13}C NMR (126 MHz, DMSO-d_6) δ 155.53, 147.36, 140.30, 132.98, 129.51, 128.88, 128.09, 127.25, 126.02, 125.69, 125.23, 119.27, 115.36, 112.68, 41.94. HRMS (ESI): calcd for $C_{20}H_{17}N_2O_4SCl$([M+H]$^+$),381.09035; found,381.09035.

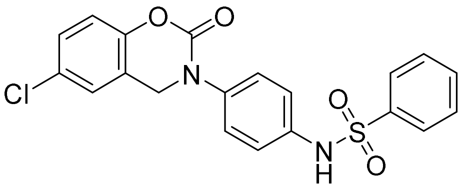

***N*-(4-(6-chloro-2-oxo-2*H*-benzo[*e*][1,3]oxazin-3(4*H*)-yl)phenyl)benzenesulfonamide(L32),** Yield: 64.89%; white solid; m. p. 203.4-204.9 °C; ^{1}H NMR (500 MHz, DMSO-d_6) δ 10.49 (s, 1H, -SO_2NH-), 7.78 (d, J = 5.4 Hz, 2H, Ar-H), 7.58 (d, J = 7.3 Hz, 1H, Ar-H), 7.57 – 7.51 (m, 2H, Ar-H), 7.34 (d, J = 7.2 Hz, 2H, Ar-H), 7.29 (d, J = 8.9 Hz, 2H, Ar-H), 7.14 – 7.08 (m, 3H, Ar-H), 4.75 (s, 2H, -CH_2-). ^{13}C NMR (126 MHz, DMSO-d_6) δ 149.61, 148.72, 139.97, 138.07, 136.82, 133.61, 129.93, 129.16, 128.46, 127.21, 127.06, 126.28, 121.49, 120.65, 117.97, 49.87. HRMS (ESI): calcd for $C_{20}H_{16}ClN_2O_4S$ ([M+H]$^+$),415.05138; found,415.05023.

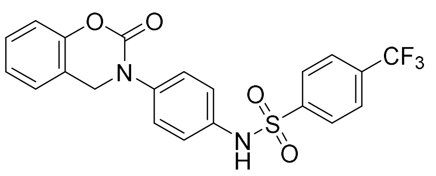

***N*-(4-(2-oxo-2*H*-benzo[*e*][1,3]oxazin-3(4*H*)-yl)phenyl)-4-(trifluoromethyl)benzenesulfonamide(L33),** Yield: 55.14%; white solid; m. p. 182.6-184.9 °C; ^{1}H NMR (600 MHz, DMSO-d_6) δ 10.69 (s, 1H, -SO_2NH-), 7.97 (d, *J* = 10.9 Hz, 2H, Ar-H), 7.95 (d, *J* = 8.7 Hz, 2H, Ar-H), 7.32 (d, *J* = 8.8 Hz, 2H, Ar-H), 7.29 (t, *J* = 7.8 Hz, 1H, Ar-H), 7.22 (d, *J* = 7.5 Hz, 1H, Ar-H), 7.13 (d, *J* = 9.0 Hz, 2H, Ar-H), 7.11 (d, *J* = 2.6 Hz, 1H, Ar-H), 7.06 (d, *J* = 8.2 Hz, 1H, Ar-H), 4.77 (s, 2H, -CH_2-). ^{13}C NMR (151 MHz, DMSO-d_6) δ 150.05, 149.81, 143.93, 138.79, 135.99, 133.28, 129.36, 128.22, 127.26, 127.24, 127.04, 124.82, 121.17, 119.36, 116.01, 50.07. ^{19}F NMR (471 MHz, DMSO-d_6) δ -61.56. HRMS (ESI): calcd for $C_{21}H_{15}F_3N_2O_4S$([M+H]$^+$),449.07774; found,449.07642.

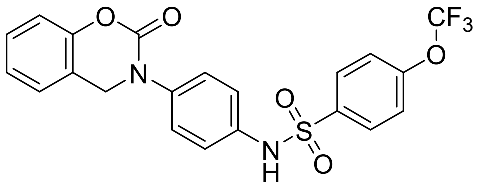

***N*-(4-(2-oxo-2*H*-benzo[*e*][1,3]oxazin-3(4*H*)-yl)phenyl)-4-(trifluoromethoxy)benzenesulfonamide(L34),** Yield: 57.89%; white solid; m. p. 144.5-145.8 °C; ^{1}H NMR (500 MHz, DMSO-d_6) δ 10.58 (s, 1H, -SO_2NH-), 7.94 – 7.84 (m, 2H, Ar-H), 7.60 (m, 2H, Ar-H), 7.34 (m, 2H, Ar-H), 7.30 (m, 1H, Ar-H), 7.22 (dd, *J* = 7.6, 1.6 Hz, 1H, Ar-H), 7.13 (dd, *J* = 7.5, 1.2 Hz, 1H, Ar-H), 7.12 (m, 2H, Ar-H), 7.06 (dd, *J* = 8.2, 1.1 Hz, 1H, Ar-H), 4.85 – 4.67 (s, 2H, -CH2-). ^{13}C NMR (126 MHz, DMSO-d_6) δ 151.65, 150.06, 149.81, 138.94, 138.61, 136.25, 129.87, 129.36, 127.02, 126.54, 124.83, 122.09, 121.37, 120.94, 119.37, 116.02, 50.09. ^{19}F NMR (471 MHz, DMSO-d_6) δ -56.60. HRMS (ESI): calcd for $C_{21}H_{16}F_3N_2O_5S$([M+H]$^+$),465.07265; found,465.07089.

3. ^{1}H NMR, ^{13}C NMR , ^{19}F NMRand HRMS data

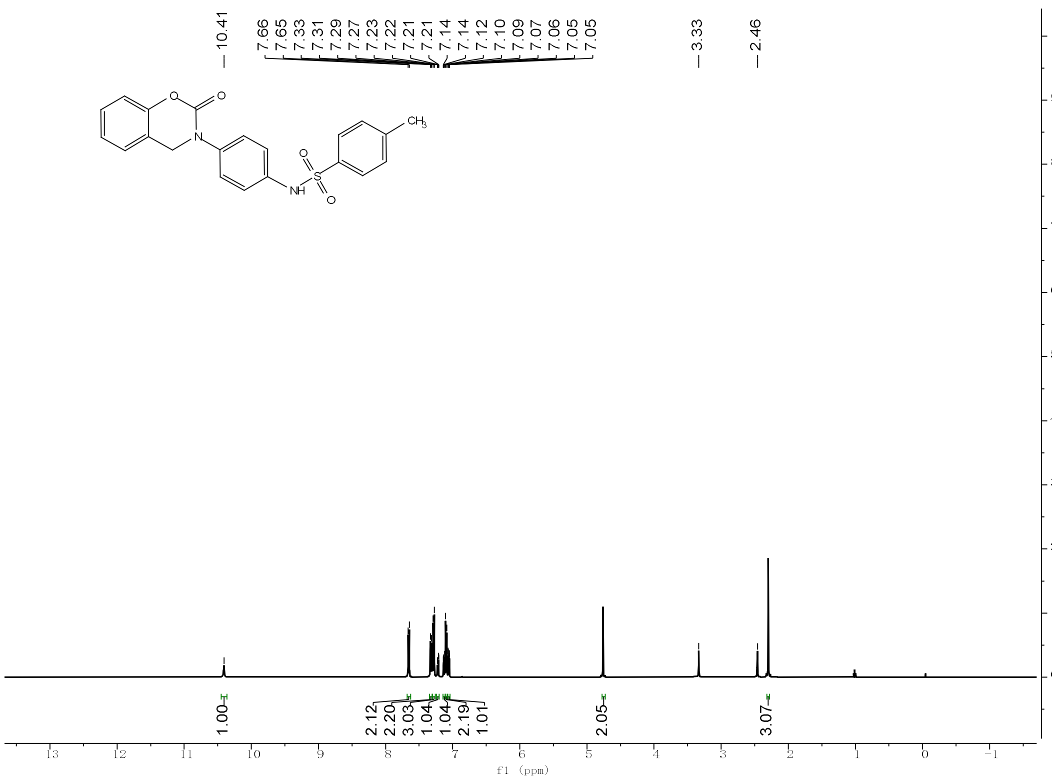

Figure 1. ^{1}H NMR (500 MHz, DMSO-d_6) spectrum of compound L1.

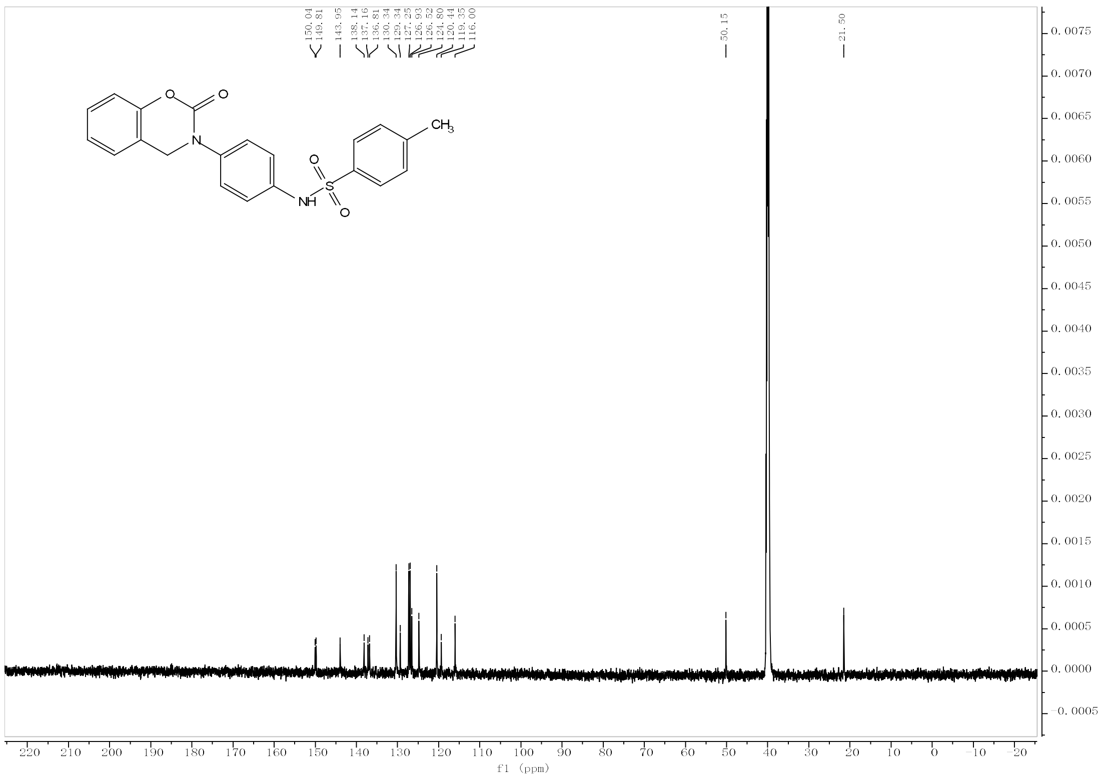

Figure 2. ^{13}C NMR (151 MHz, DMSO-d_6) spectrum of compound L1.

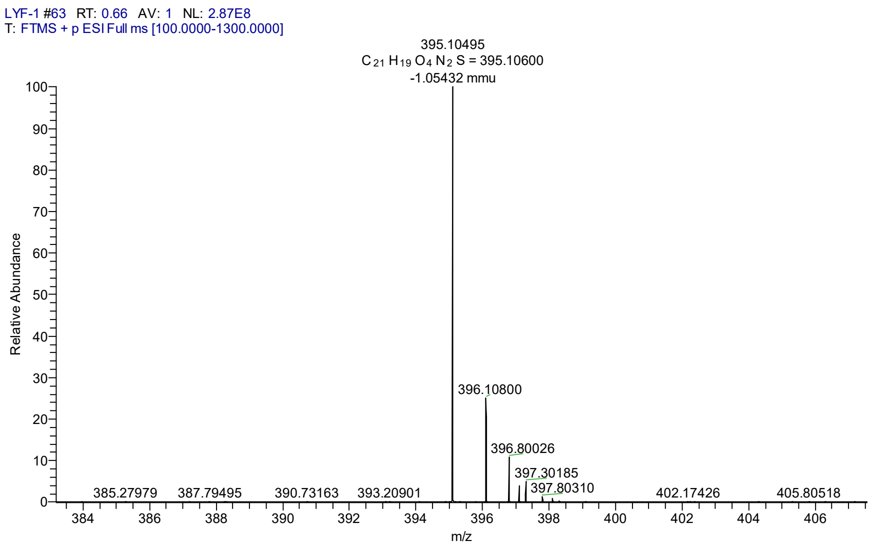

Figure 3. HRMS of compound L1.

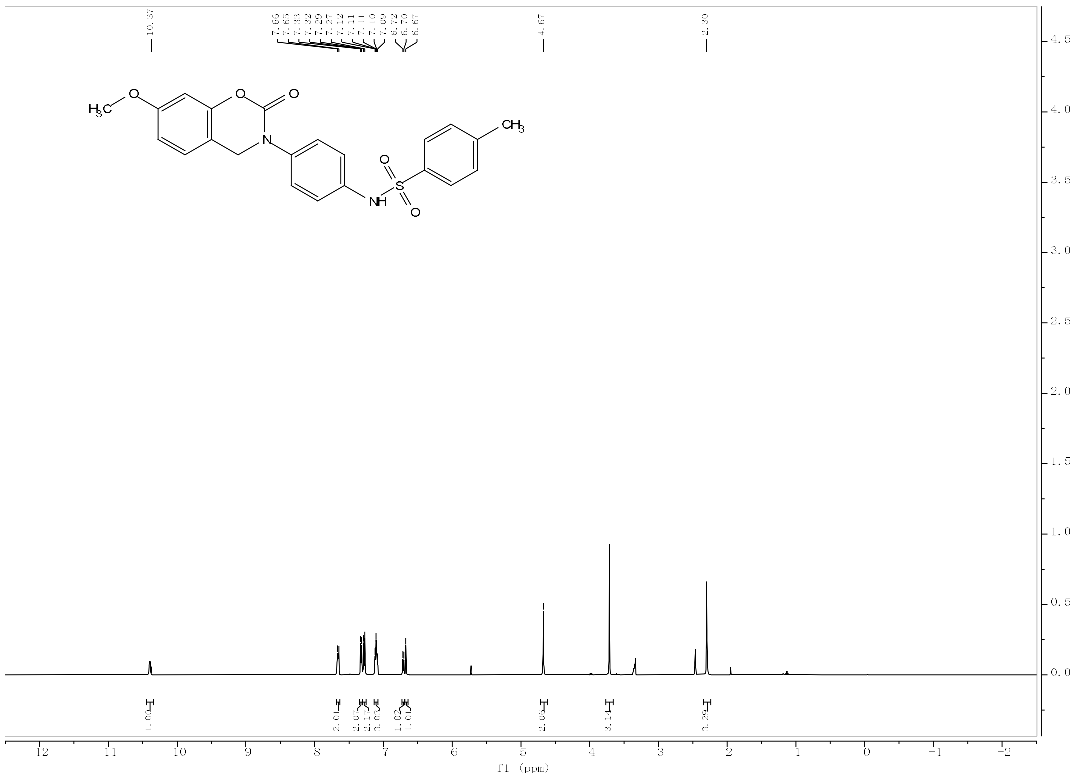

Figure 4. ^{1}H NMR (500 MHz, DMSO-d_6) spectrum of compound L2.

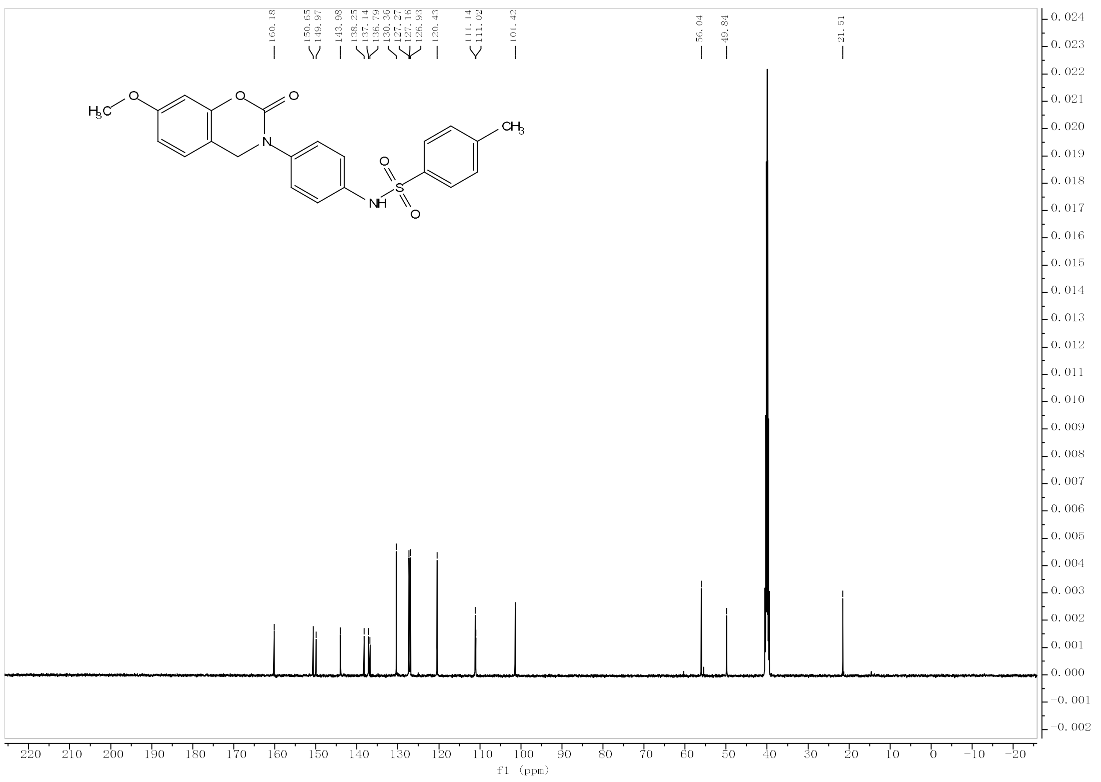

Figure 5. ^{13}C NMR (126 MHz, DMSO-d_6) spectrum of compound L2.

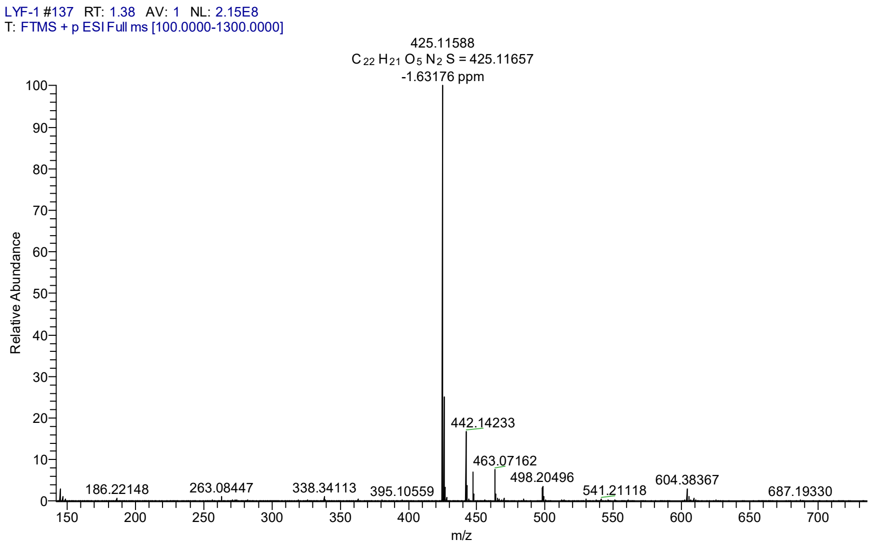

Figure 6. HRMS of compound L2.

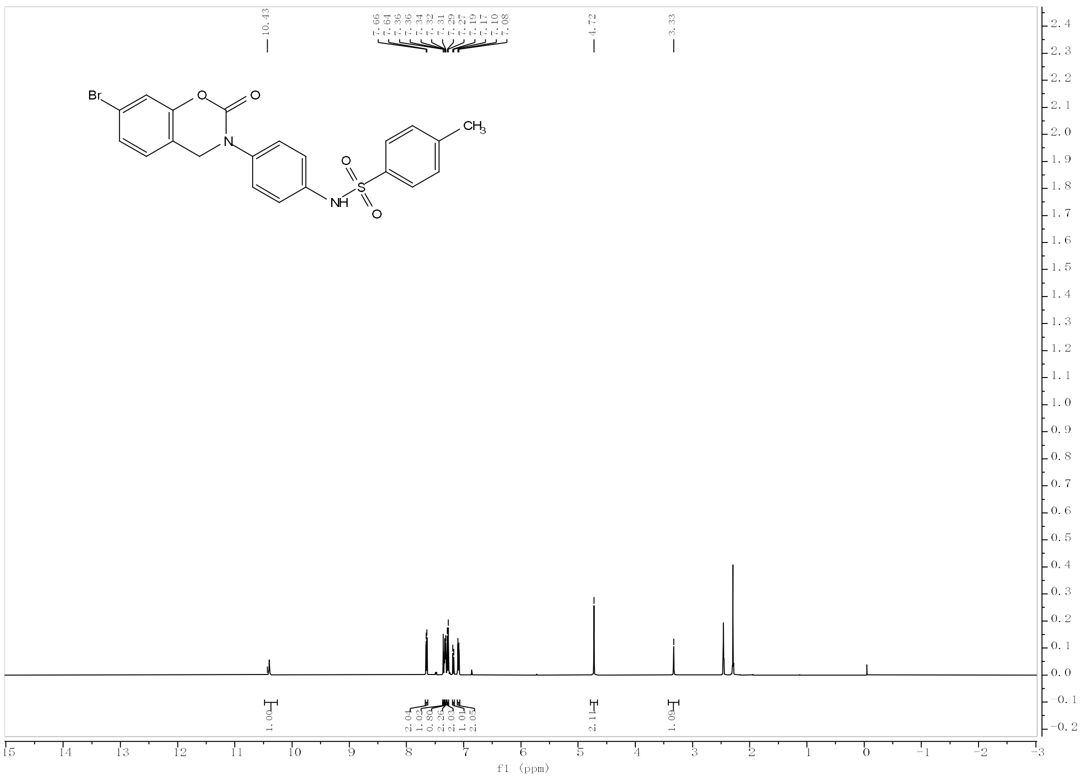

Figure 7. ^{1}H NMR (500 MHz, DMSO-d_6) spectrum of compound L3.

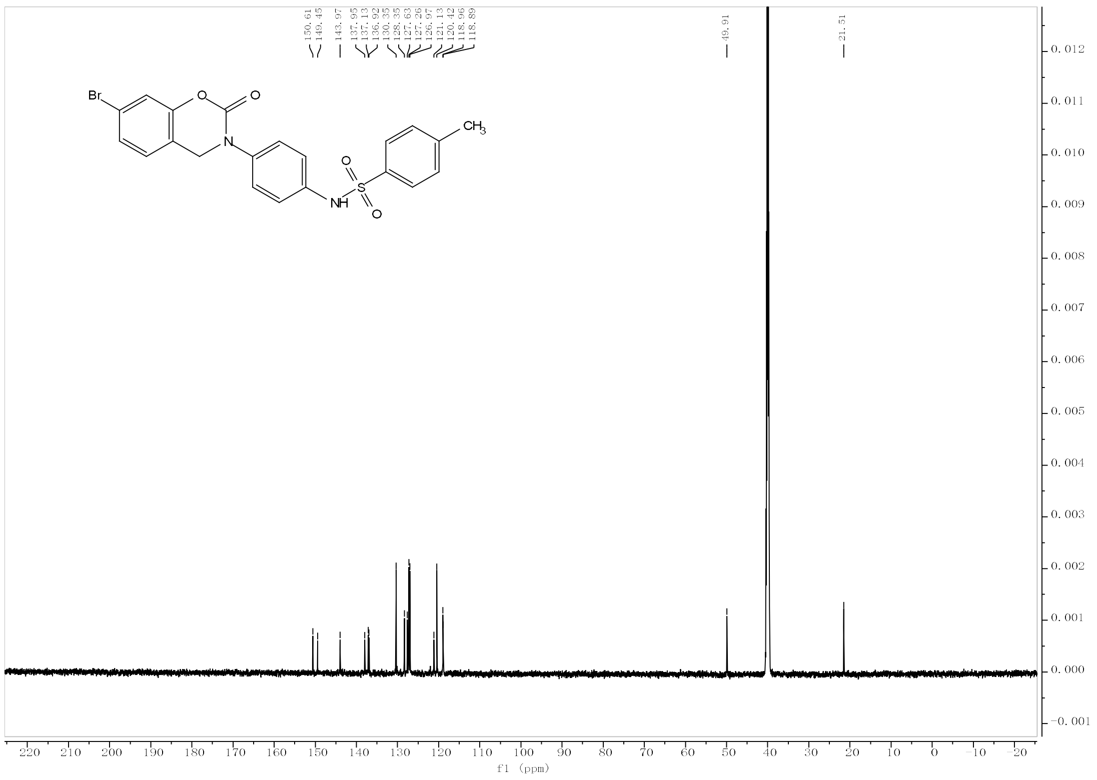

Figure 8. ^{13}C NMR (151 MHz, DMSO-d_6) spectrum of compound L3.

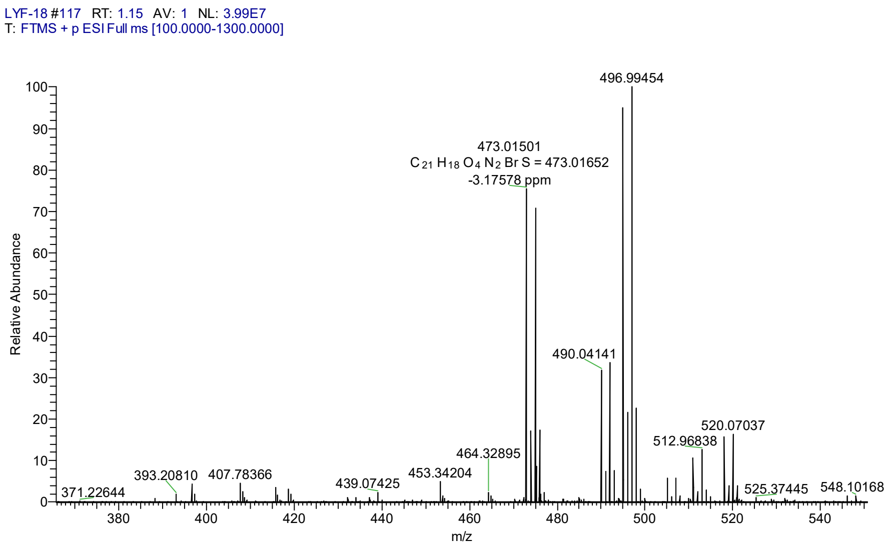

Figure 9. HRMS of compound L3.

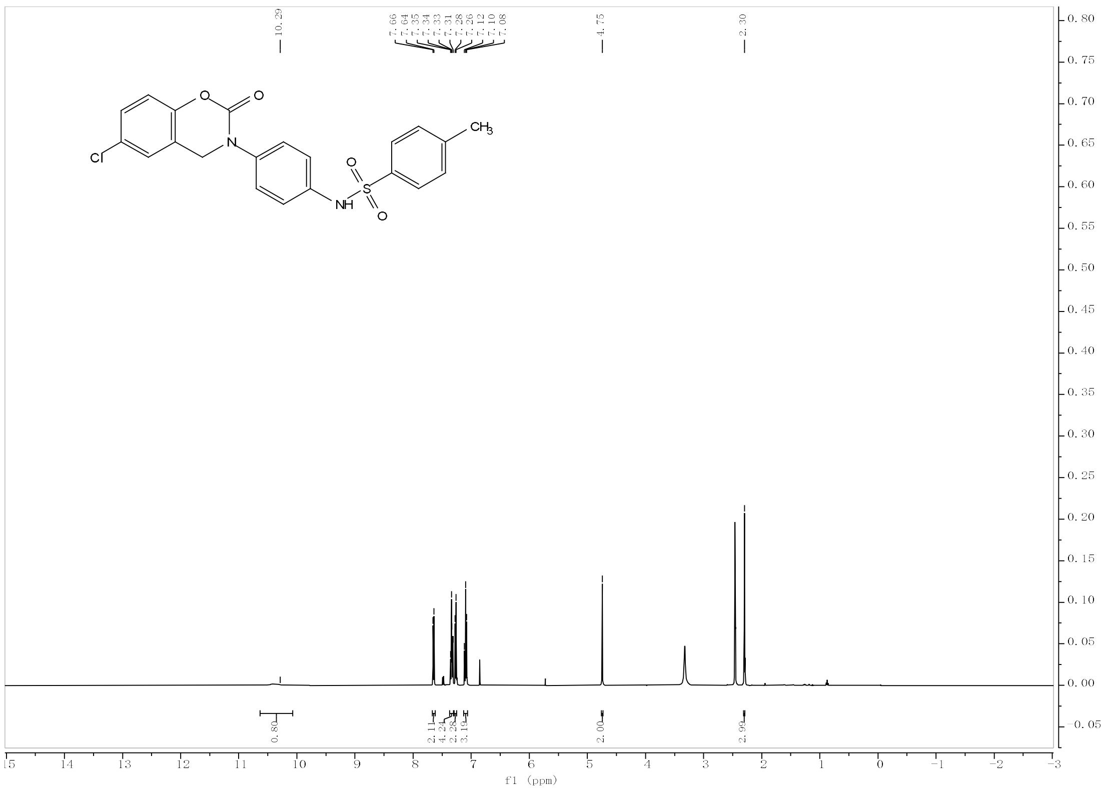

Figure 7. ^{1}H NMR (500 MHz, DMSO-d_6) spectrum of compound L4.

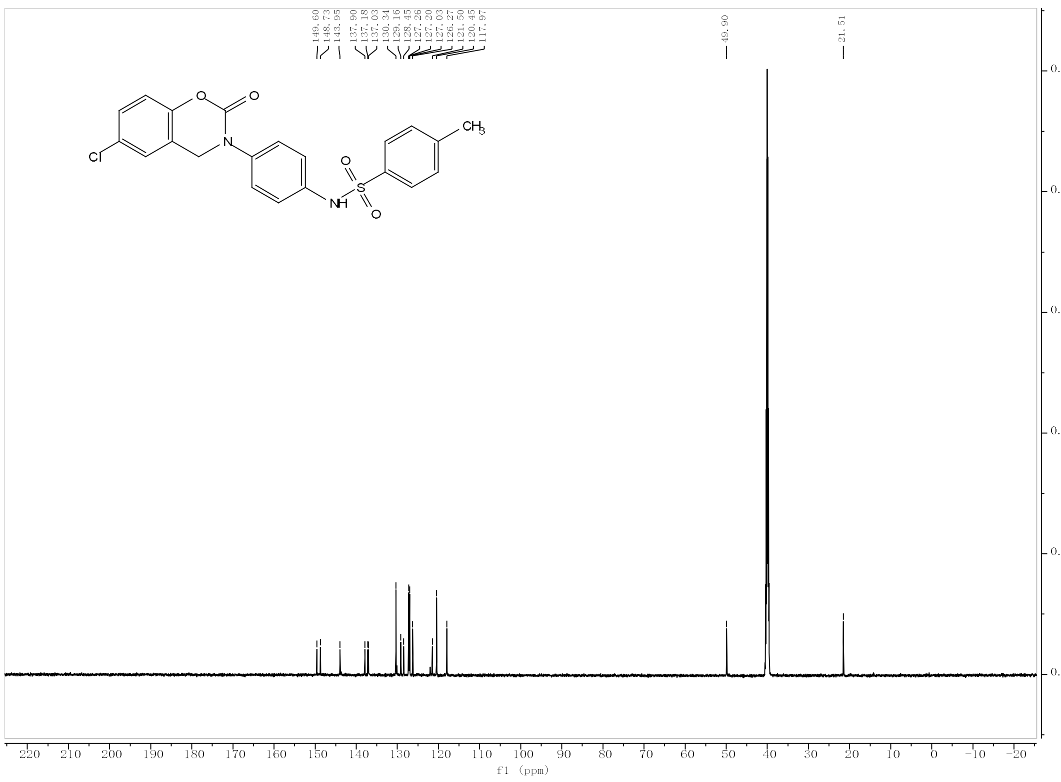

Figure 11. ^{13}C NMR (126 MHz, DMSO-d_6) spectrum of compound L4.

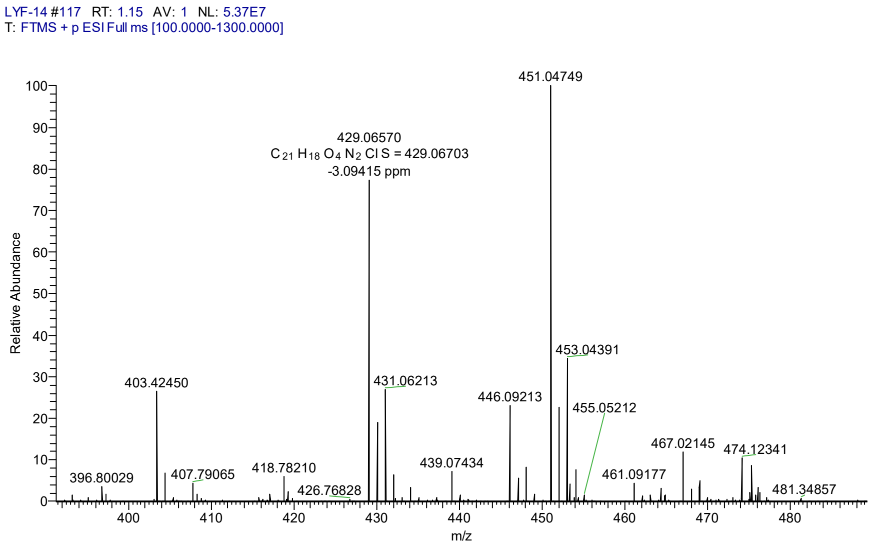

Figure 12. HRMS of compound L4.

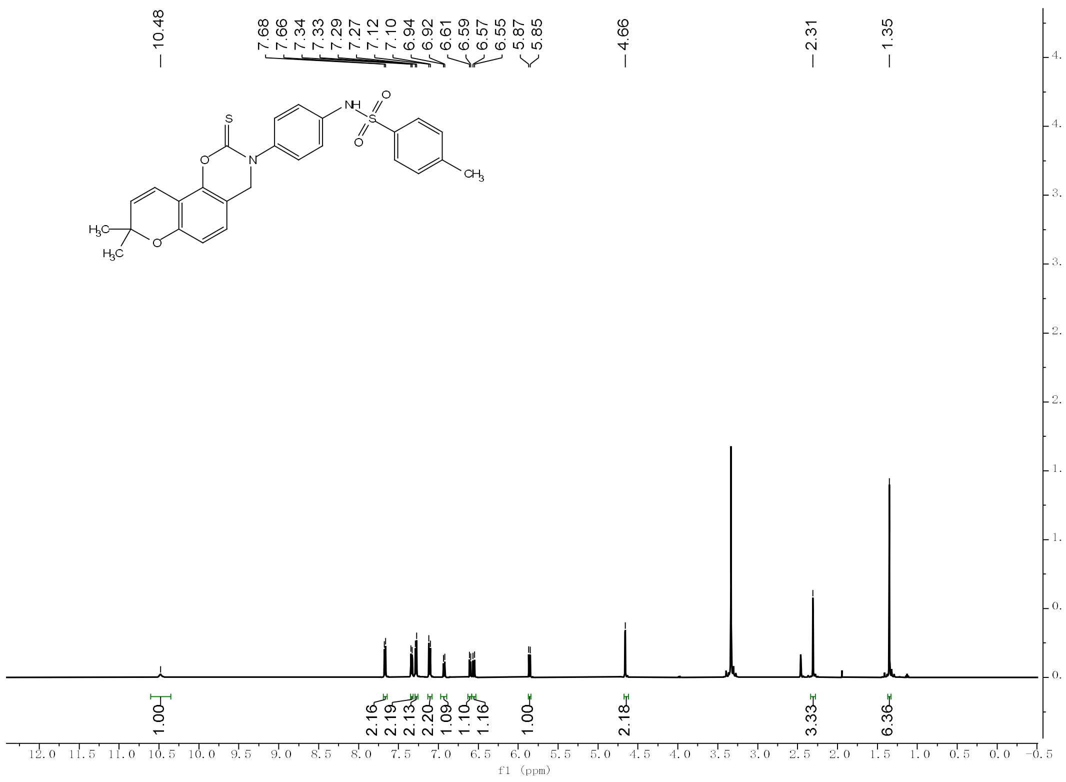

Figure 13. ^{1}H NMR (500 MHz, DMSO-d_6) spectrum of compound L5.

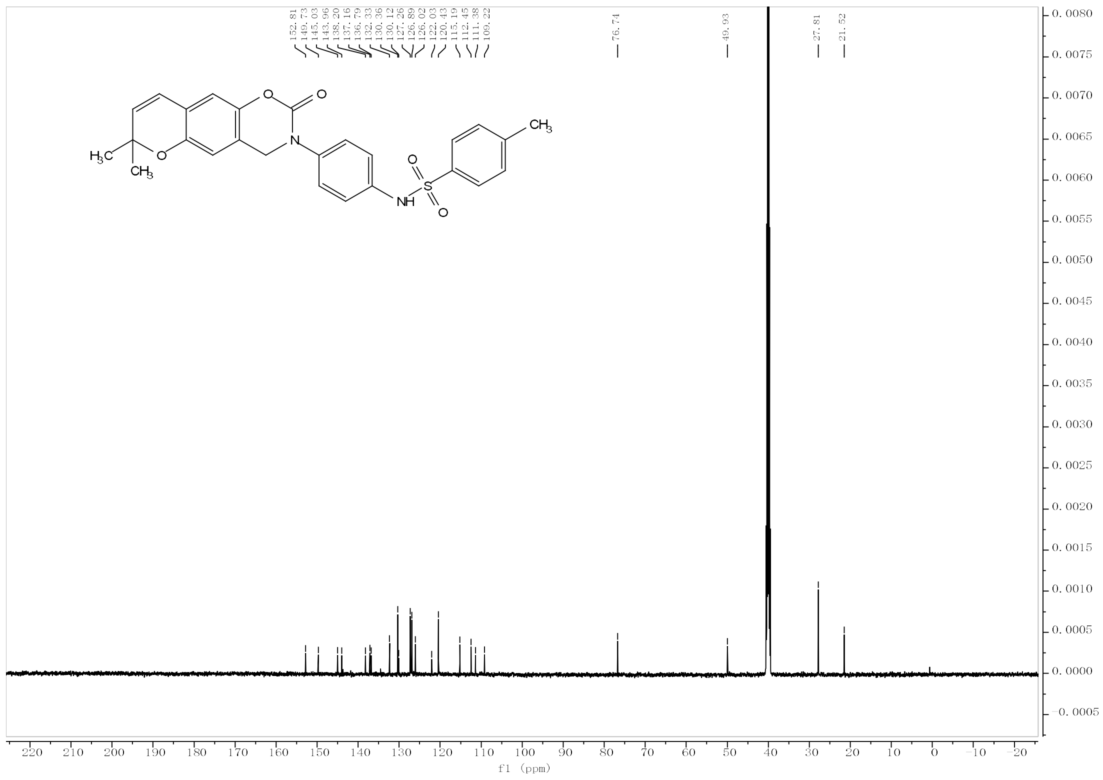

Figure 14. ^{13}C NMR (126 MHz, DMSO-d_6) spectrum of compound L5.

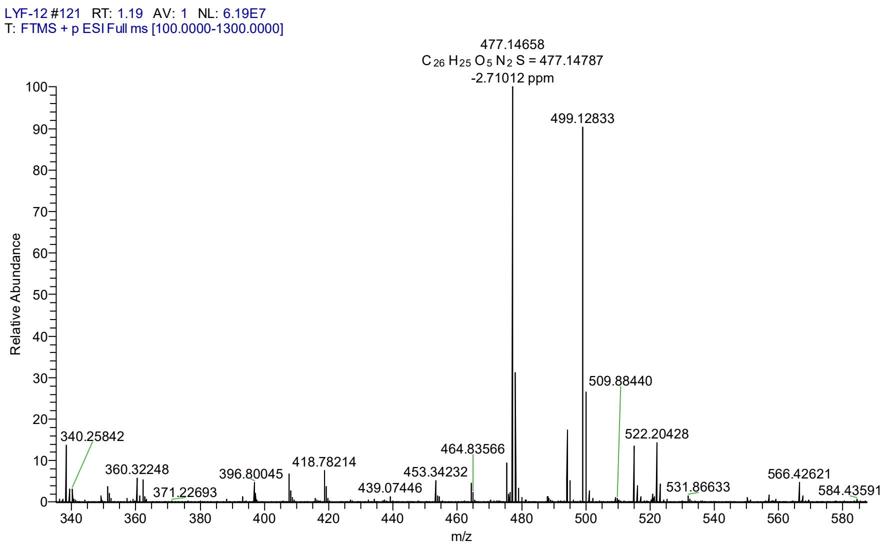

Figure 15. HRMS of compound L5.

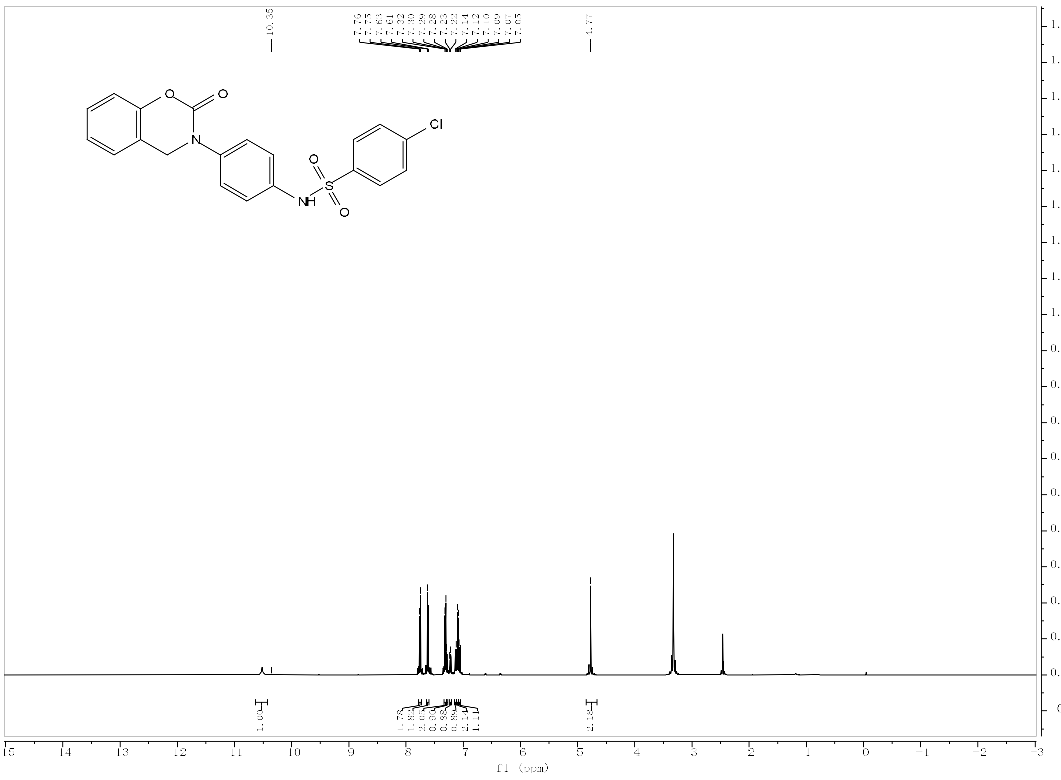

Figure 16. ^{1}H NMR (600 MHz, DMSO-d_6) spectrum of compound L6.

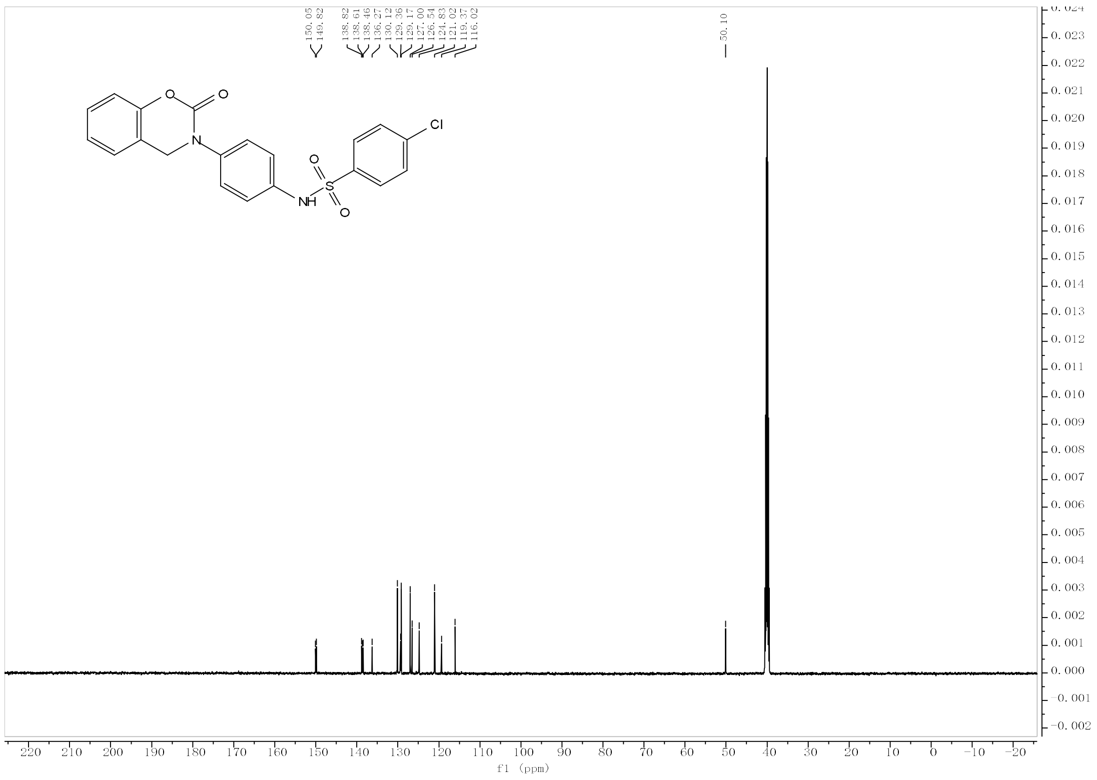

Figure 17. ^{13}C NMR (126 MHz, DMSO-d_6) spectrum of compound L6.

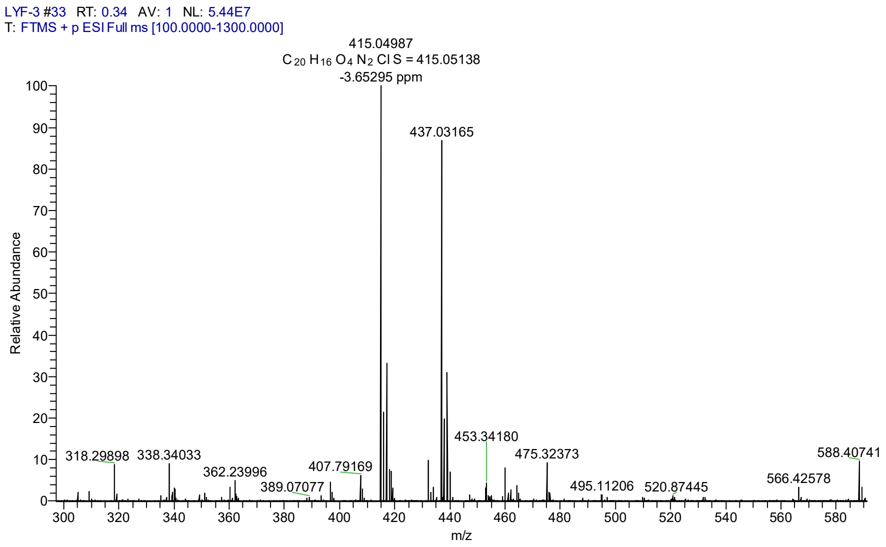

Figure 18. HRMS of compound L6.

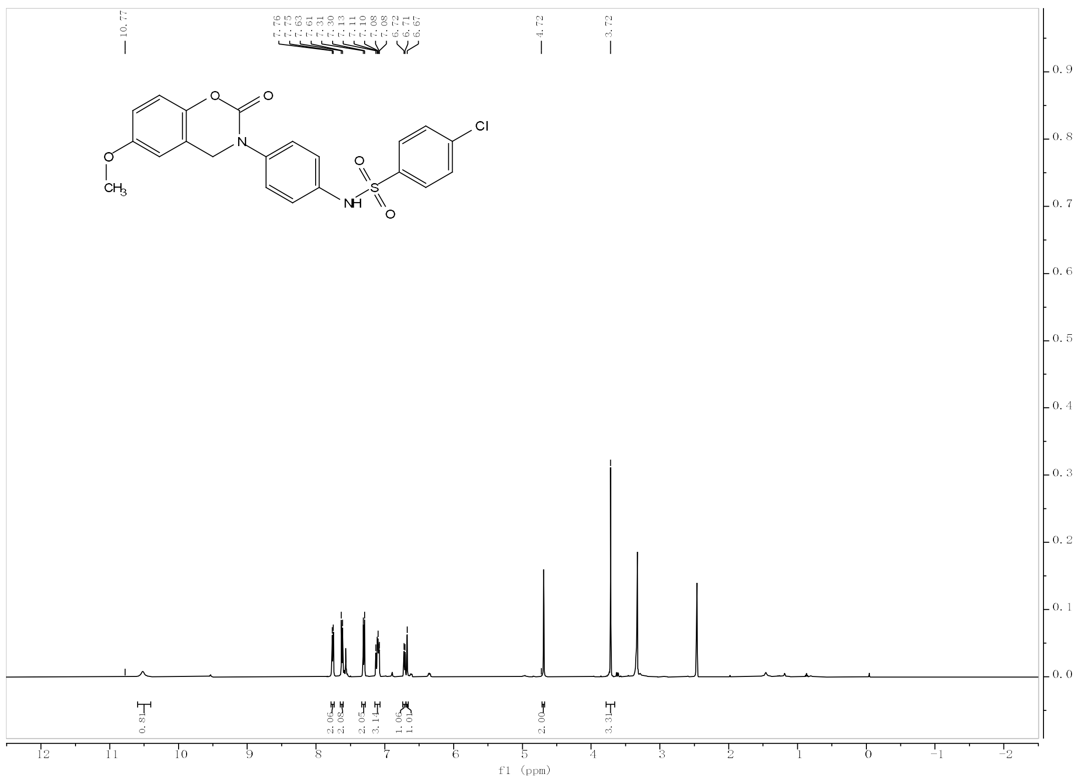

Figure 19. ^{1}H NMR (600 MHz, DMSO-d_6) spectrum of compound L7.

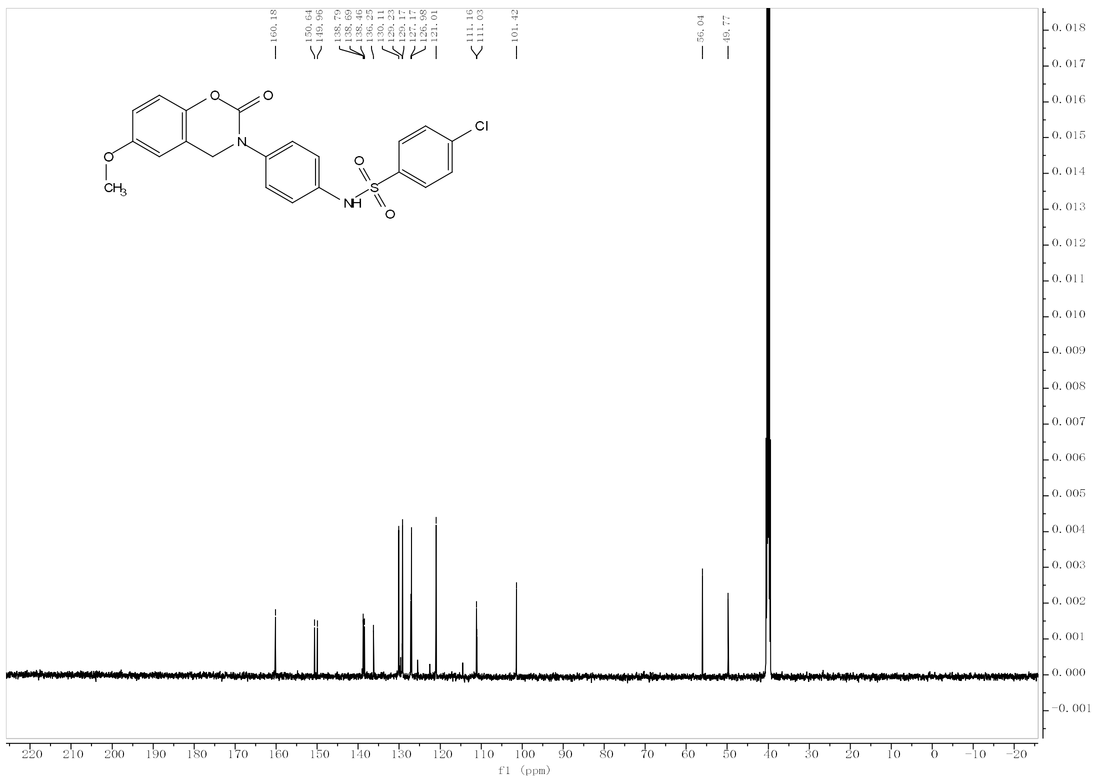

Figure 20. ^{13}C NMR (126 MHz, DMSO-d_6) spectrum of compound L7.

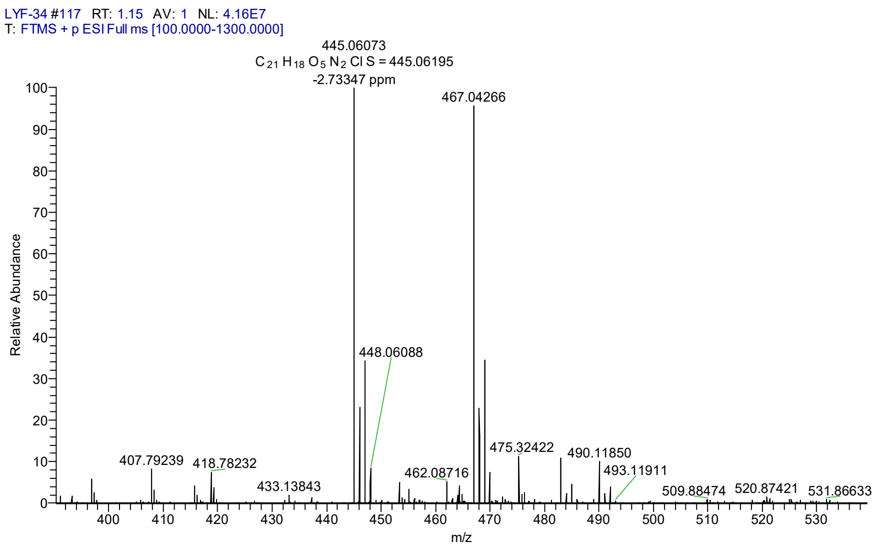

Figure 21. HRMS of compound L7.

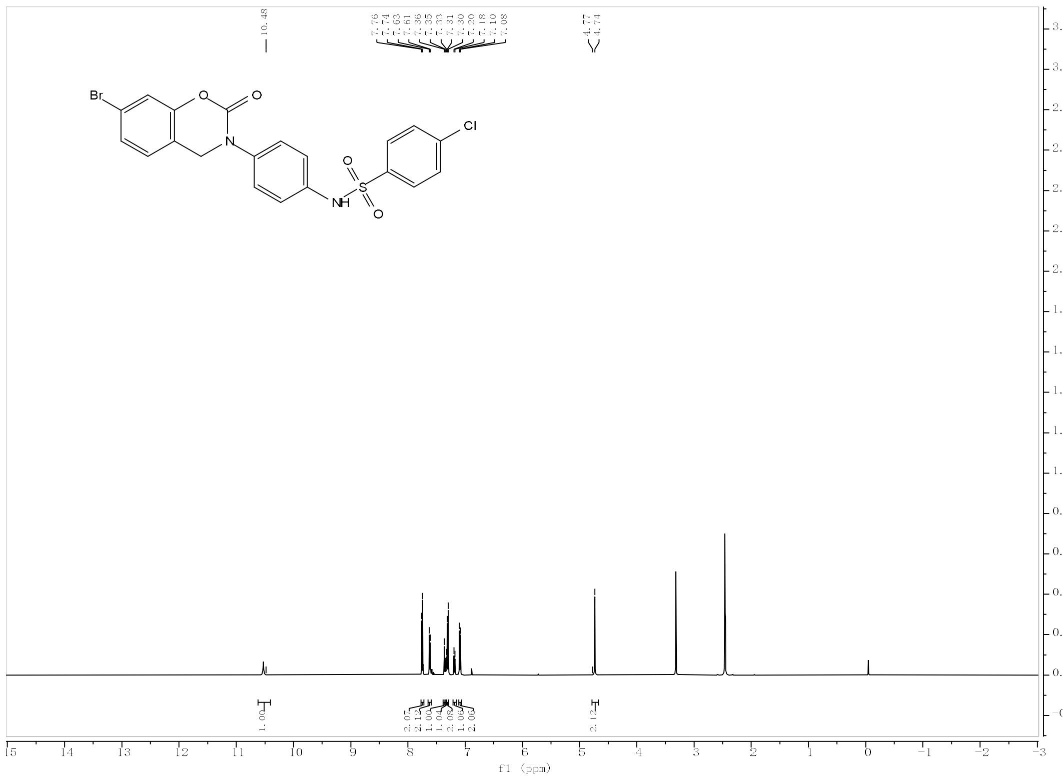

Figure 22. ^{1}H NMR (500 MHz, DMSO-d_6) spectrum of compound L8.

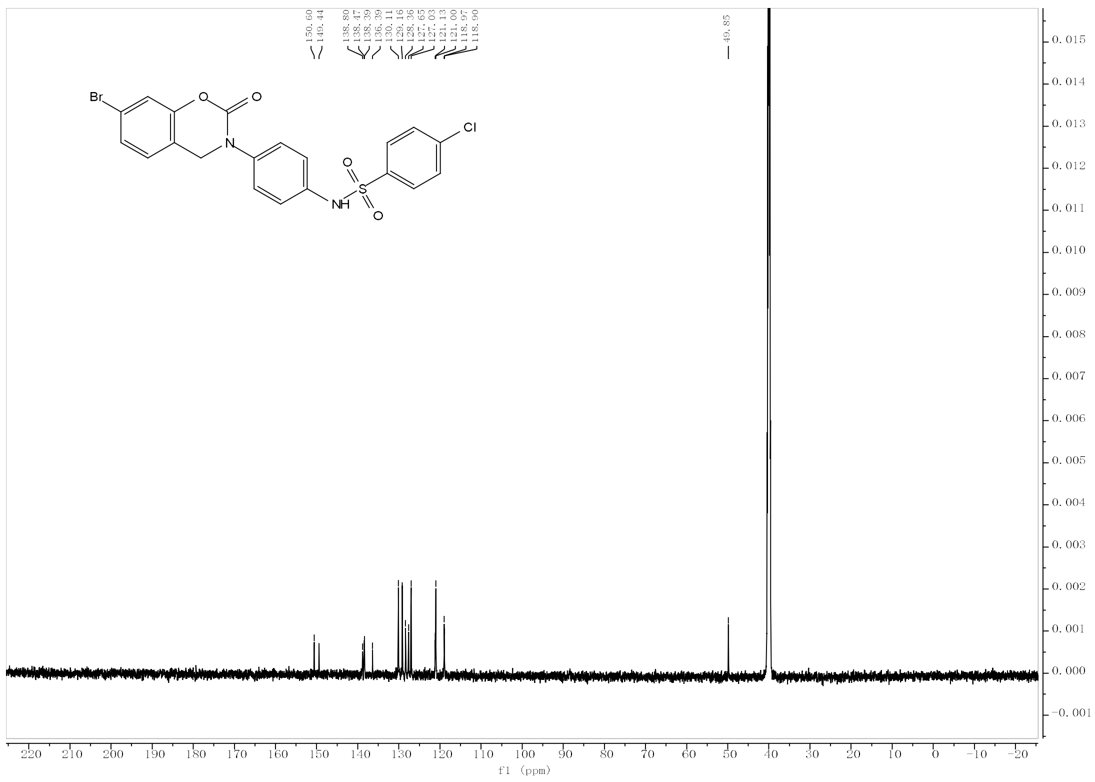

Figure 23. ^{13}C NMR (151 MHz, DMSO-d_6) spectrum of compound L8.

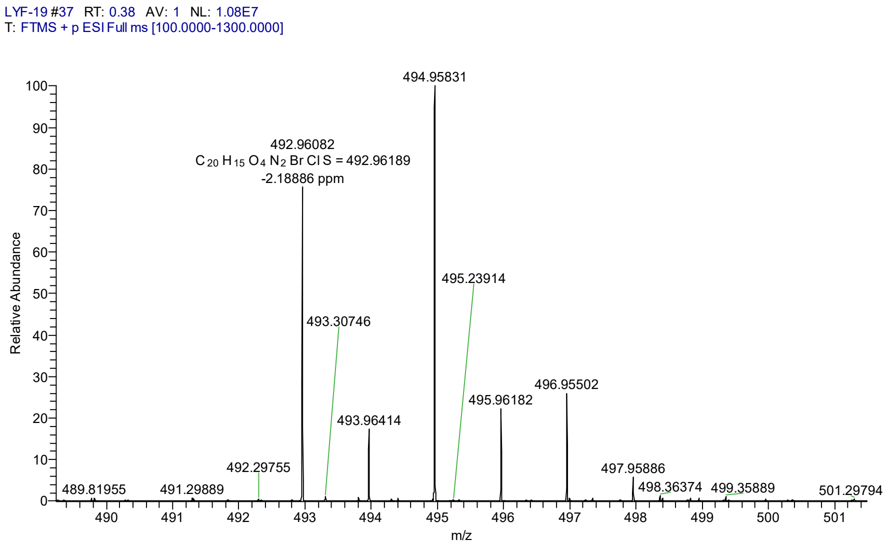

Figure 24. HRMS of compound L8.

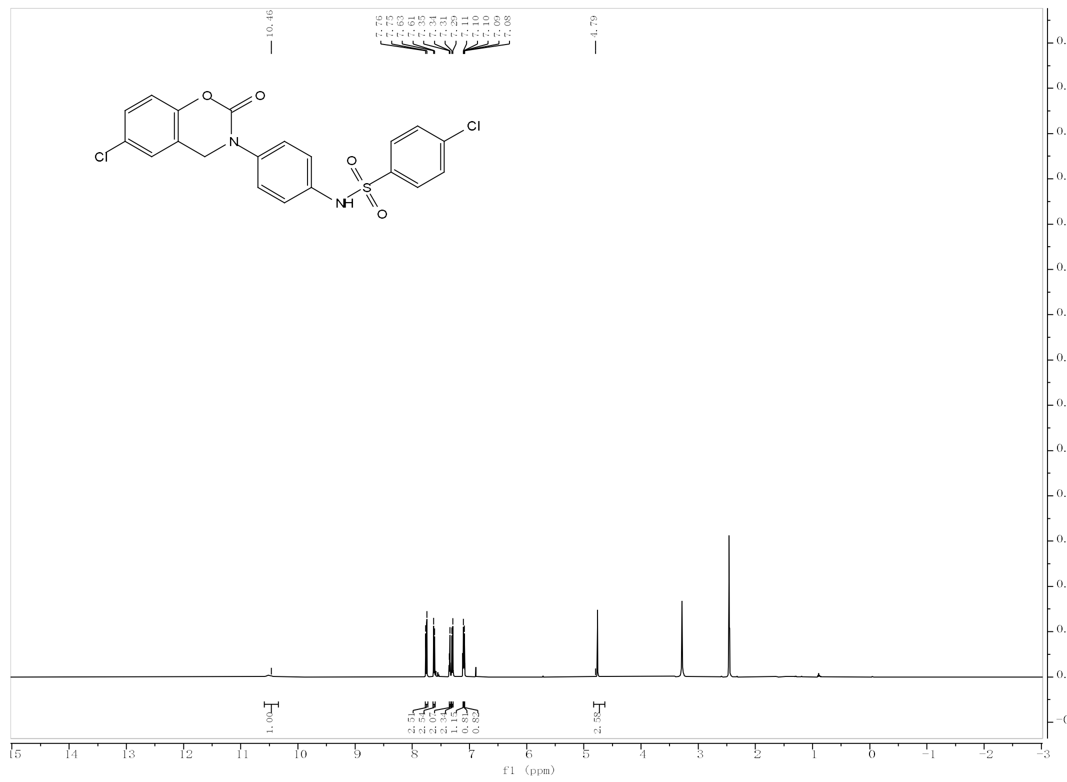

Figure 22. ^{1}H NMR (500 MHz, DMSO-d_6) spectrum of compound L9.

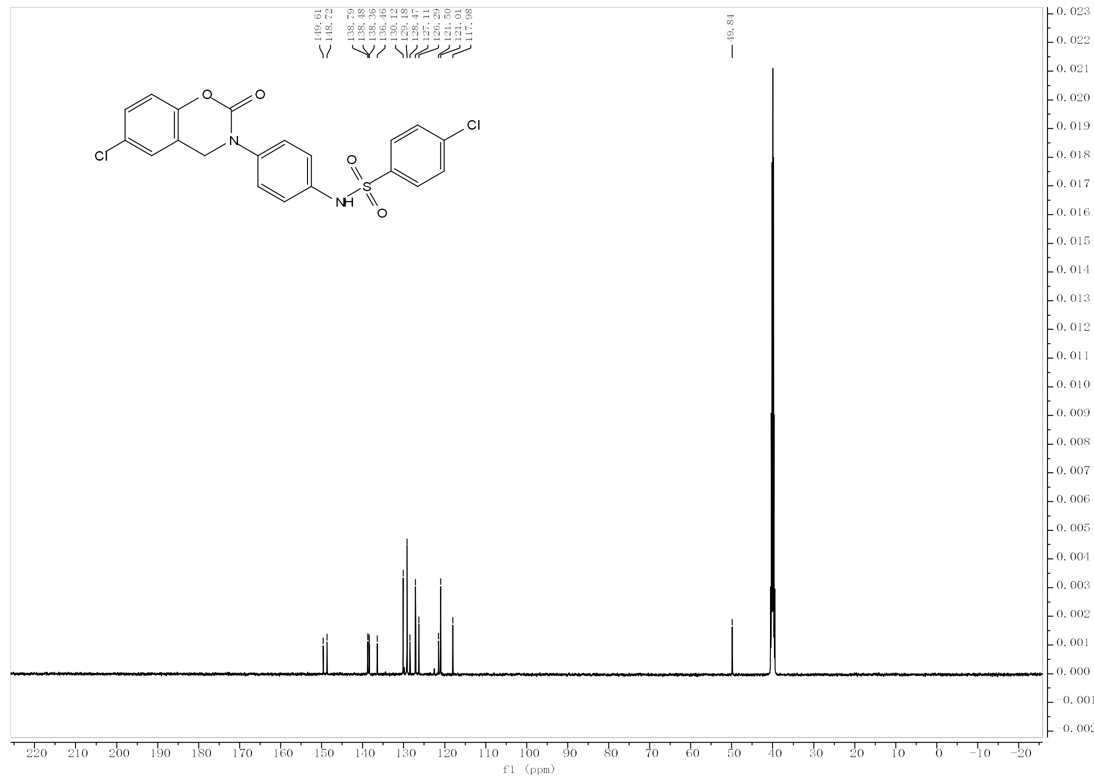

Figure 26. ^{13}C NMR (126 MHz, DMSO-d_6) spectrum of compound L9.

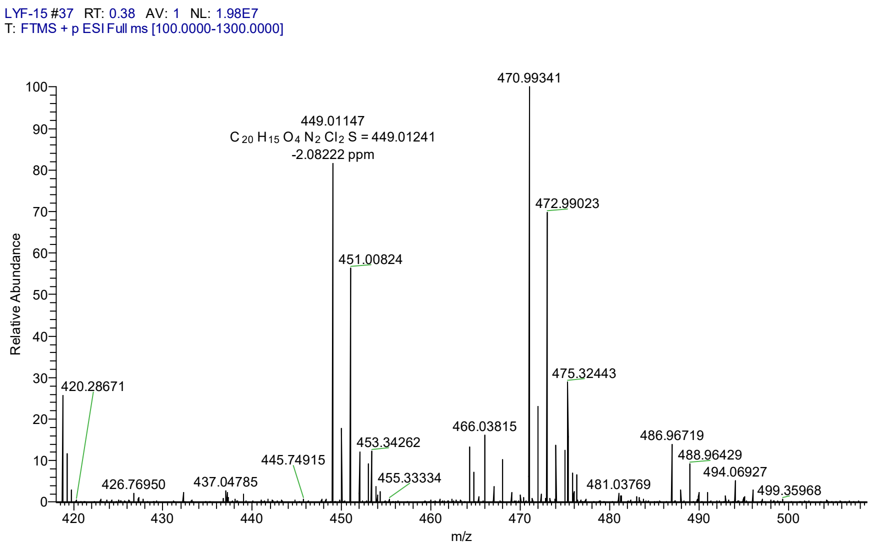

Figure 27. HRMS of compound L9.

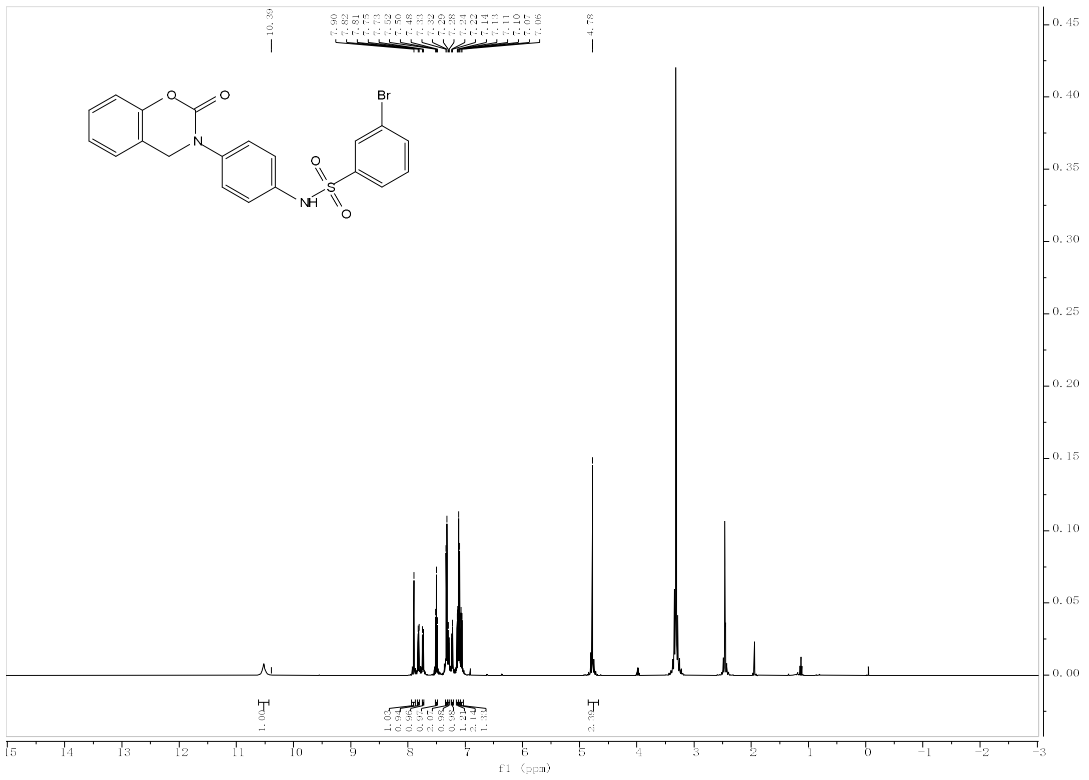

Figure 28. ^{1}H NMR (600 MHz, DMSO-d_6) spectrum of compound L10.

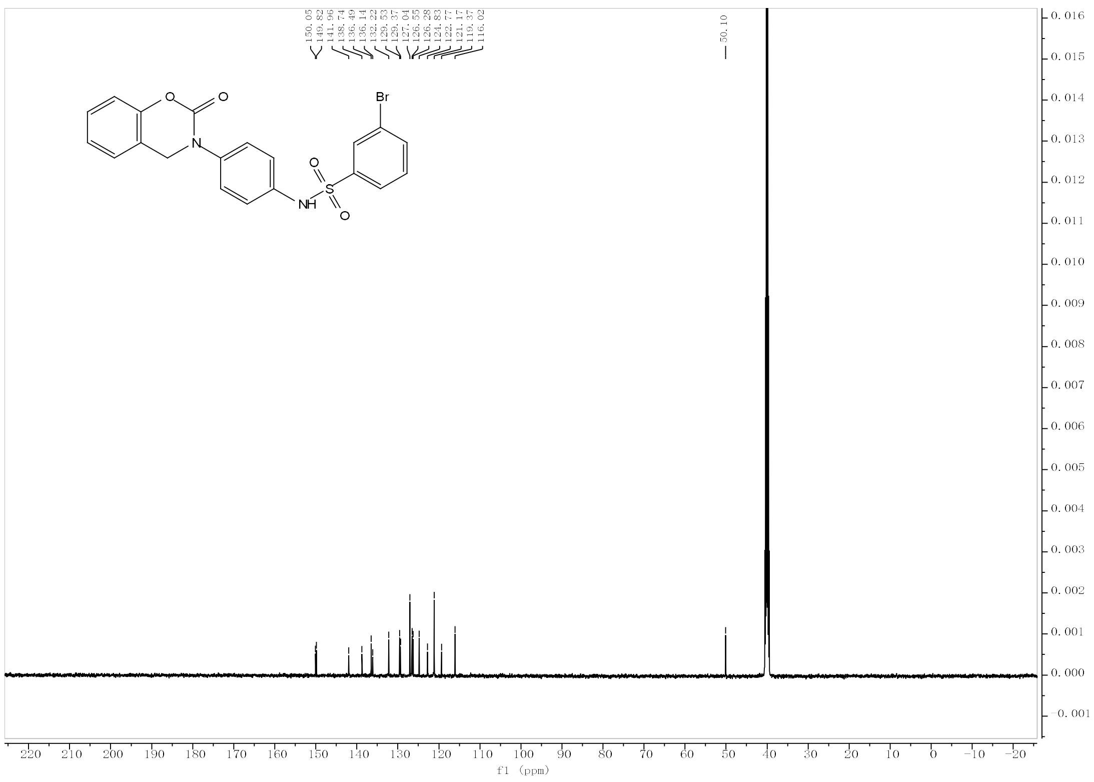

Figure 29. ^{13}C NMR (126 MHz, DMSO-d_6) spectrum of compound L10.

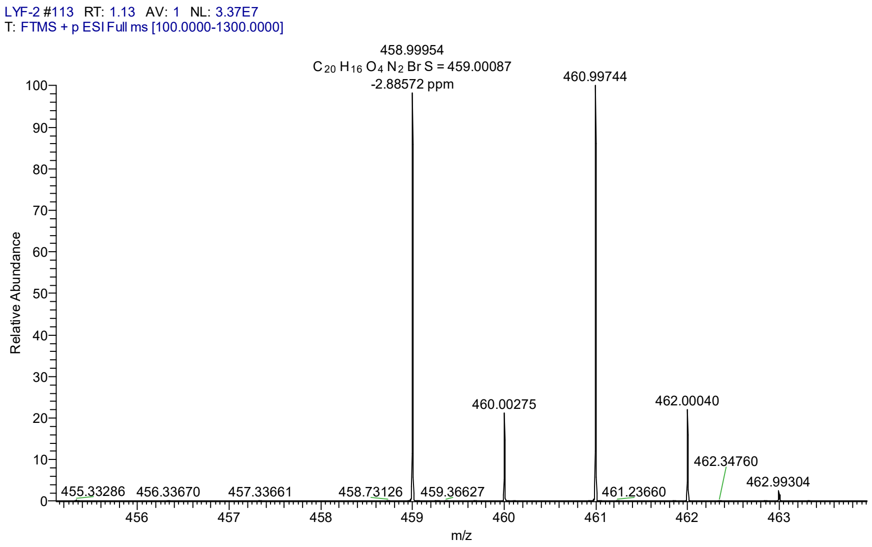

Figure 30. HRMS of compound L10.

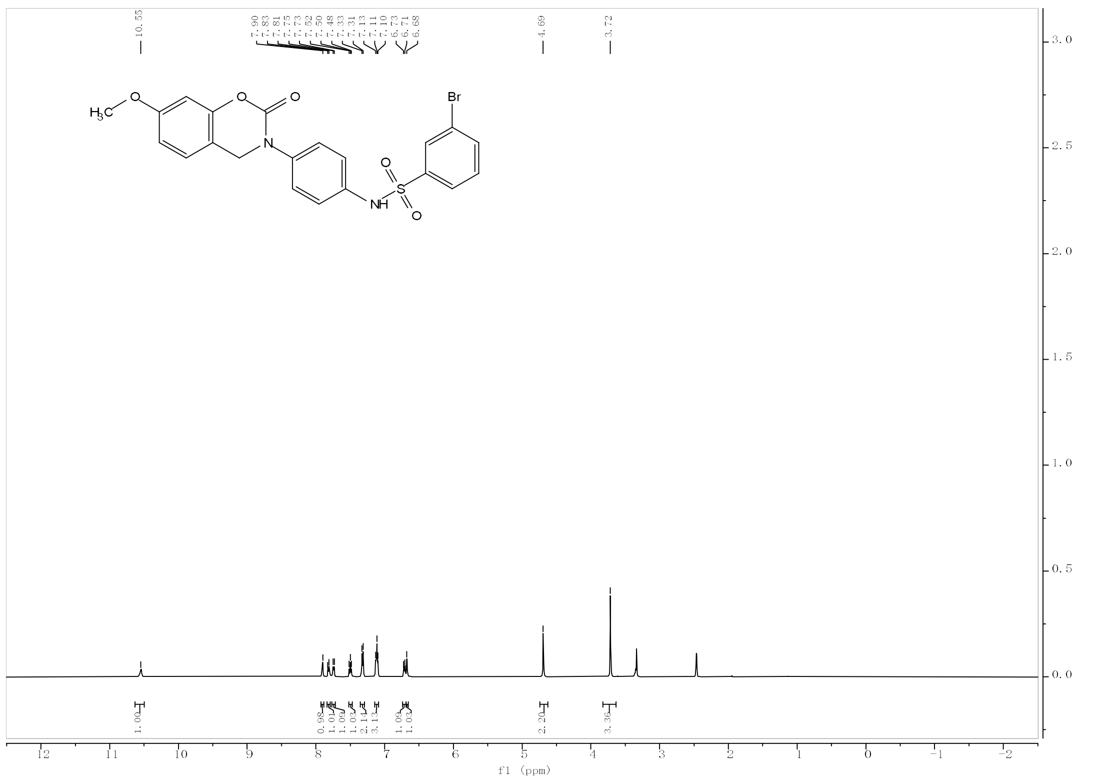

Figure 31. ^{1}H NMR (500 MHz, DMSO-d_6) spectrum of compound L11.

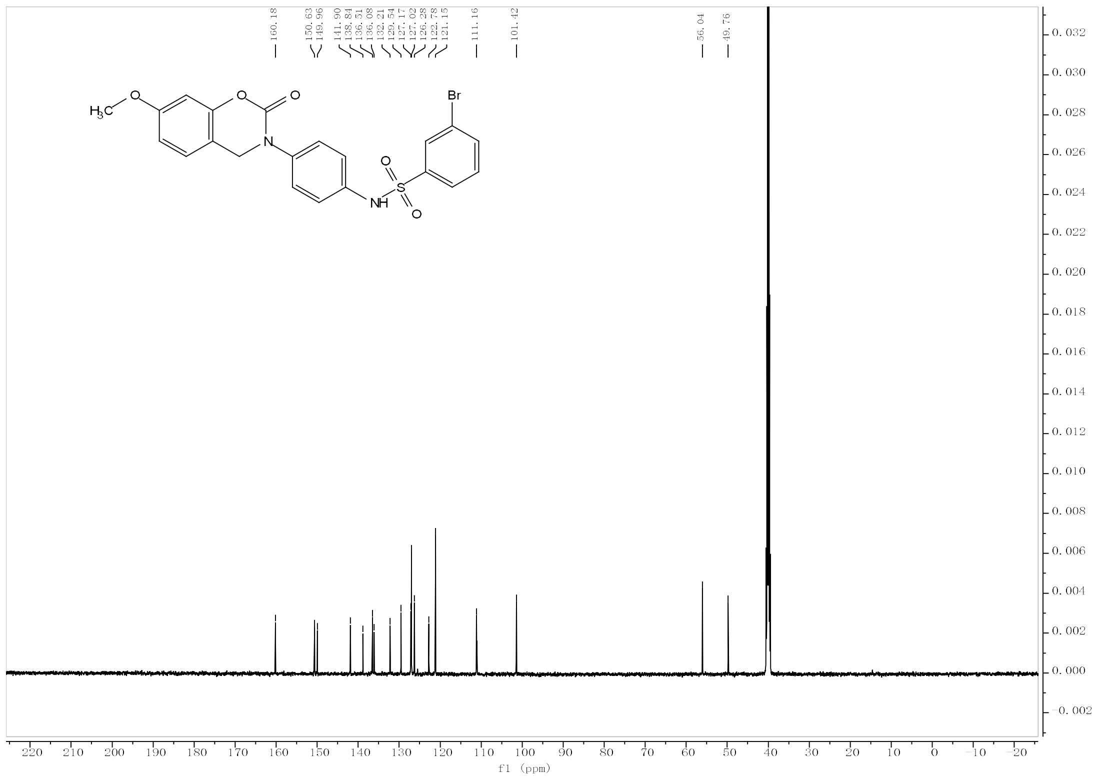

Figure 32. ^{13}C NMR (126 MHz, DMSO-d_6) spectrum of compound L11.

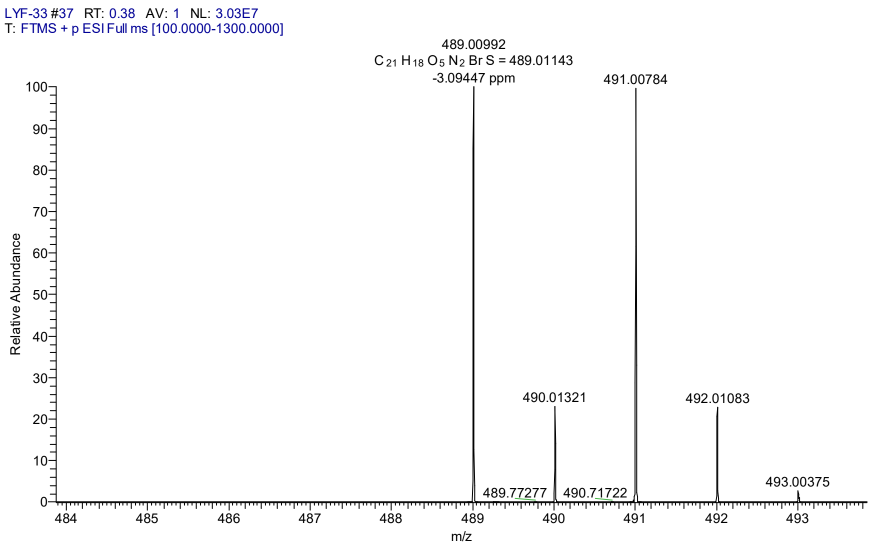

Figure 33. HRMS of compound L11.

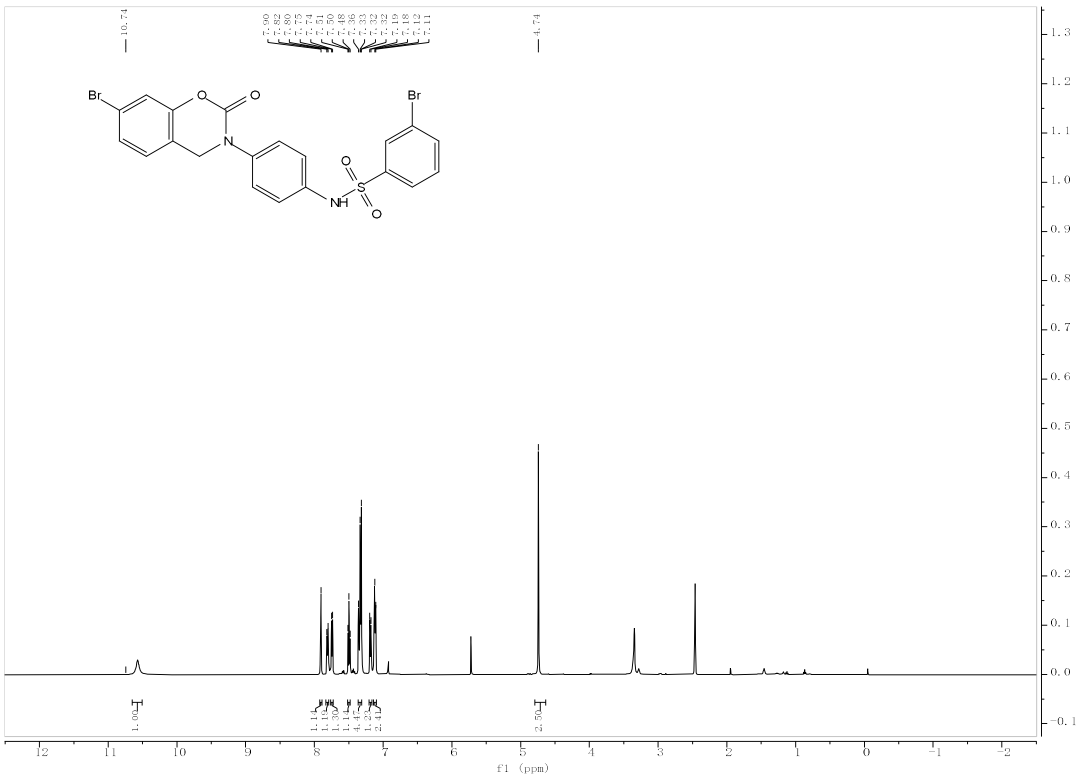

Figure 34. ^{1}H NMR (500 MHz, DMSO-d_6) spectrum of compound L12.

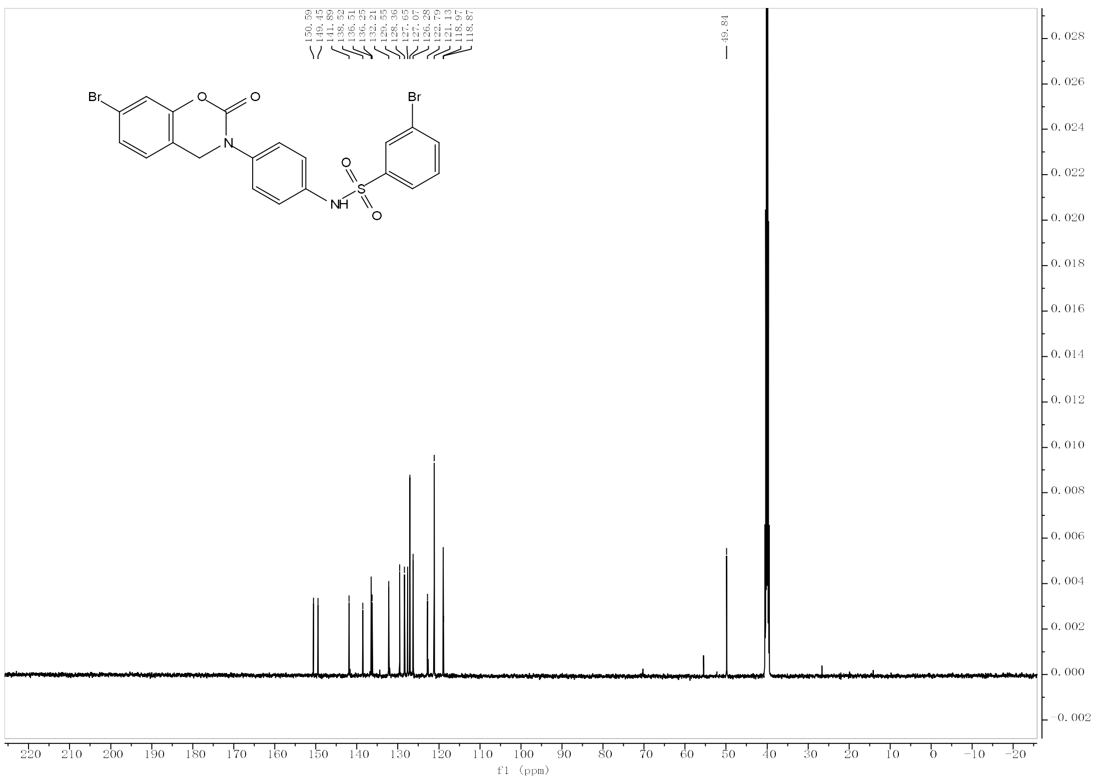

Figure 35. ^{13}C NMR (126 MHz, DMSO-d_6) spectrum of compound L12.

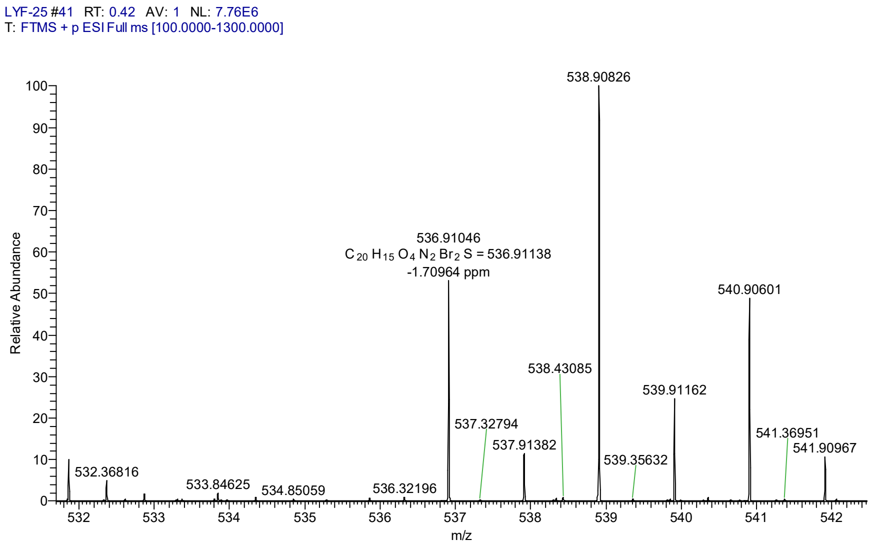

Figure 36. HRMS of compound L12.

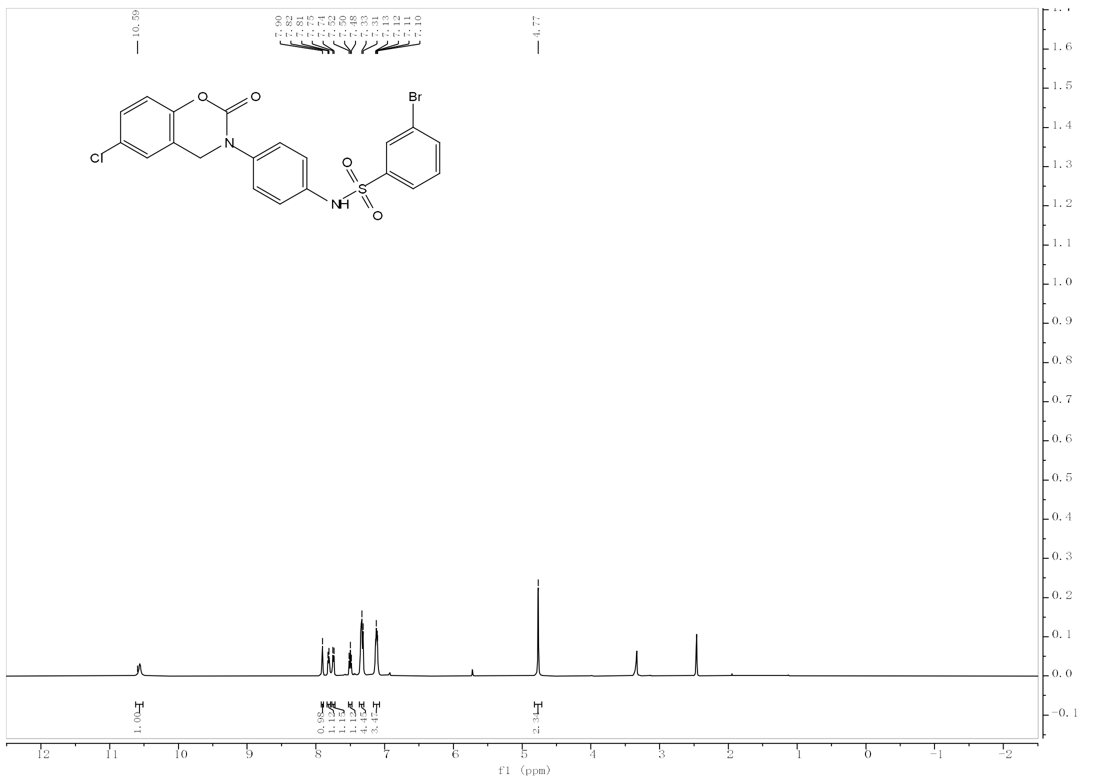

Figure 37 ^{1}H NMR (500 MHz, DMSO- d_6) spectrum of compound L13.

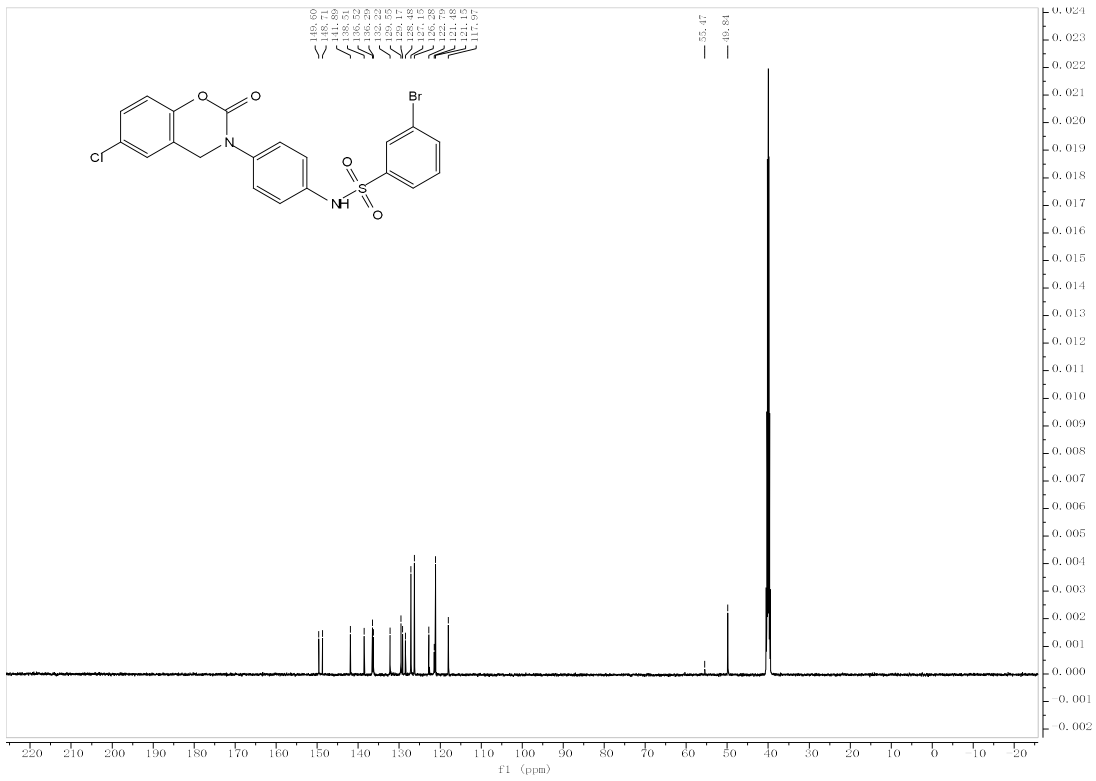

Figure 38 ^{13}C NMR (126 MHz, DMSO- d_6) spectrum of compound L13.

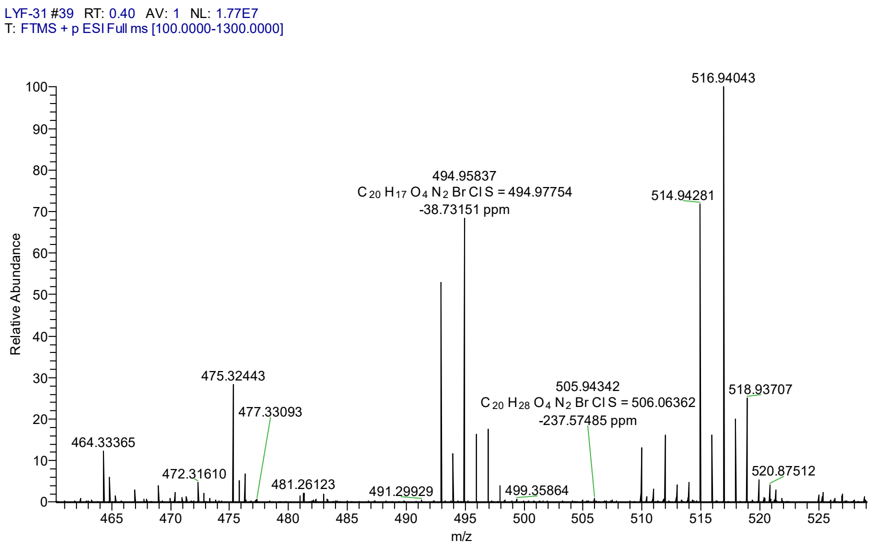

Figure 39. HRMS of compound L13.

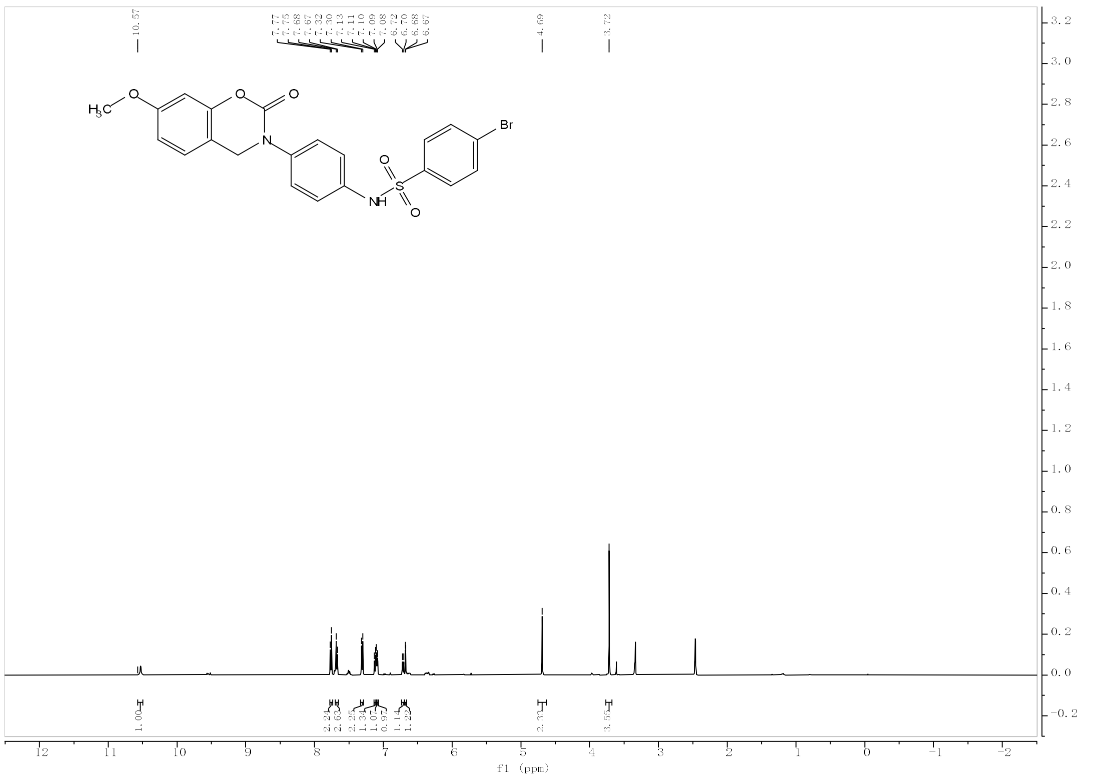

Figure 40 ^{1}H NMR (500 MHz, DMSO- d_6) spectrum of compound L14.

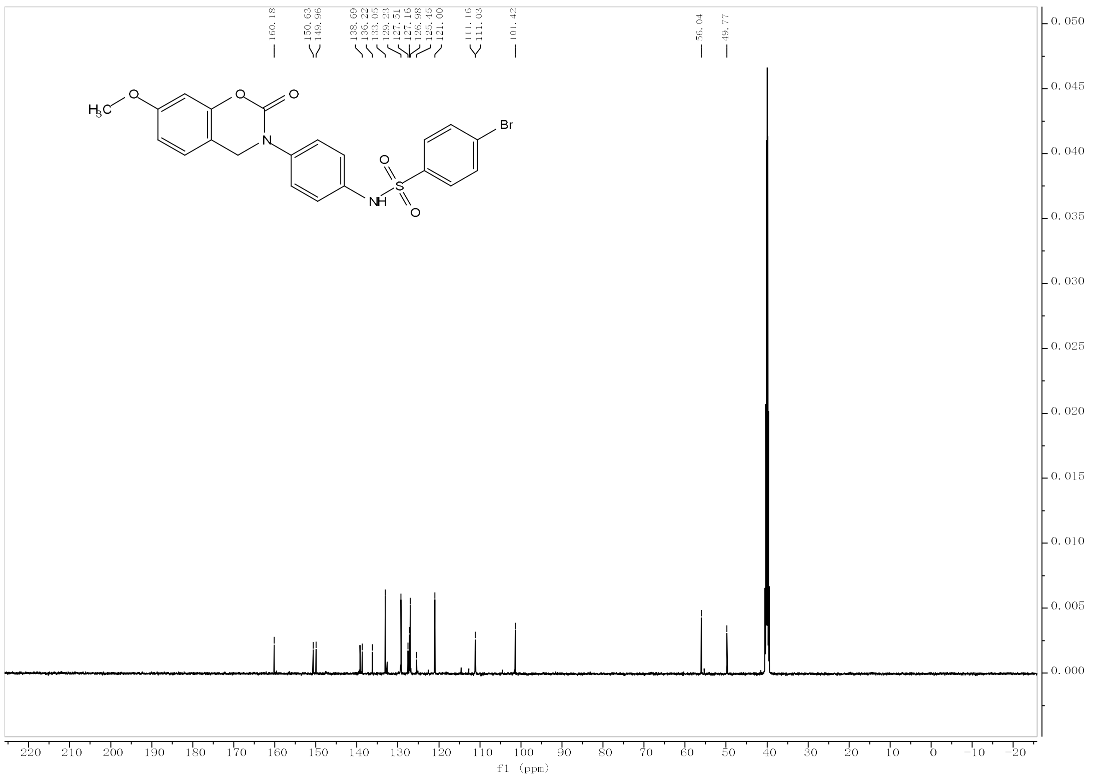

Figure 41 ^{13}C NMR (126 MHz, DMSO- d_6) spectrum of compound L14.

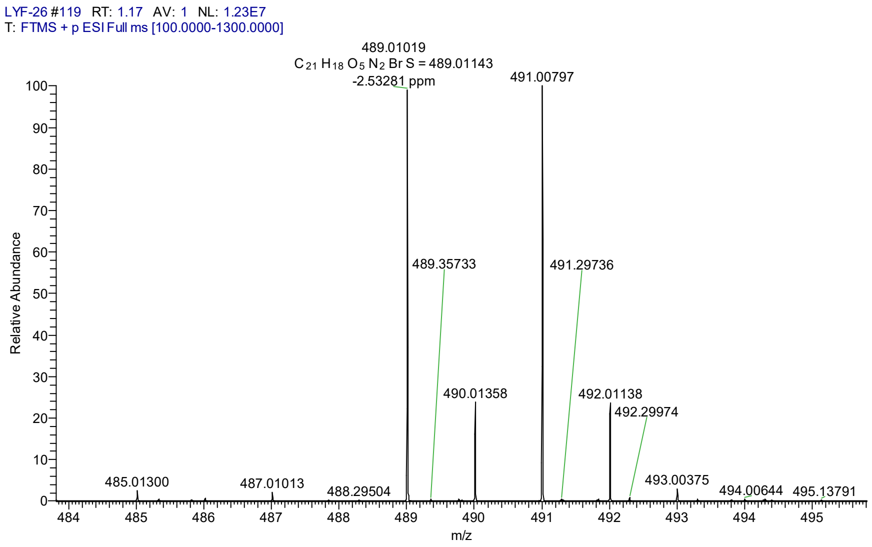

Figure 42. HRMS of compound L14.

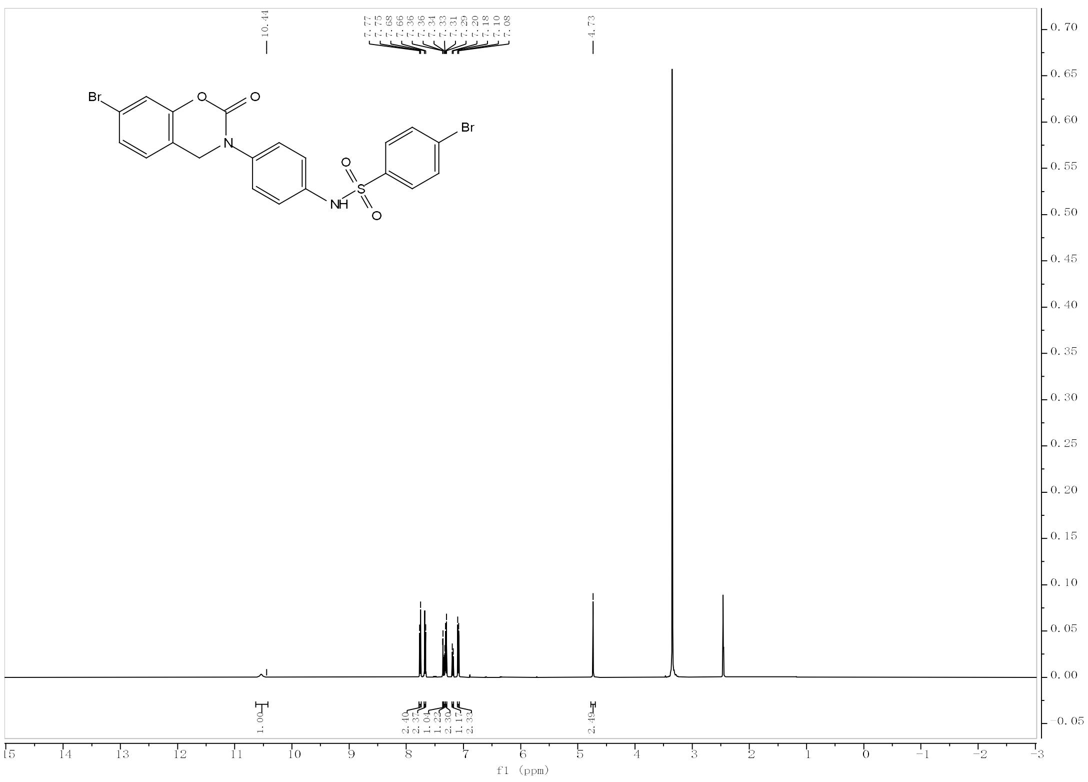

Figure 43 ^{1}H NMR (500 MHz, DMSO- d_6) spectrum of compound L15.

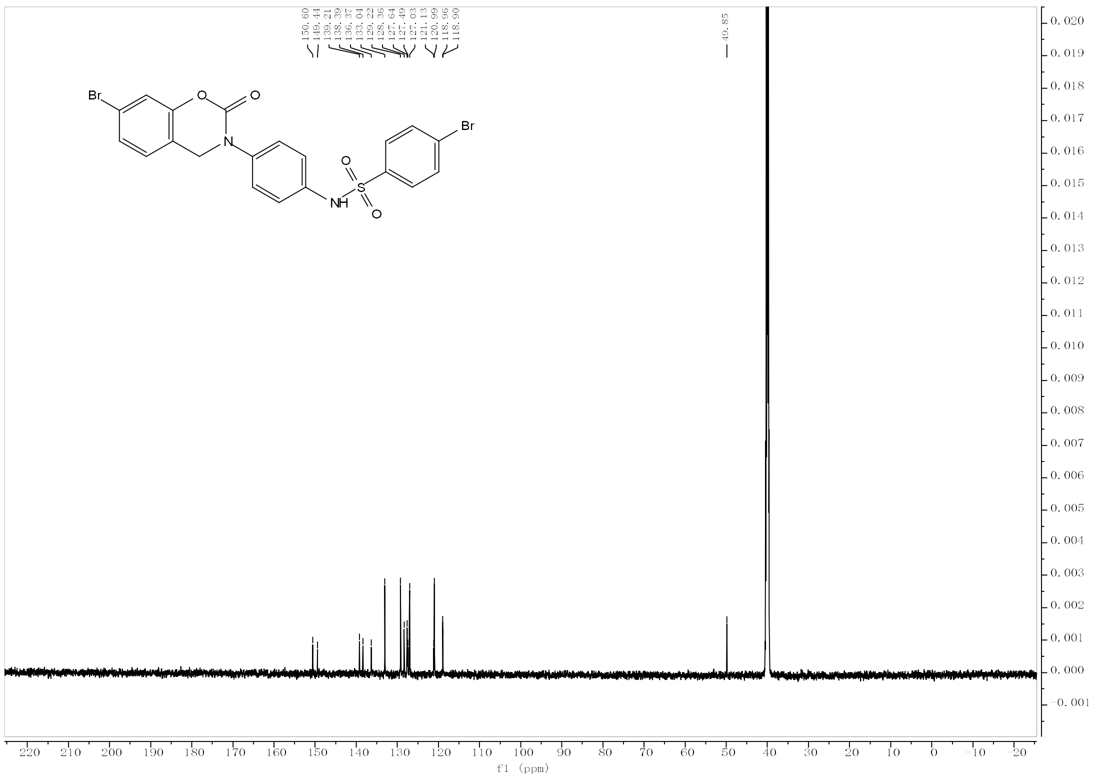

Figure 44 ^{13}C NMR (151 MHz, DMSO- d_6) spectrum of compound L15.

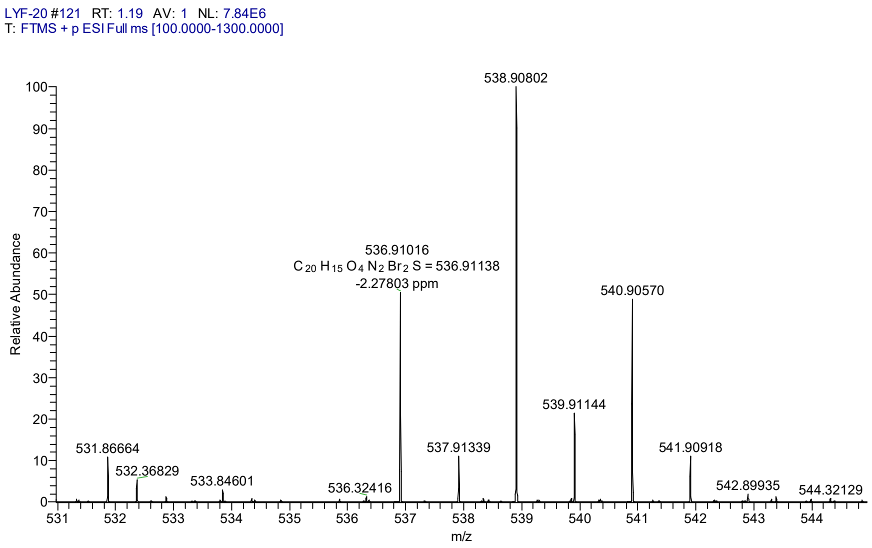

Figure 45. HRMS of compound L15.

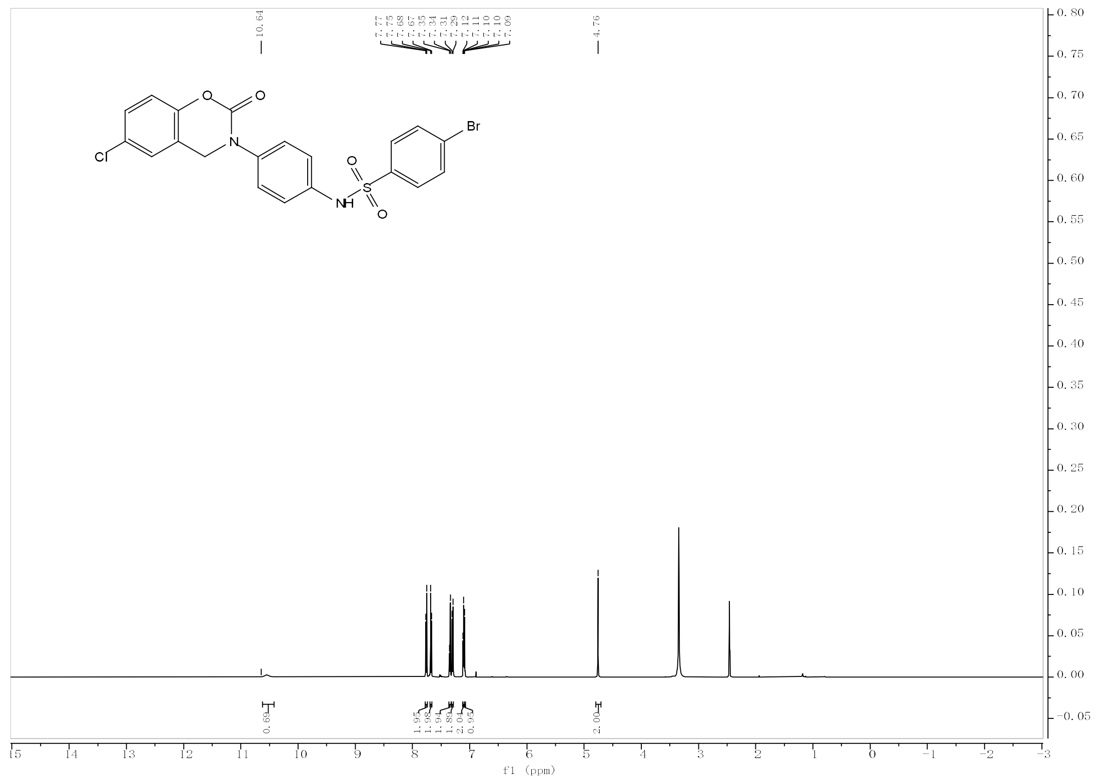

Figure 46 ^{1}H NMR (600 MHz, DMSO- d_6) spectrum of compound L16.

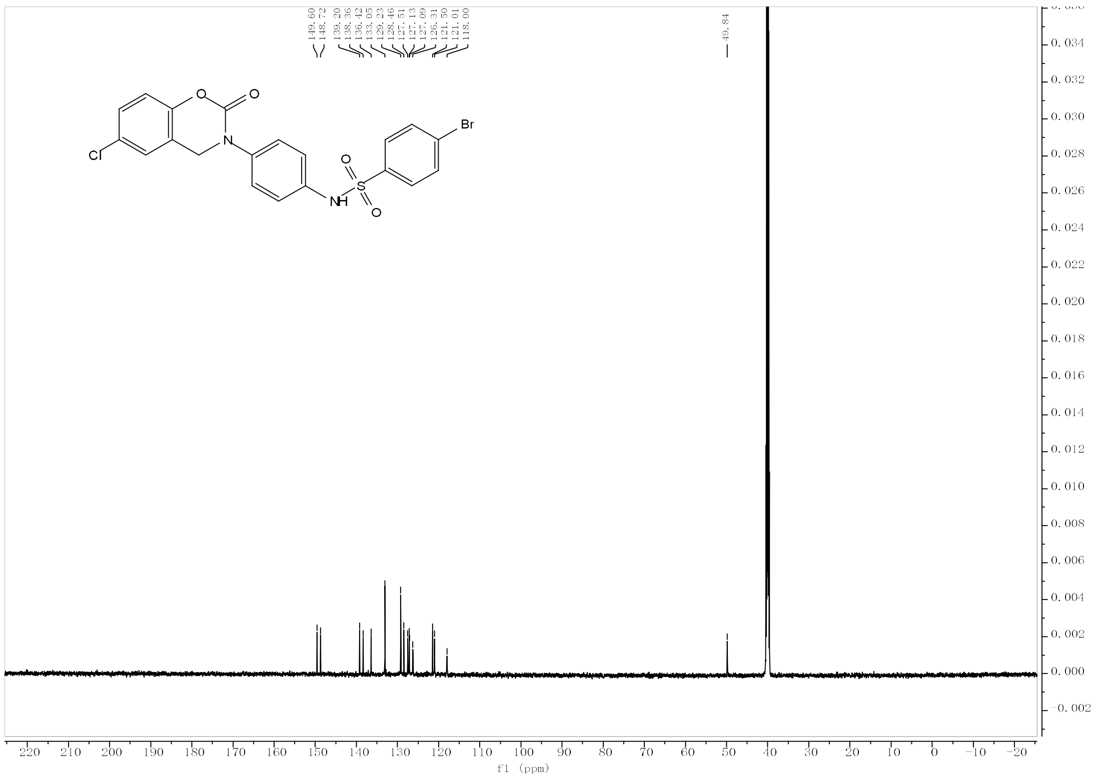

Figure 47 ^{13}C NMR (151 MHz, DMSO- d_6) spectrum of compound L16.

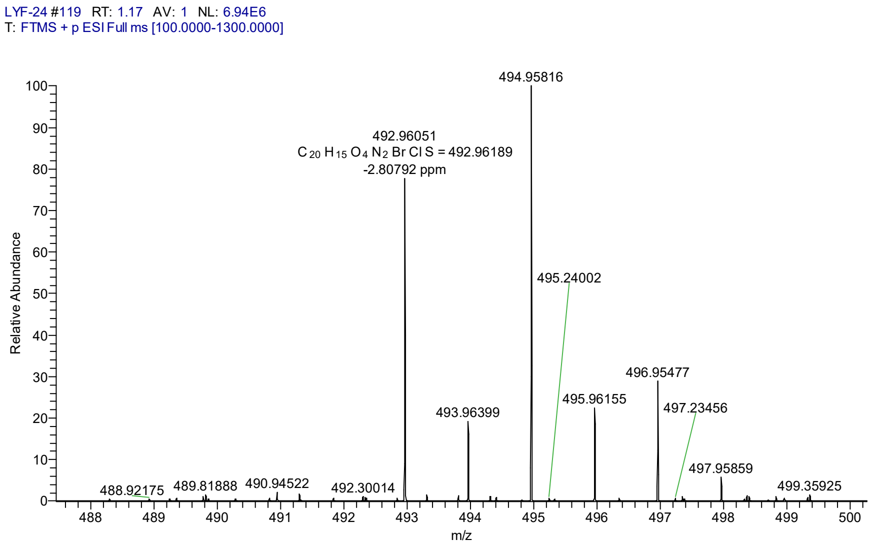

Figure 48. HRMS of compound L16.

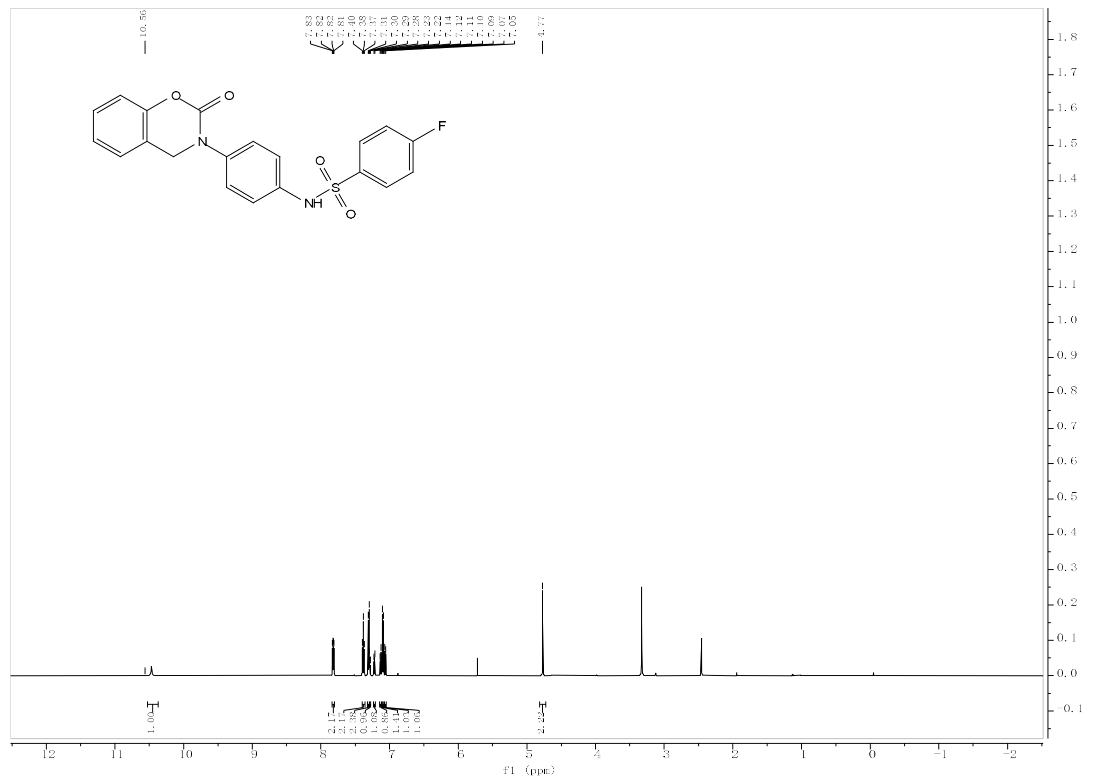

Figure 49. ^{1}H NMR (600 MHz, DMSO- d_6) spectrum of compound L17.

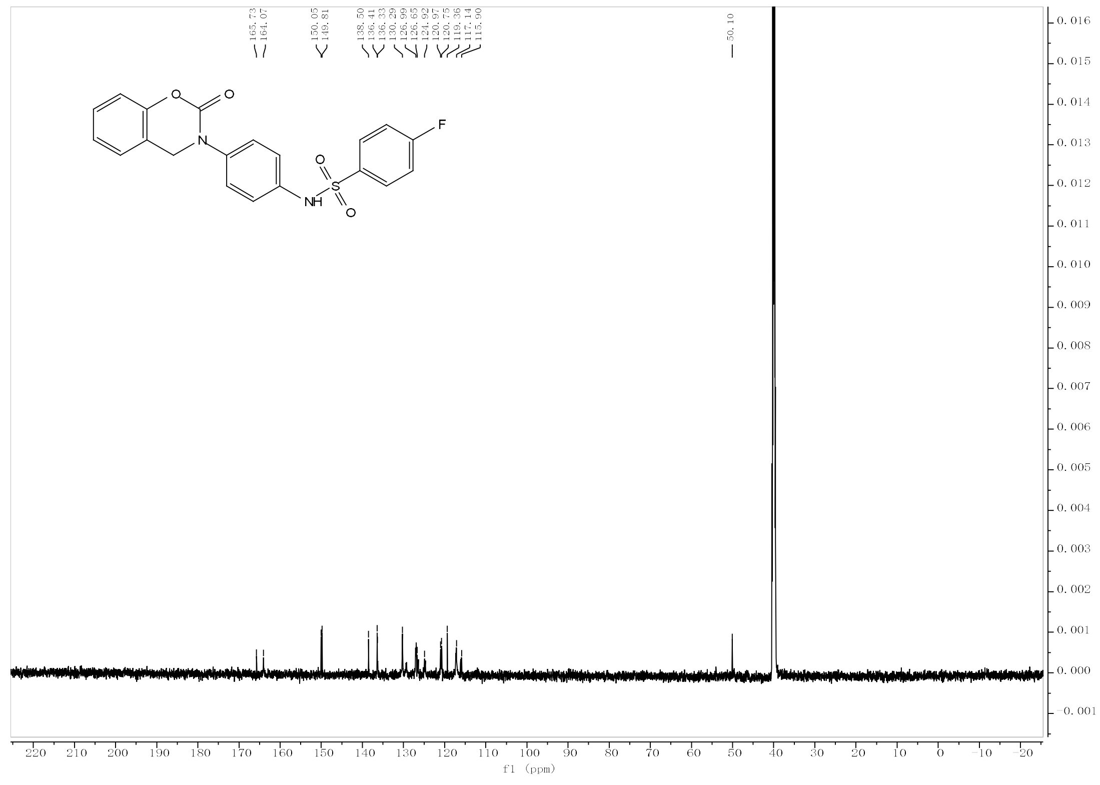

Figure 50 ^{13}C NMR (151 MHz, DMSO- d_6) spectrum of compound L17.

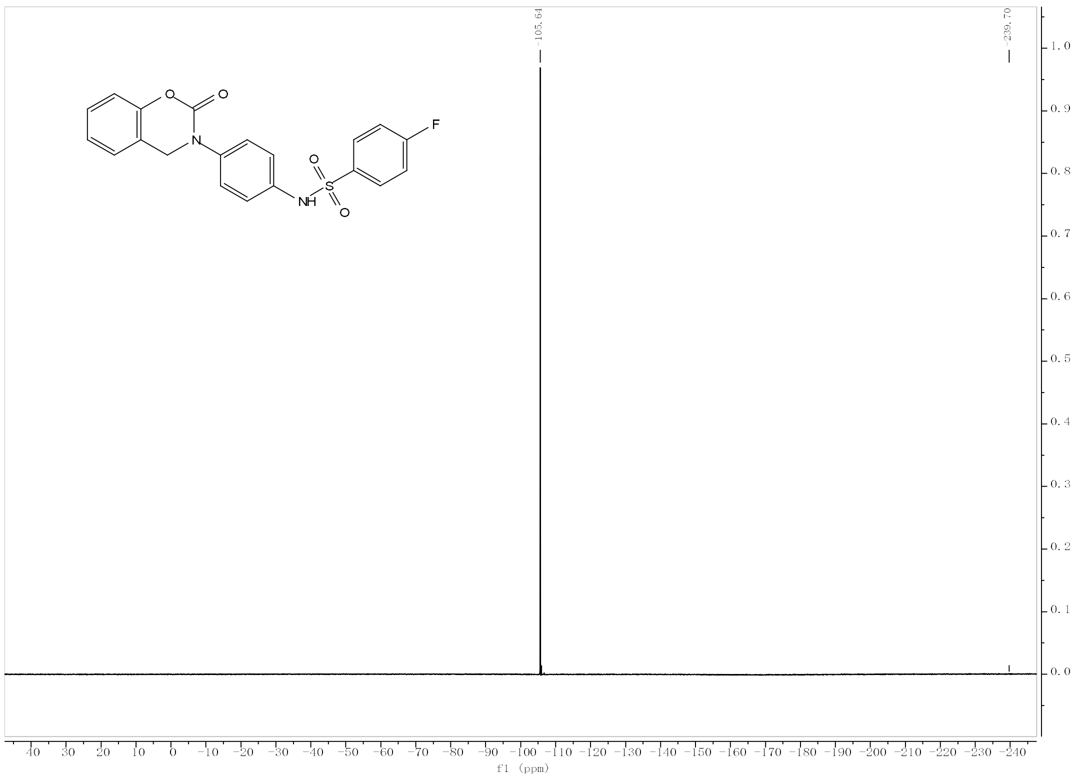

Figure 51 ^{19}F NMR (471 MHz, DMSO-d_6) spectrum of compound L17.

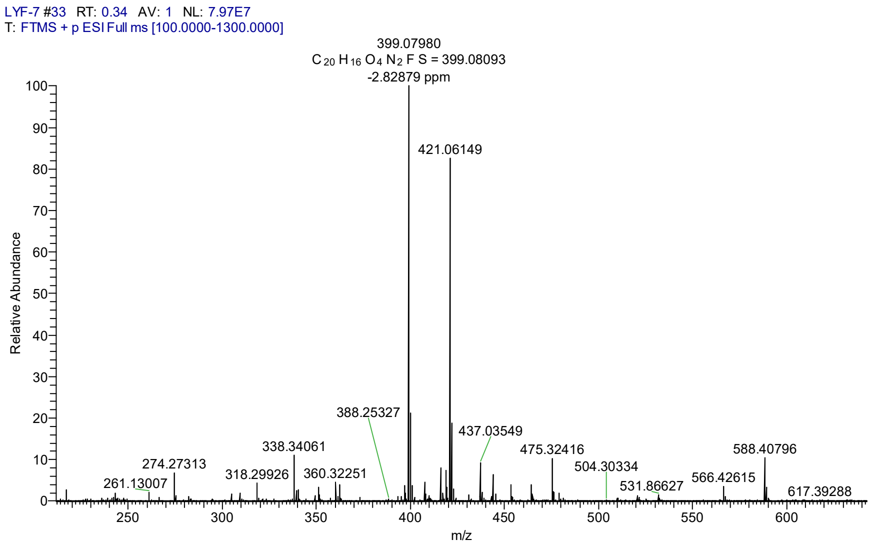

Figure 52. HRMS of compound L17.

Figure 53 ^{1}H NMR (500 MHz, DMSO- d_6) spectrum of compound L18.

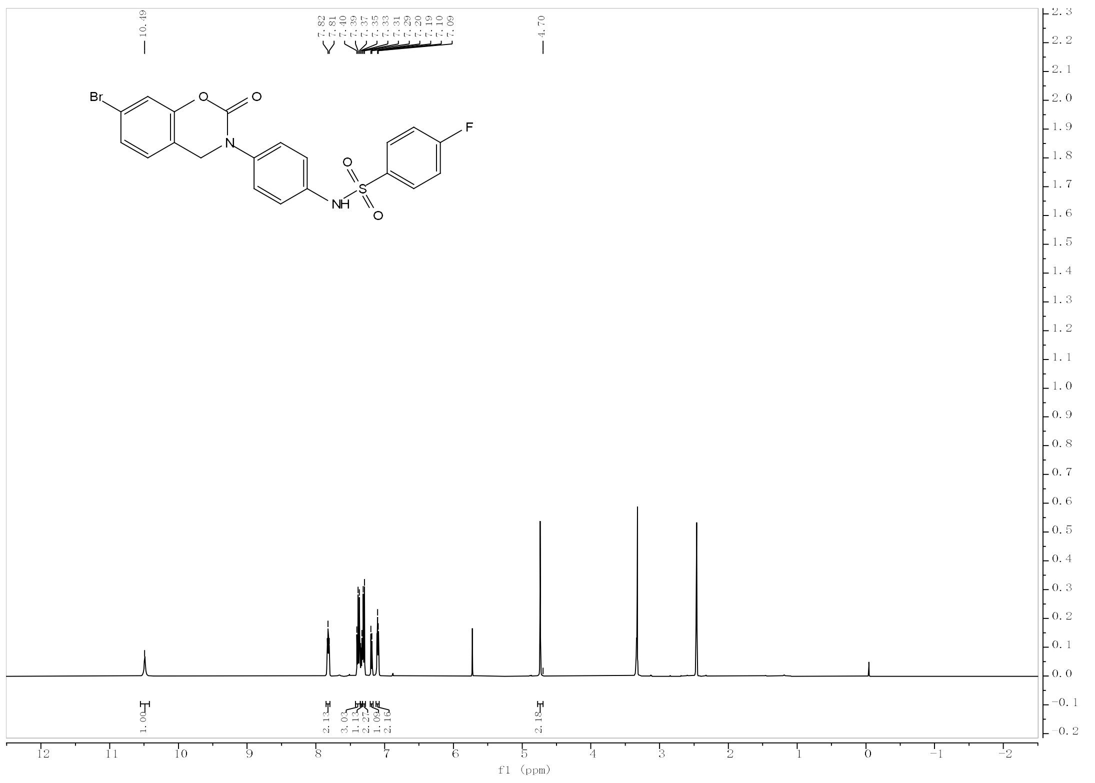

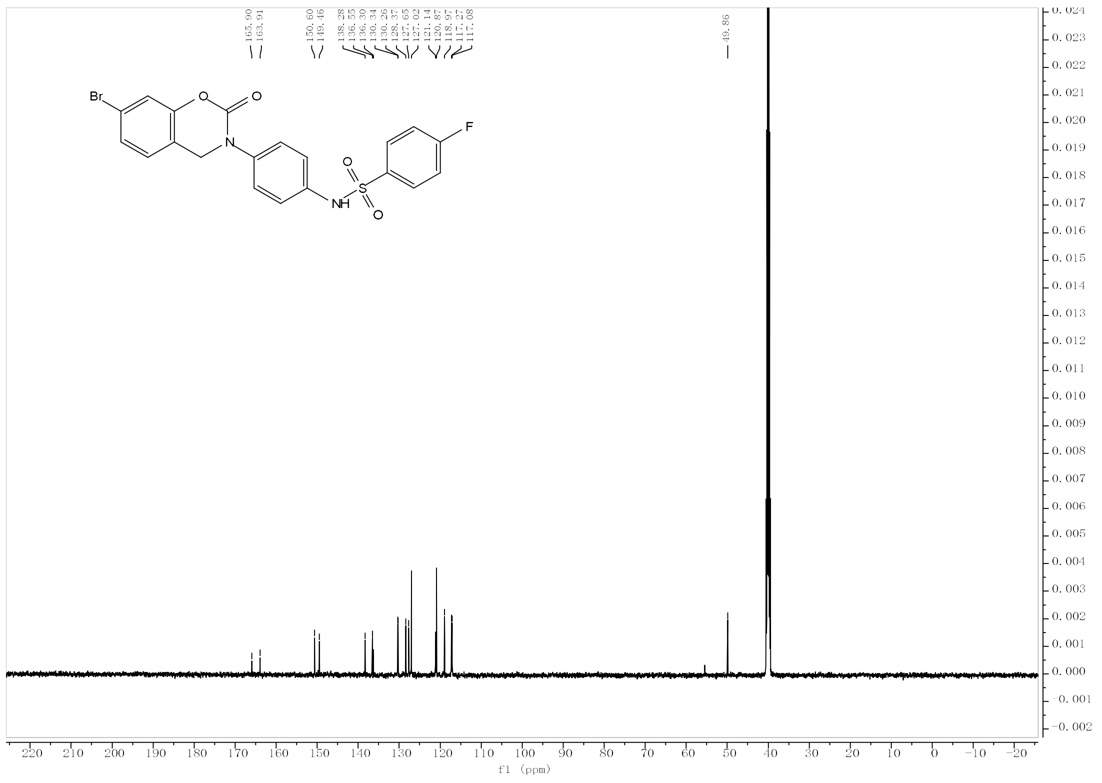

Figure 54 ^{13}C NMR (126 MHz, DMSO- d_6) spectrum of compound L18.

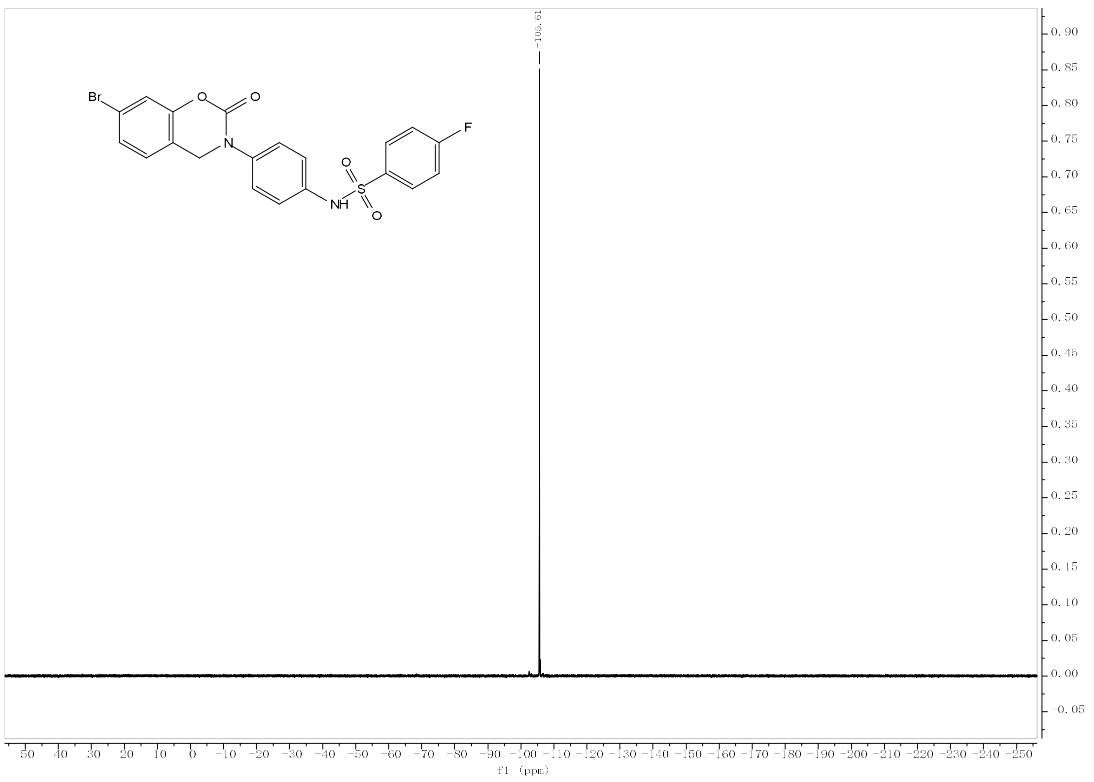

Figure 55 ^{19}F NMR (471 MHz, DMSO- d_6) spectrum of compound L18.

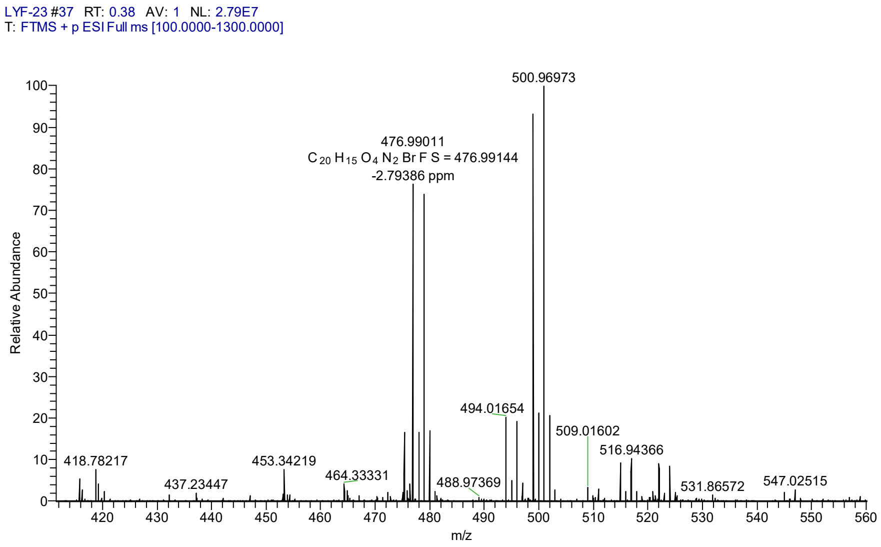

Figure 56. HRMS of compound L18.

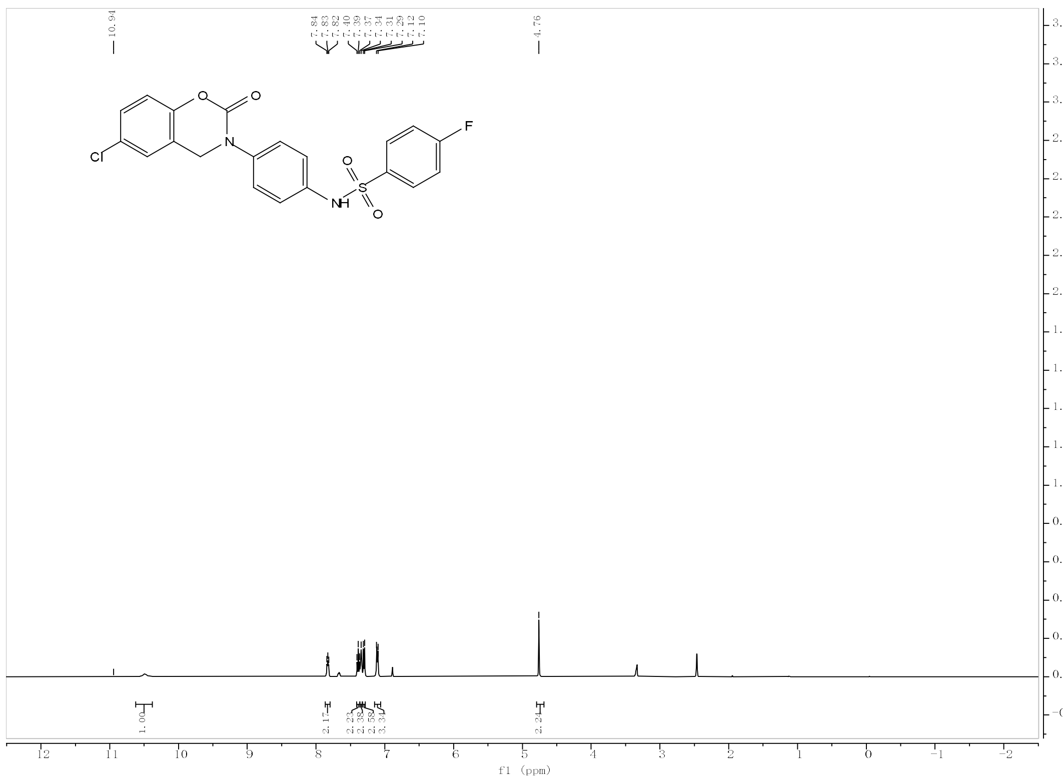

Figure 57 ^{1}H NMR (500 MHz, DMSO- d_6) spectrum of compound L19.

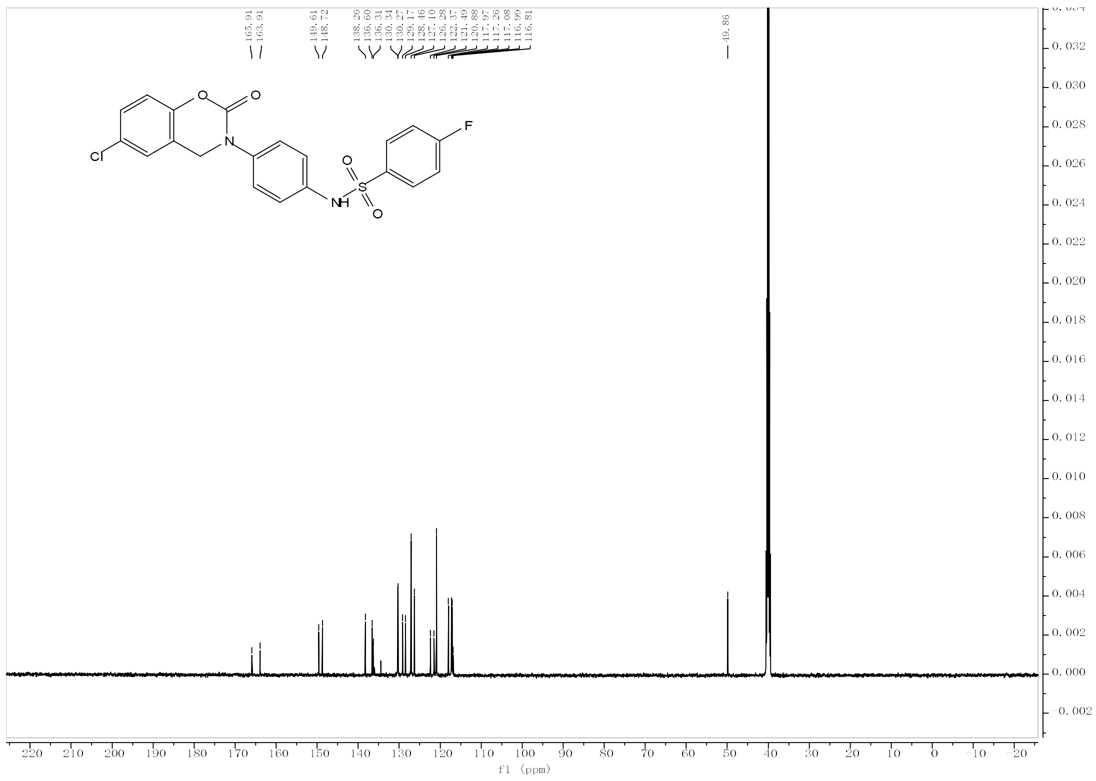

Figure 58 ^{13}C NMR (126 MHz, DMSO- d_6) spectrum of compound L19.

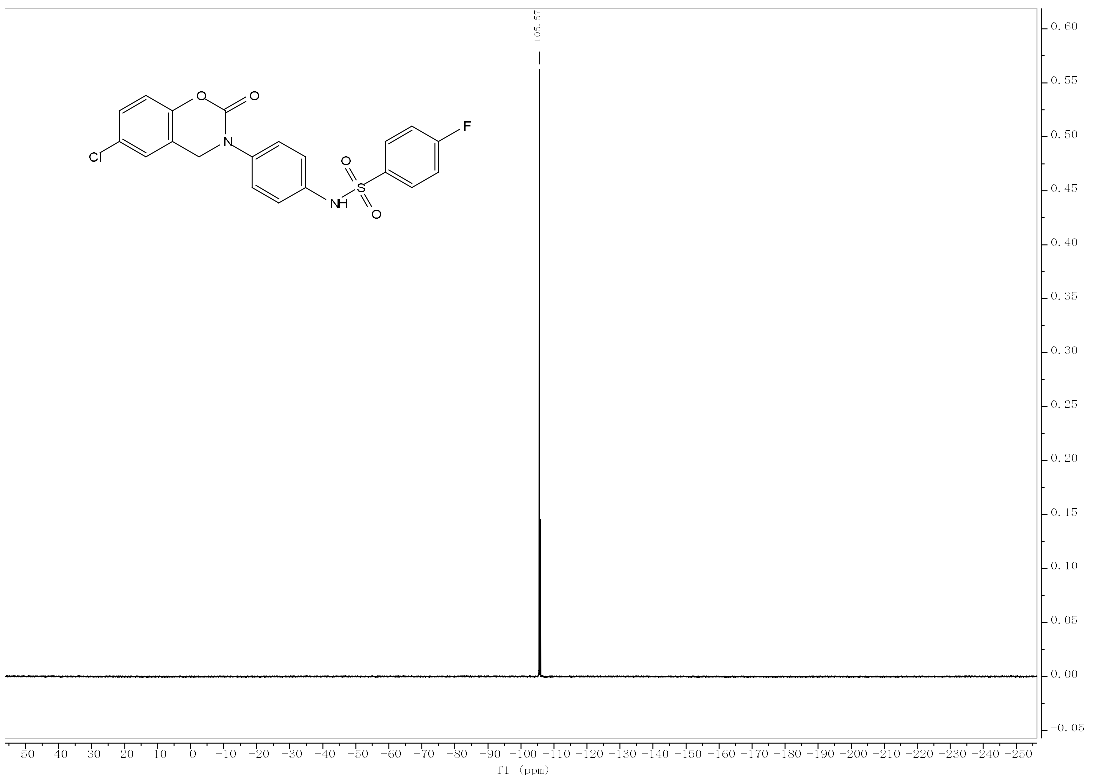

. Figure 59 ^{19}F NMR (471 MHz, DMSO- d_6) spectrum of compound L19.

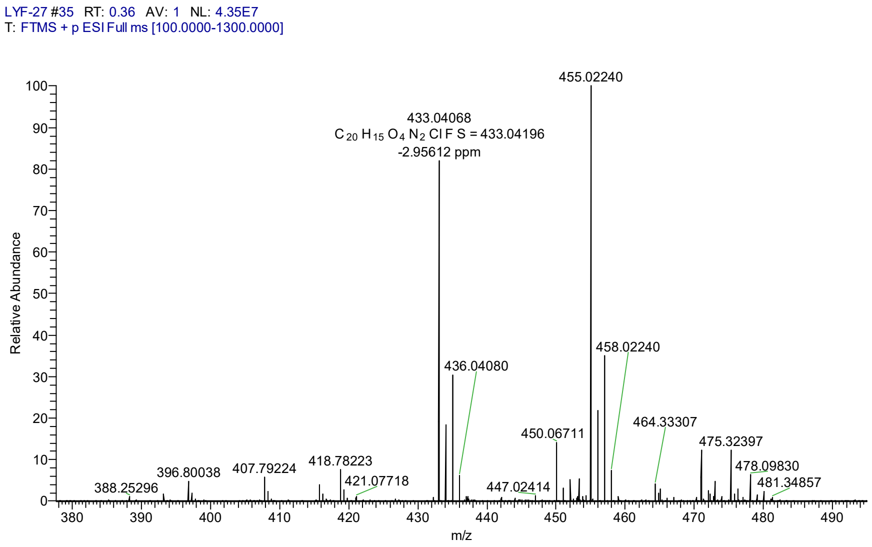

Figure 60. HRMS of compound L19.

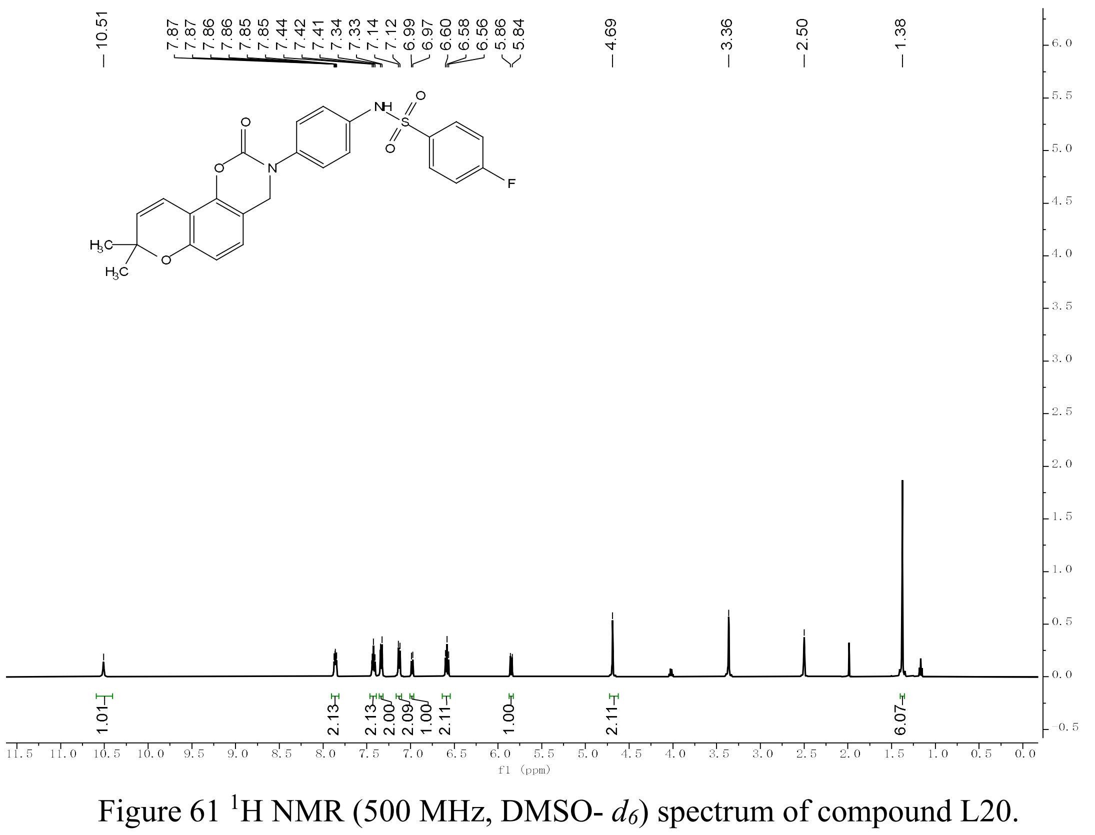

Figure 61 ^{1}H NMR (500 MHz, DMSO- d_6) spectrum of compound L20.

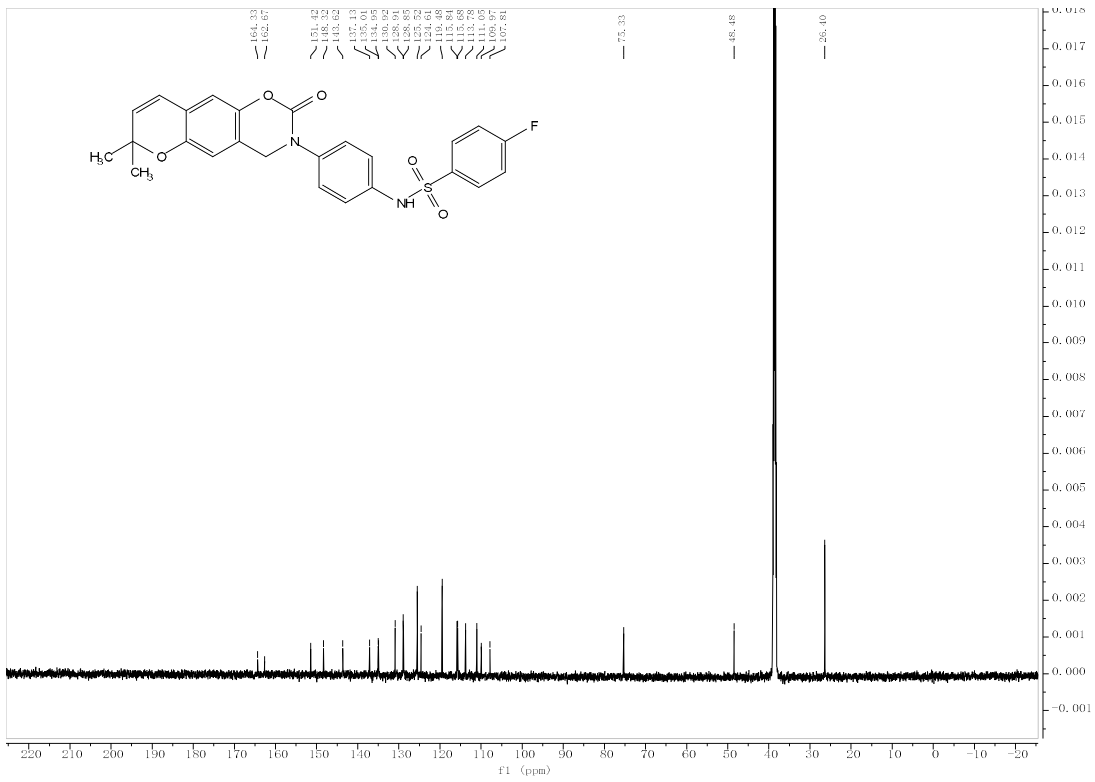

Figure 62 ^{13}C NMR (126 MHz, DMSO- d_6) spectrum of compound L20.

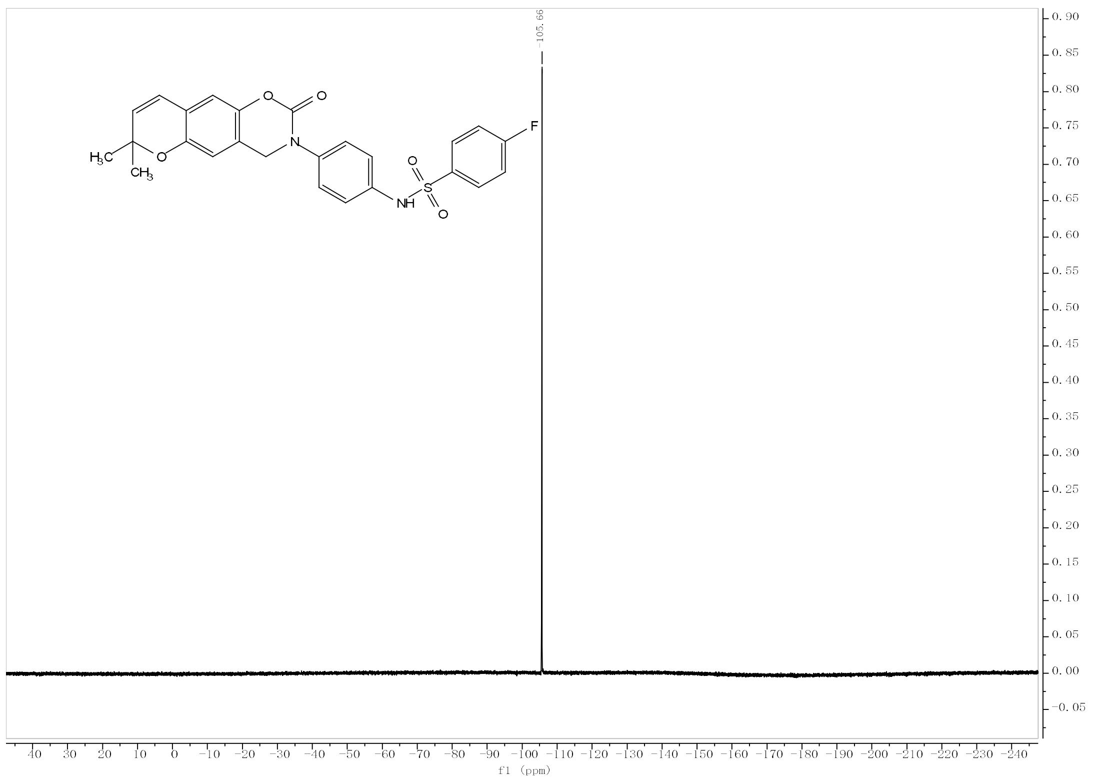

Figure 63 ^{19}F NMR (471 MHz, DMSO-d_6) spectrum of compound L20.

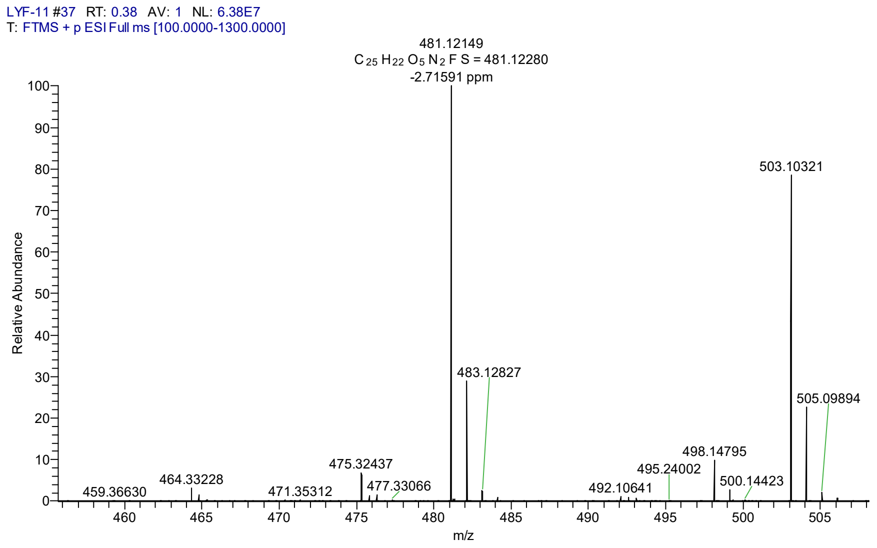

Figure 64. HRMS of compound L20.

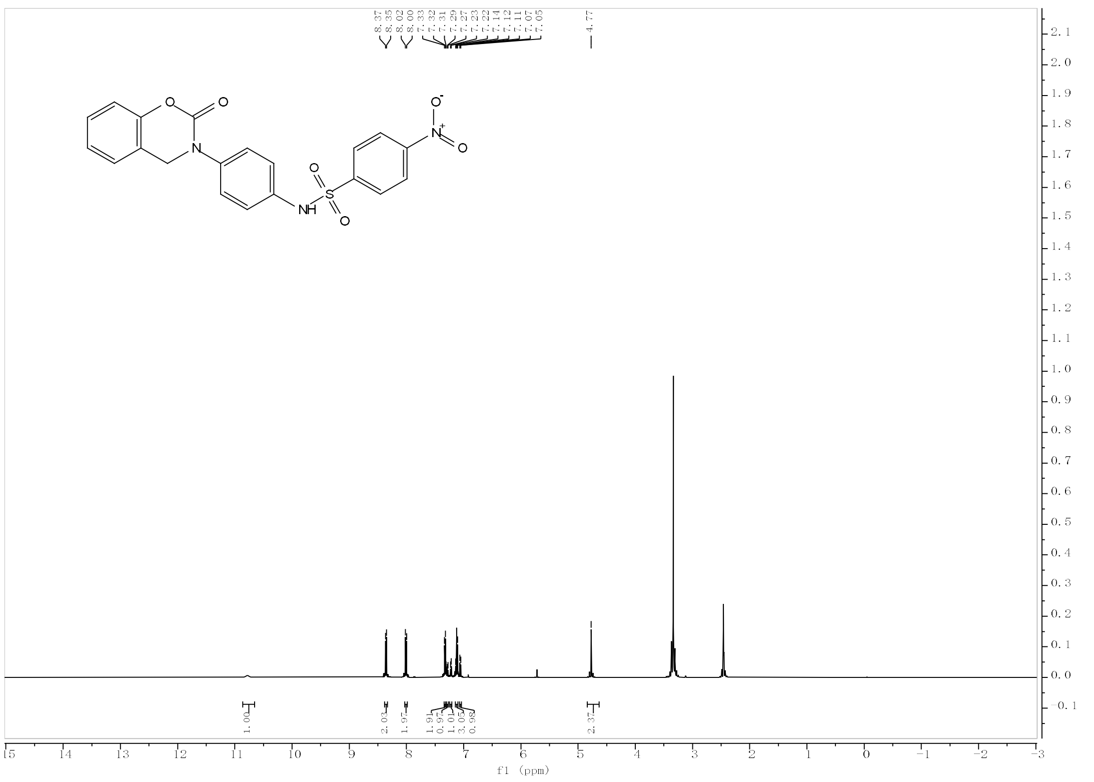

Figure 65. ^{1}H NMR (500 MHz, DMSO- d_6) spectrum of compound L21.

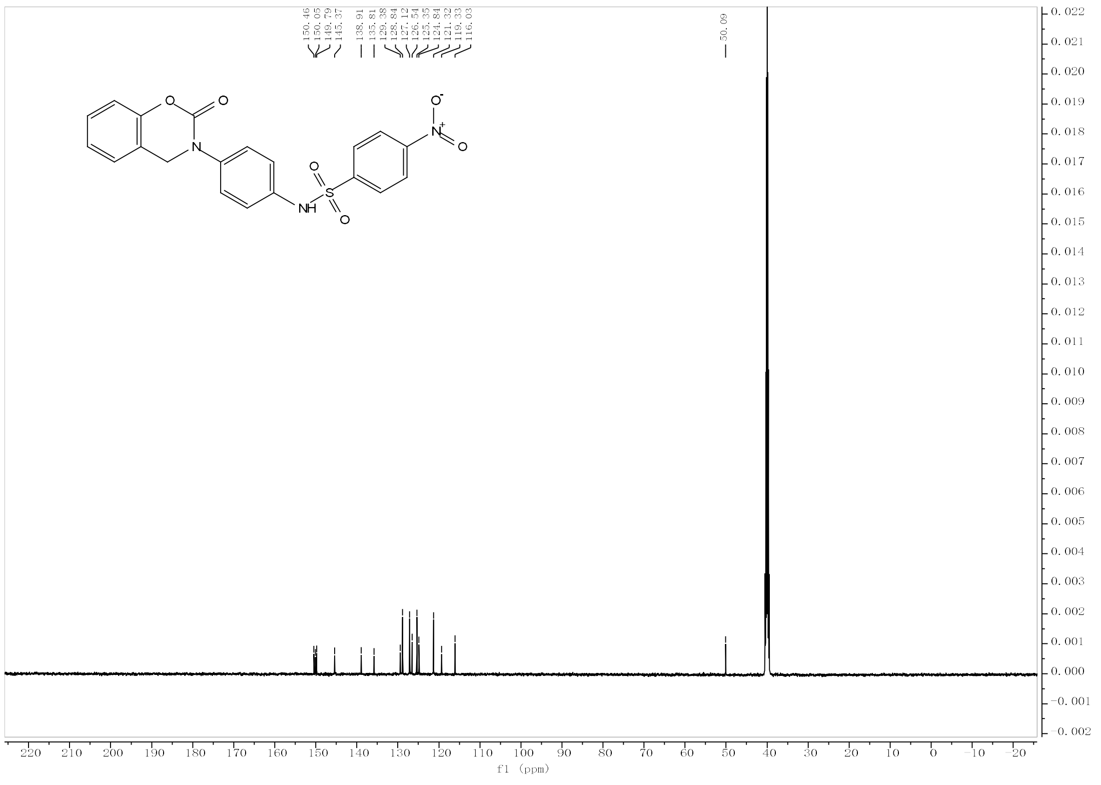

Figure 66 ^{13}C NMR (126 MHz, DMSO- d_6) spectrum of compound L21.

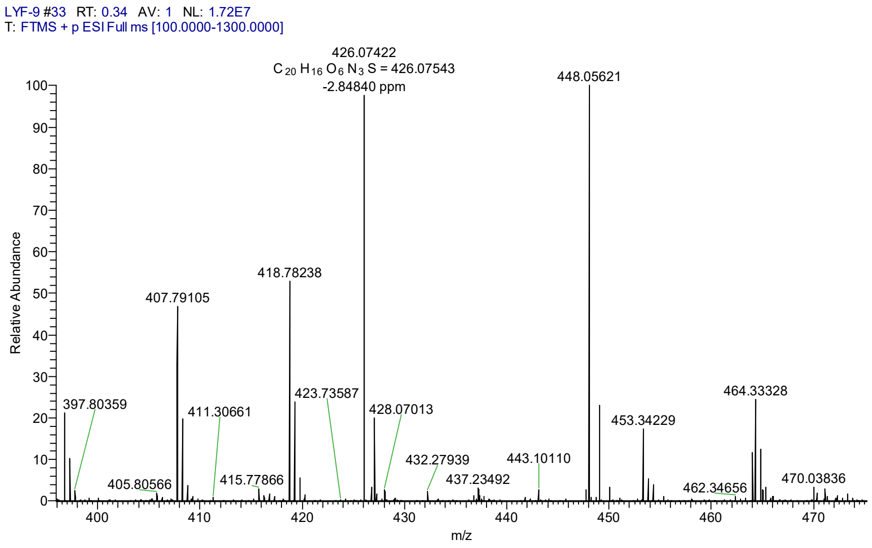

Figure 67. HRMS of compound L21.

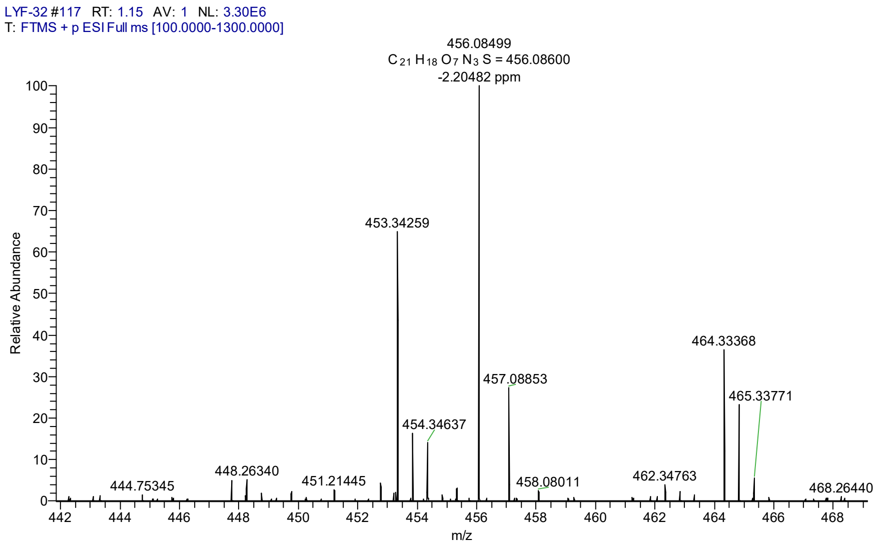

Figure 70. HRMS of compound L22.

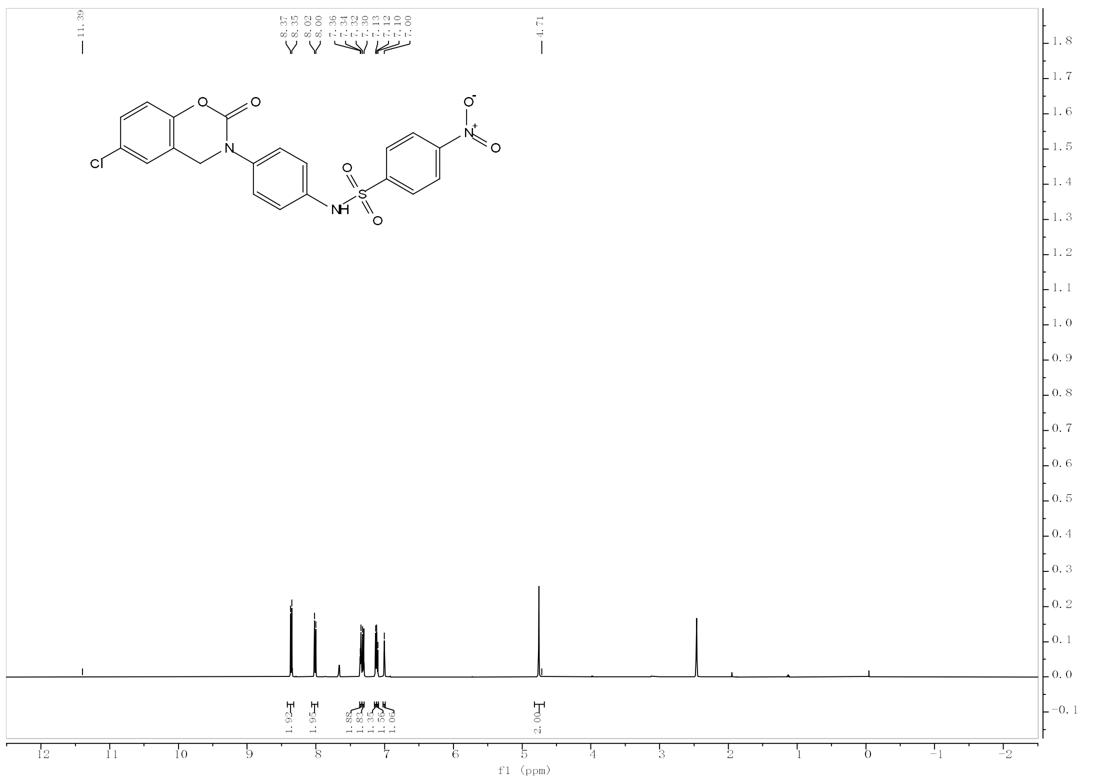

Figure 71 ^{1}H NMR (500 MHz, DMSO- d_6) spectrum of compound L23.

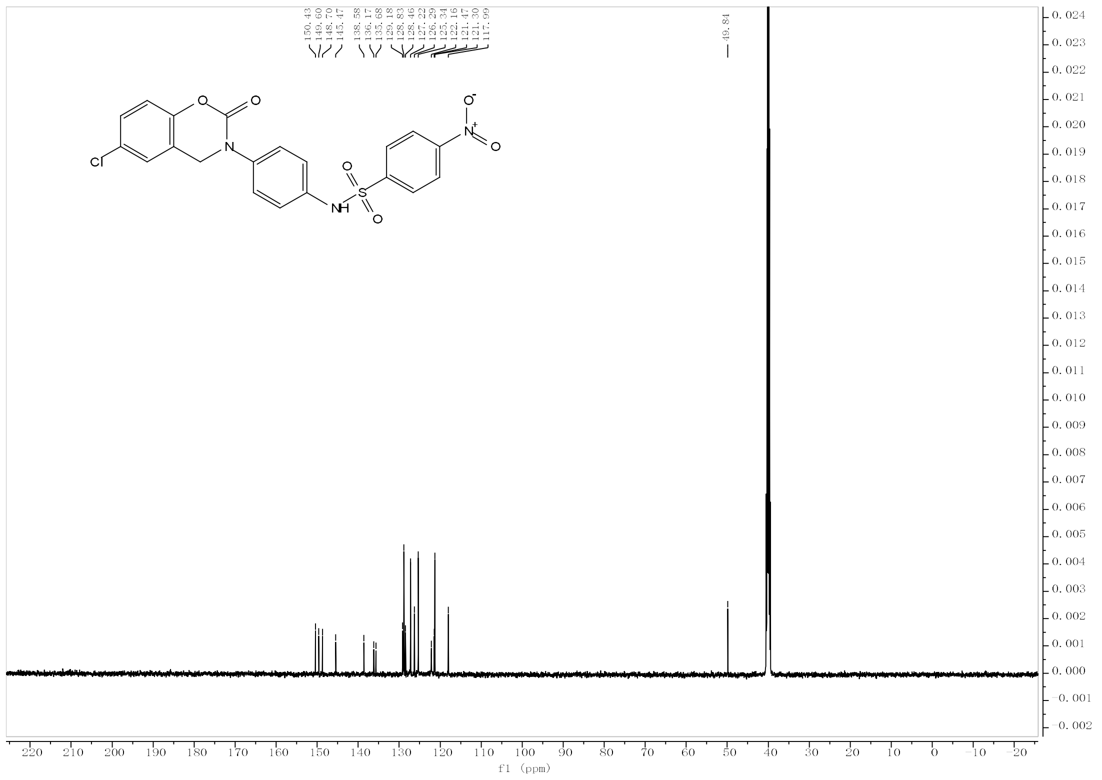

Figure 72 ^{13}C NMR (126 MHz, DMSO- d_6) spectrum of compound L23.

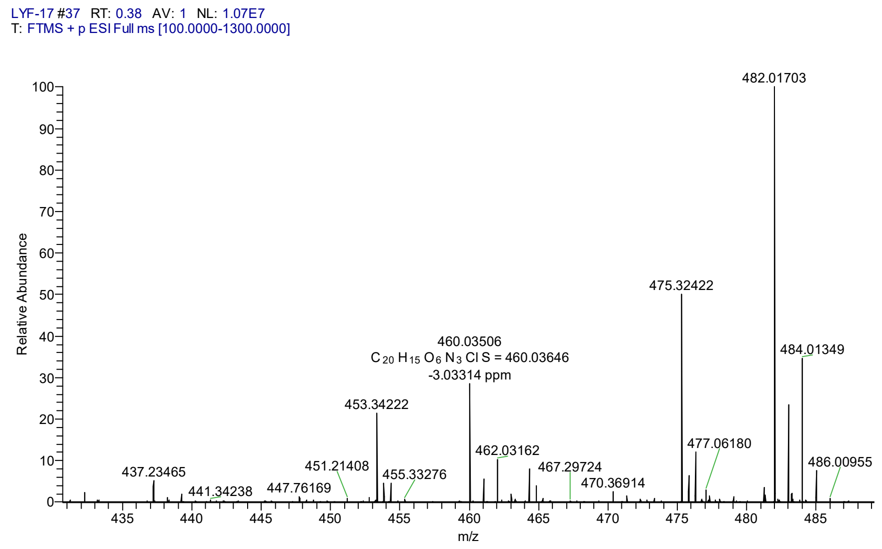

Figure 73. HRMS of compound L23.

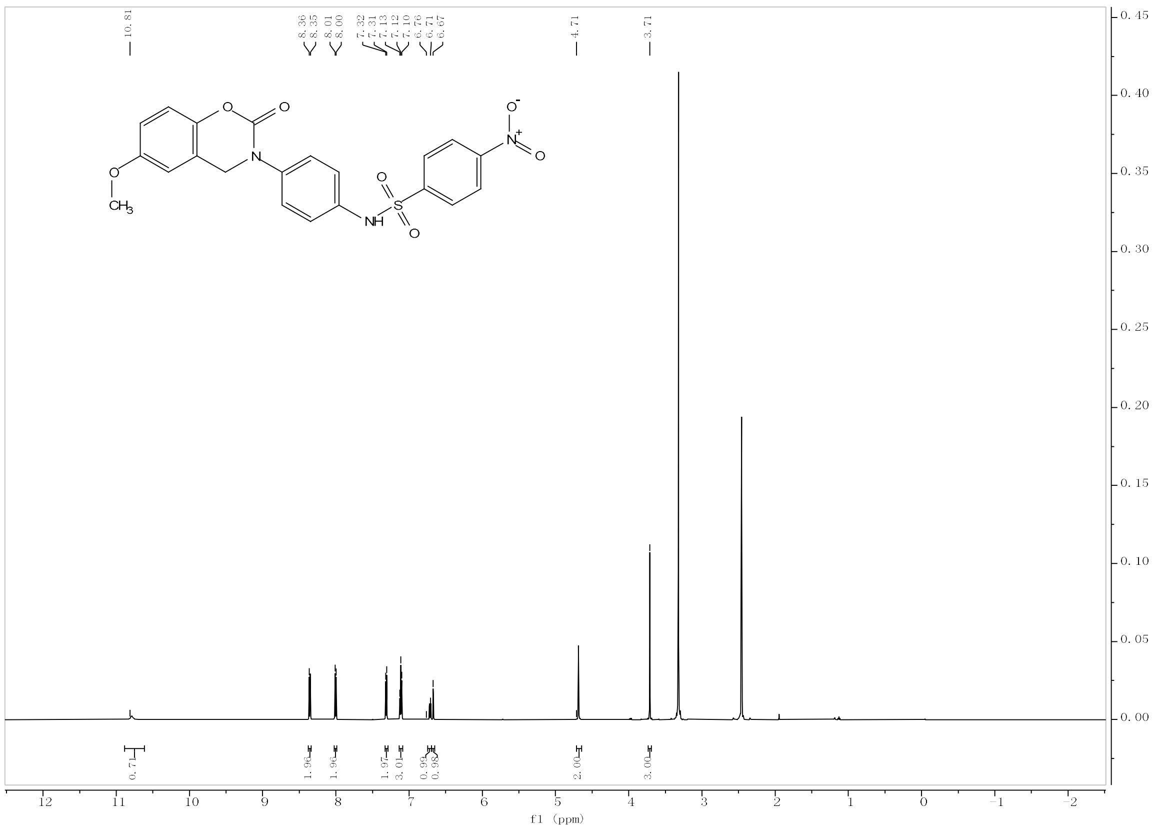

Figure 74 ^{1}H NMR (500 MHz, DMSO- d_6) spectrum of compound L24.

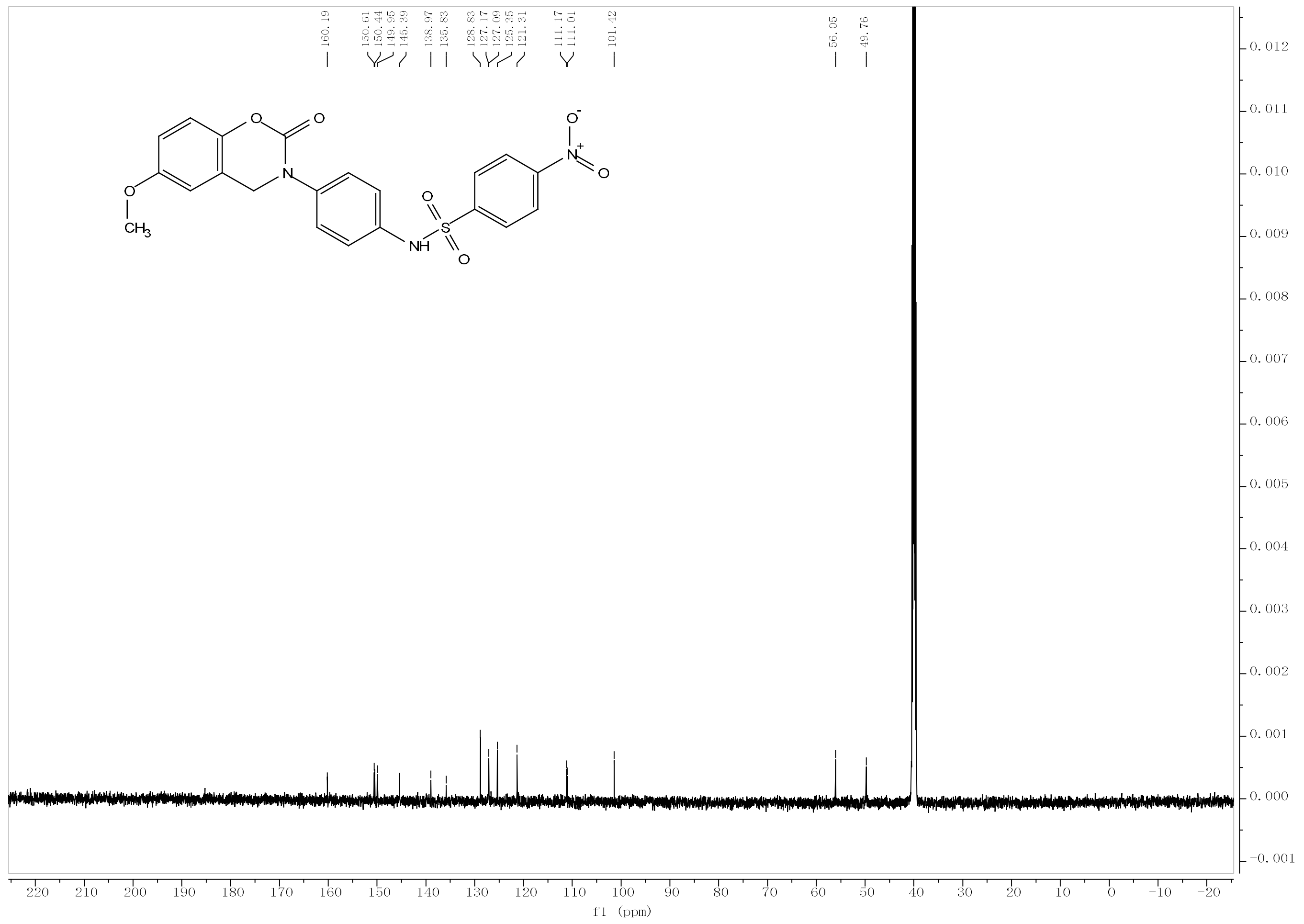

Figure 75 ^{13}C NMR (126 MHz, DMSO- d_6) spectrum of compound L24.

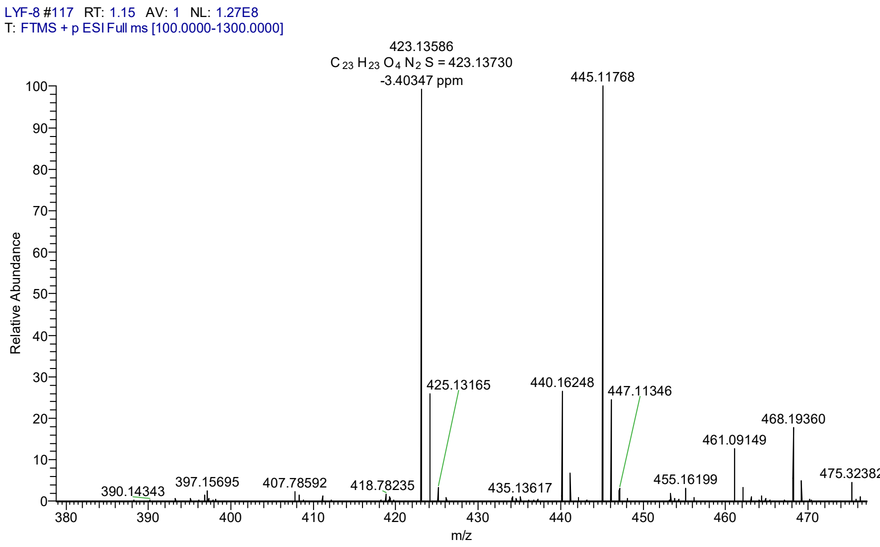

Figure 76. HRMS of compound L24.

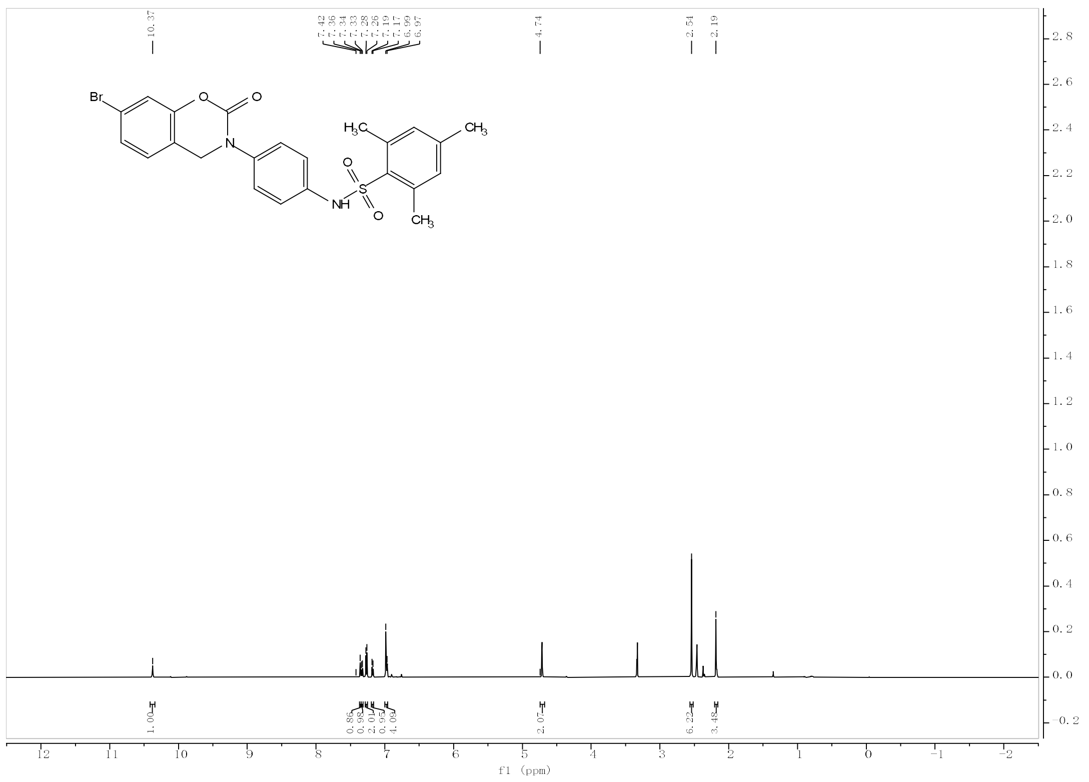

Figure 77 ^{1}H NMR (500 MHz, DMSO- d_6) spectrum of compound L25.

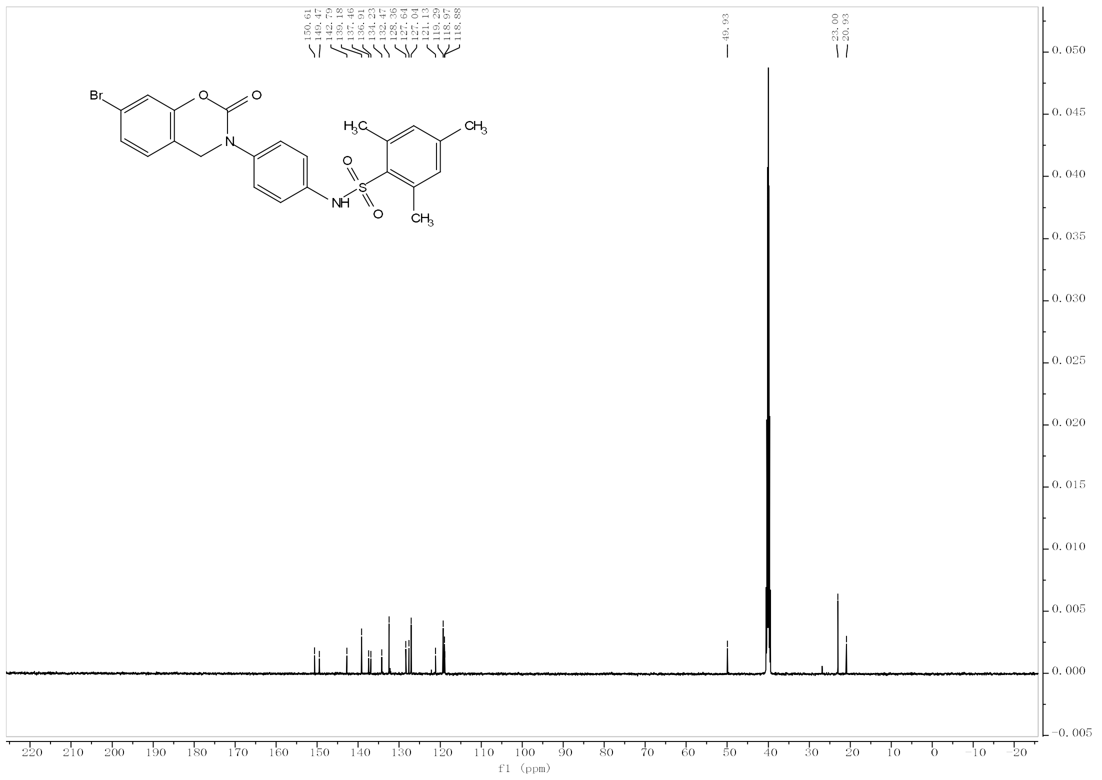

Figure 78 ^{13}C NMR (126 MHz, DMSO- d_6) spectrum of compound L25.

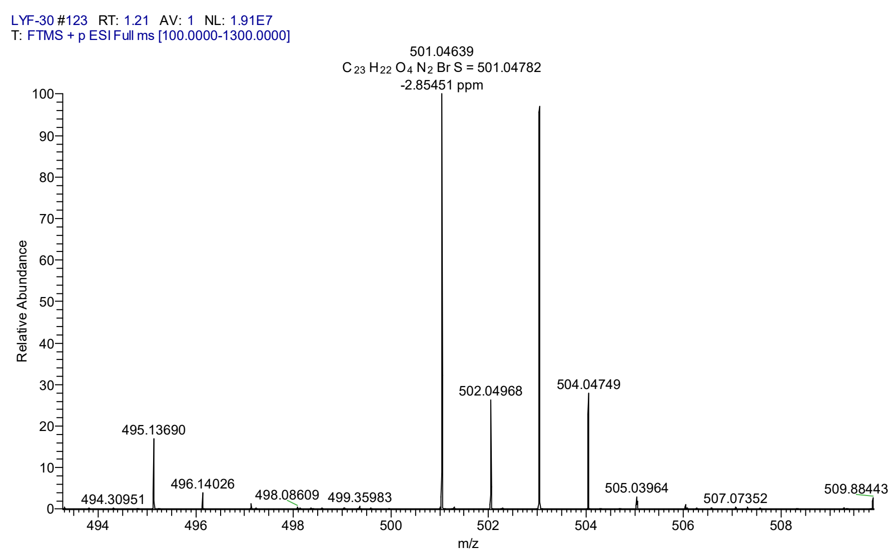

Figure 79. HRMS of compound L25.

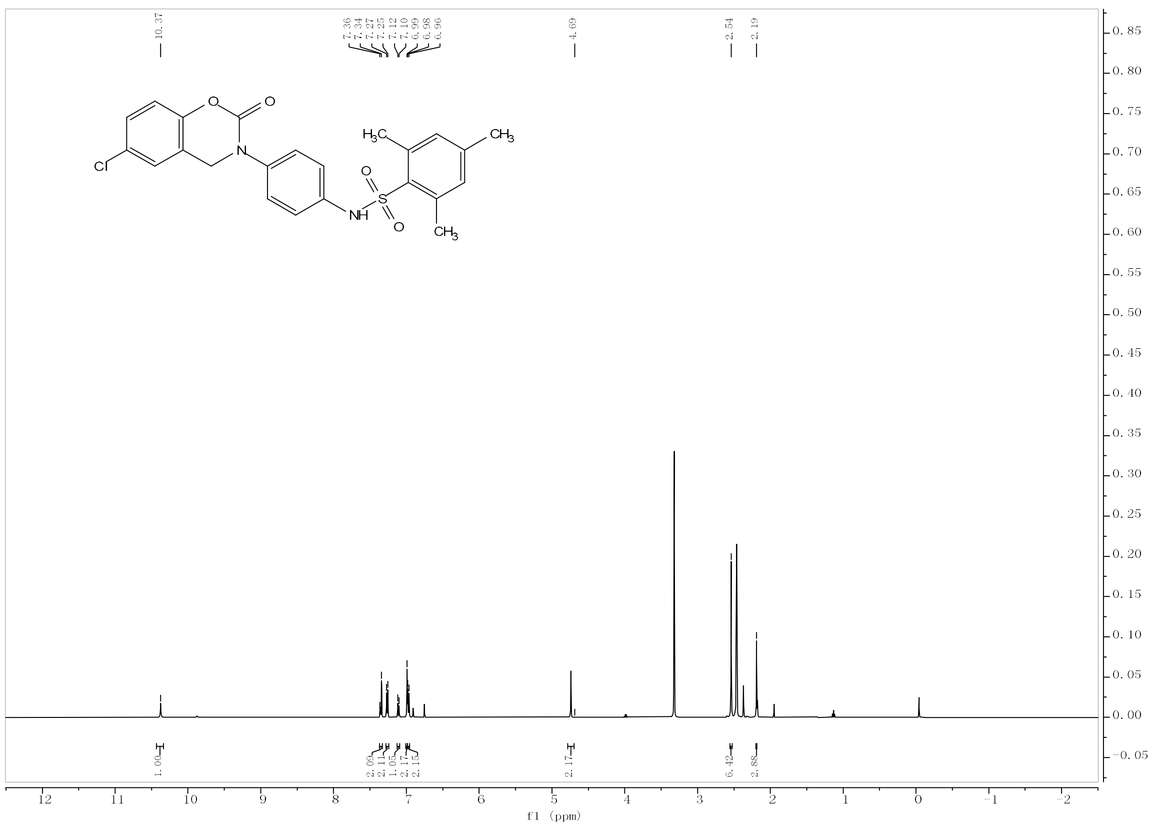

Figure 80 ^{1}H NMR (600 MHz, DMSO- d_6) spectrum of compound L26.

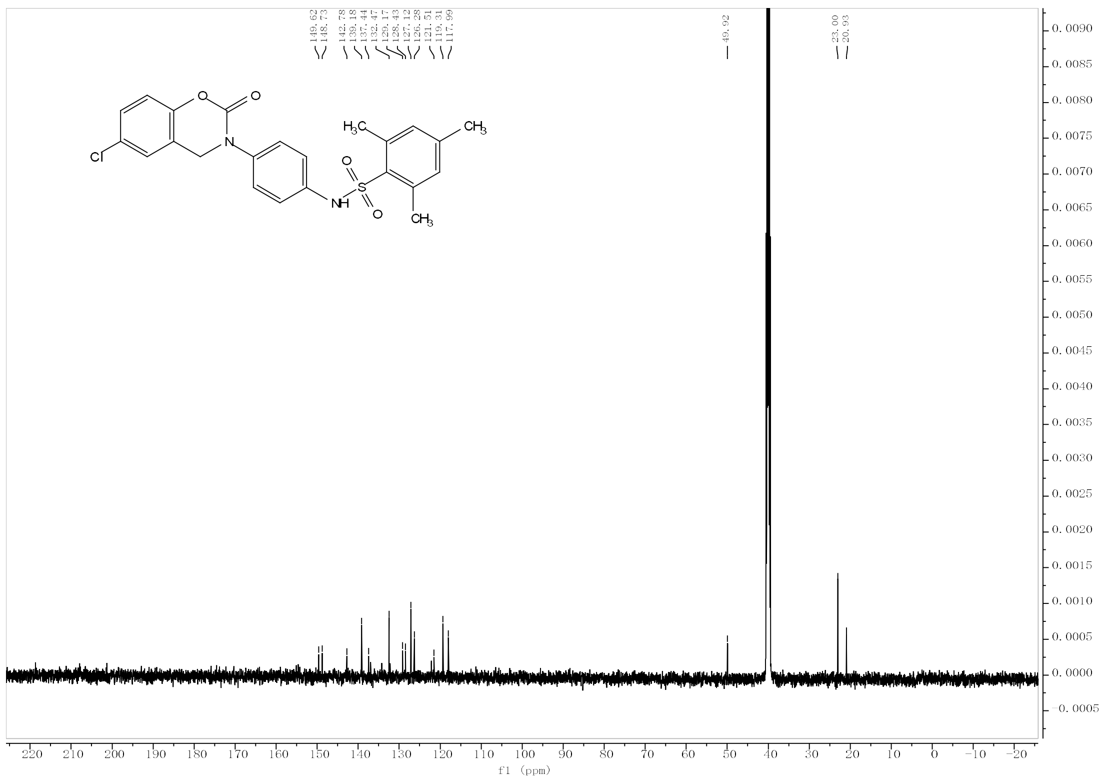

Figure 81 ^{13}C NMR (126 MHz, DMSO- d_6) spectrum of compound L26.

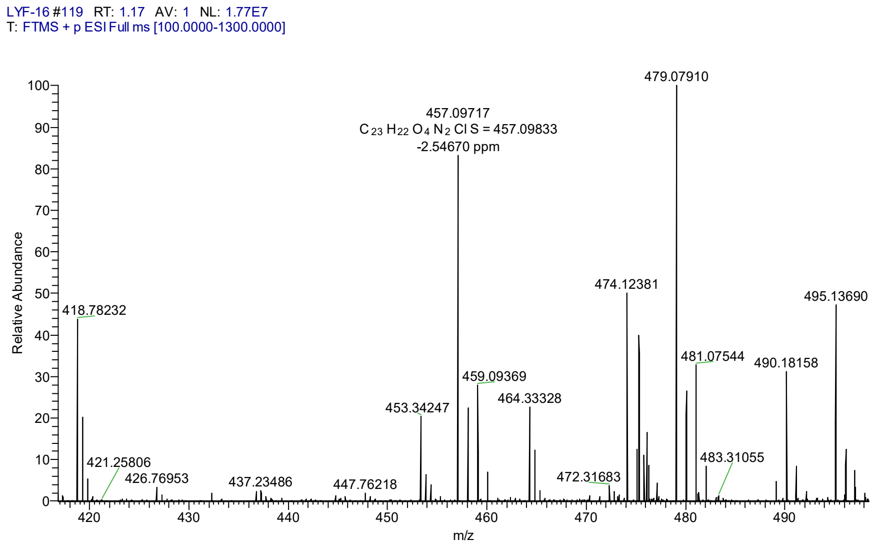

Figure 82. HRMS of compound L26.

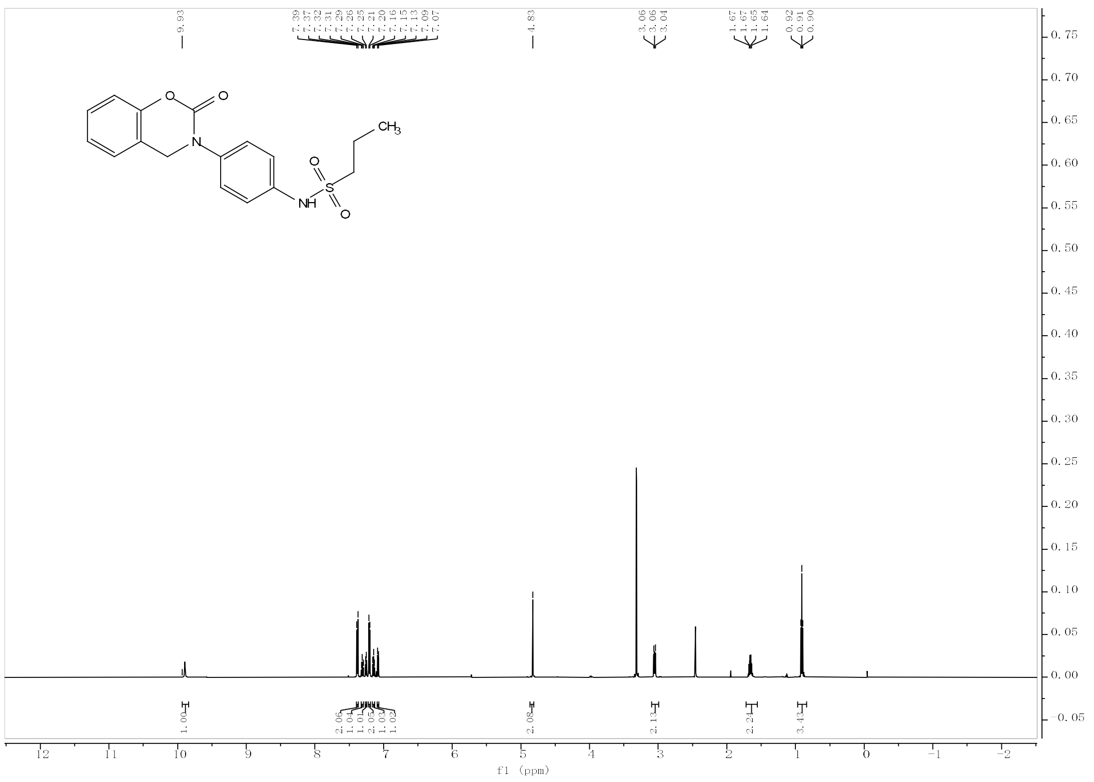

Figure 83. ^{1}H NMR (600 MHz, DMSO- d_6) spectrum of compound L27.

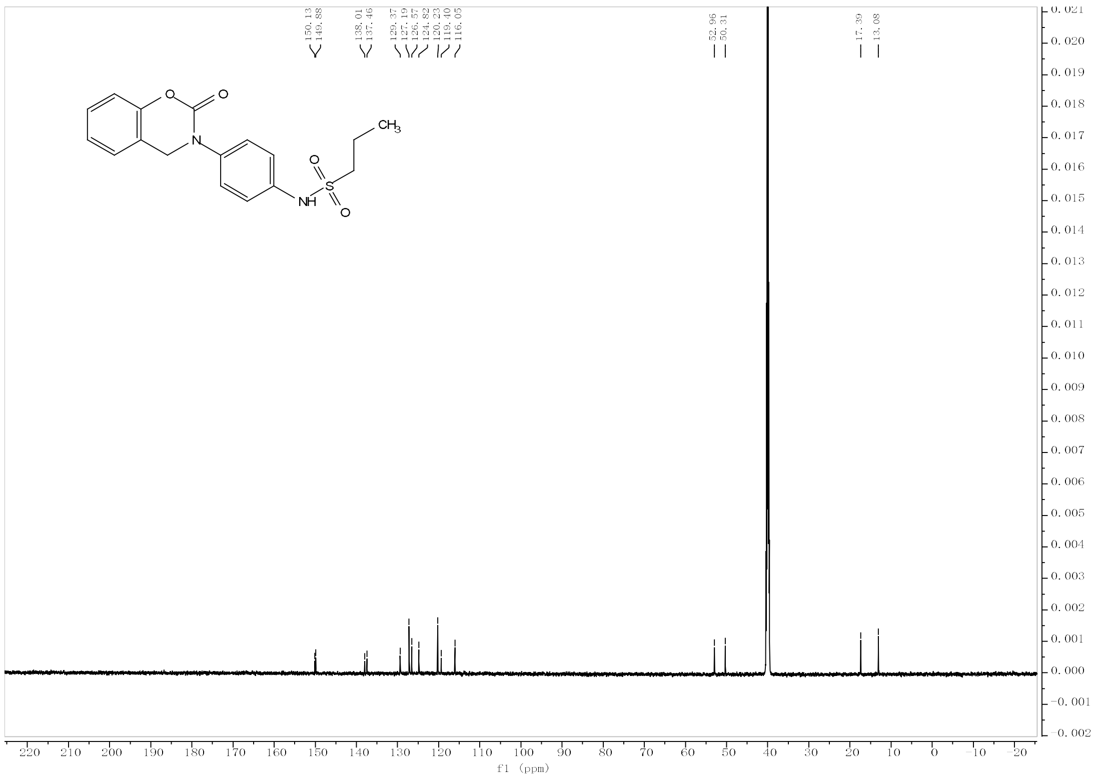

Figure 84 ^{13}C NMR (151 MHz, DMSO- d_6) spectrum of compound L27.

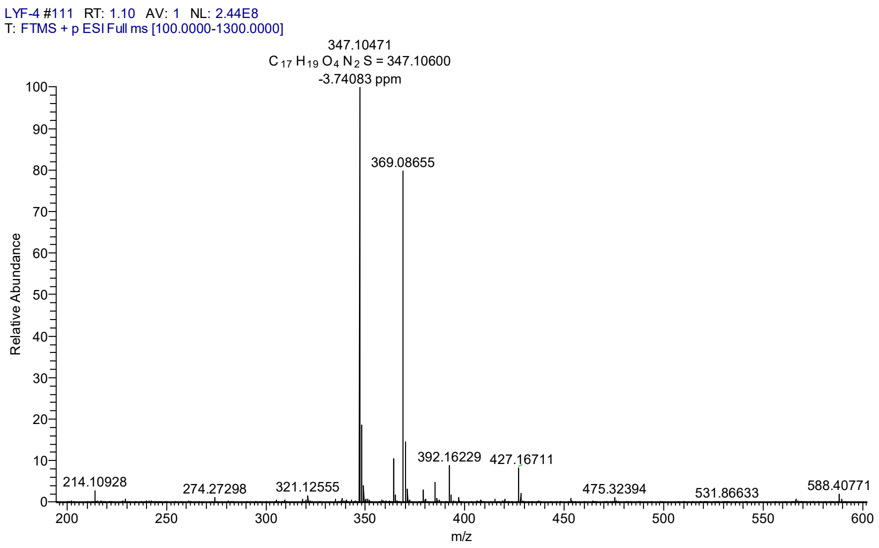

Figure 85. HRMS of compound L27.

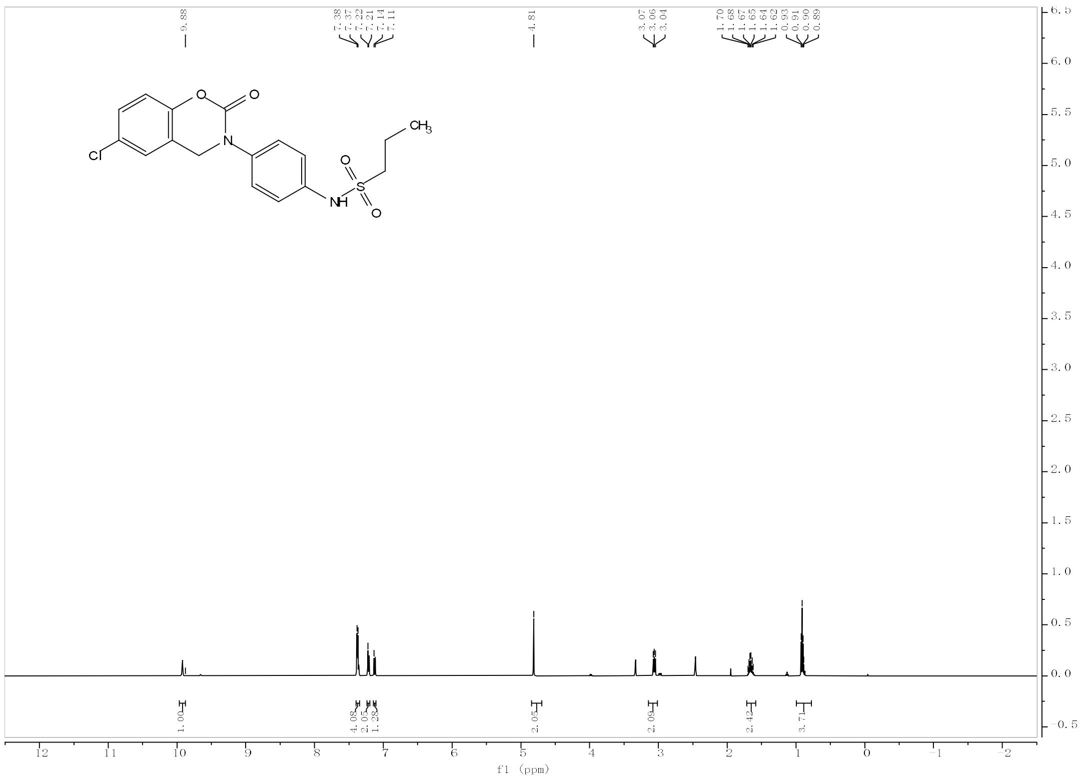

Figure 86 ^{1}H NMR (500 MHz, DMSO- d_6) spectrum of compound L28.

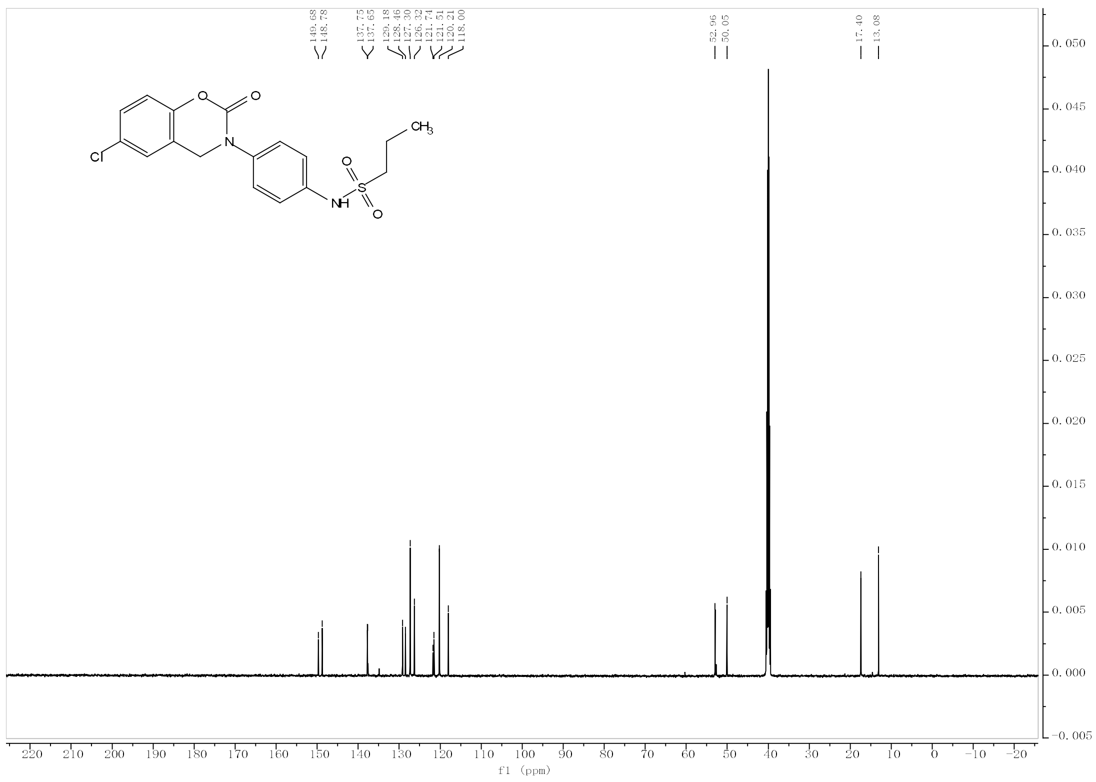

Figure 87 ^{13}C NMR (126 MHz, DMSO- d_6) spectrum of compound L28.

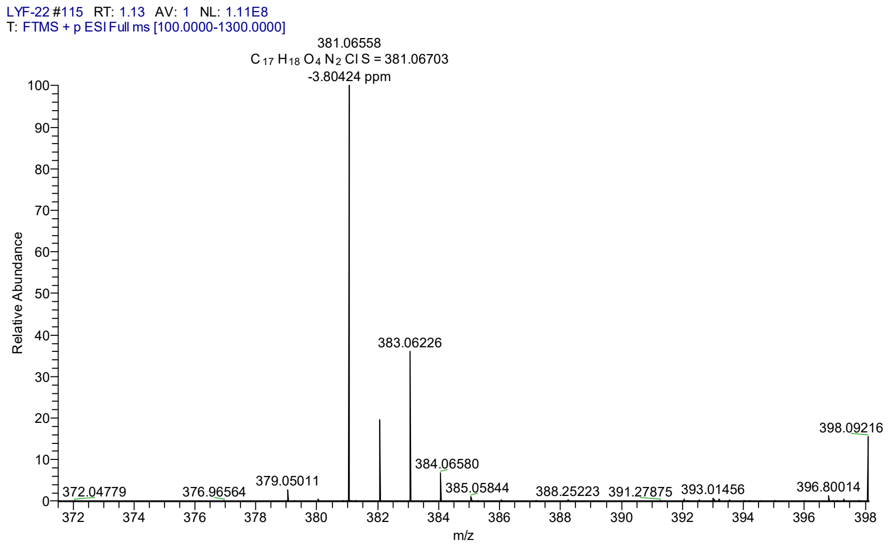

Figure 88. HRMS of compound L28.

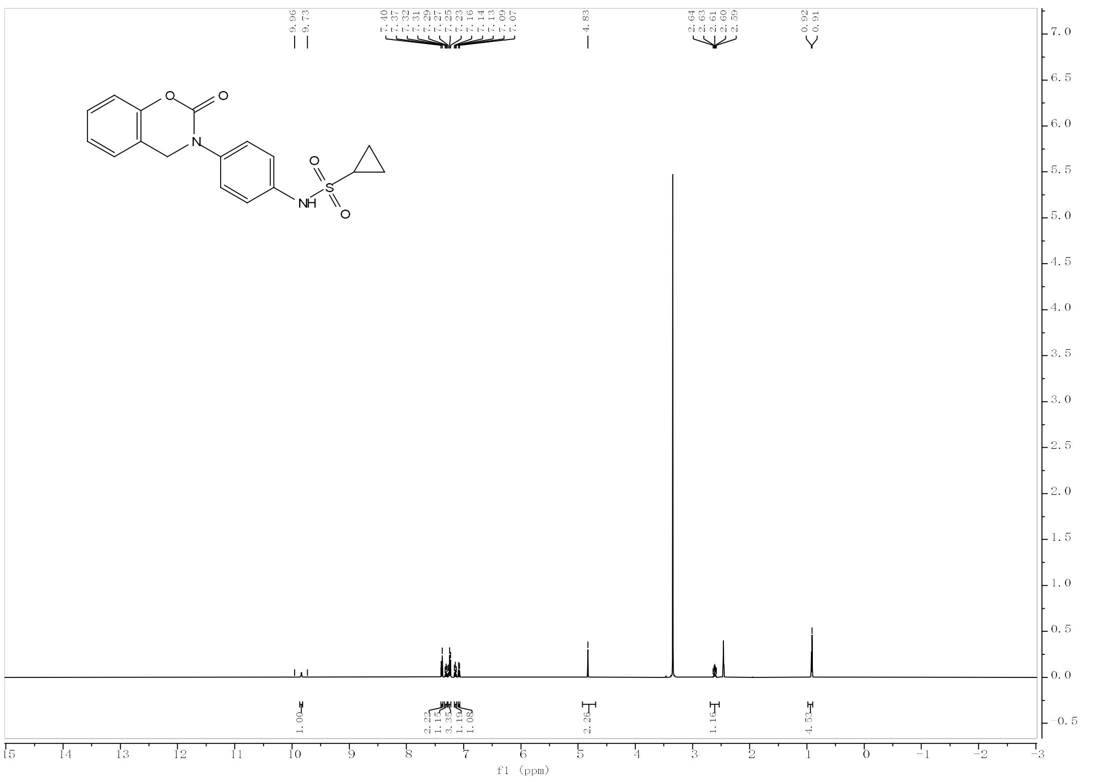

Figure 89 ^{1}H NMR (500 MHz, DMSO- d_6) spectrum of compound L29.

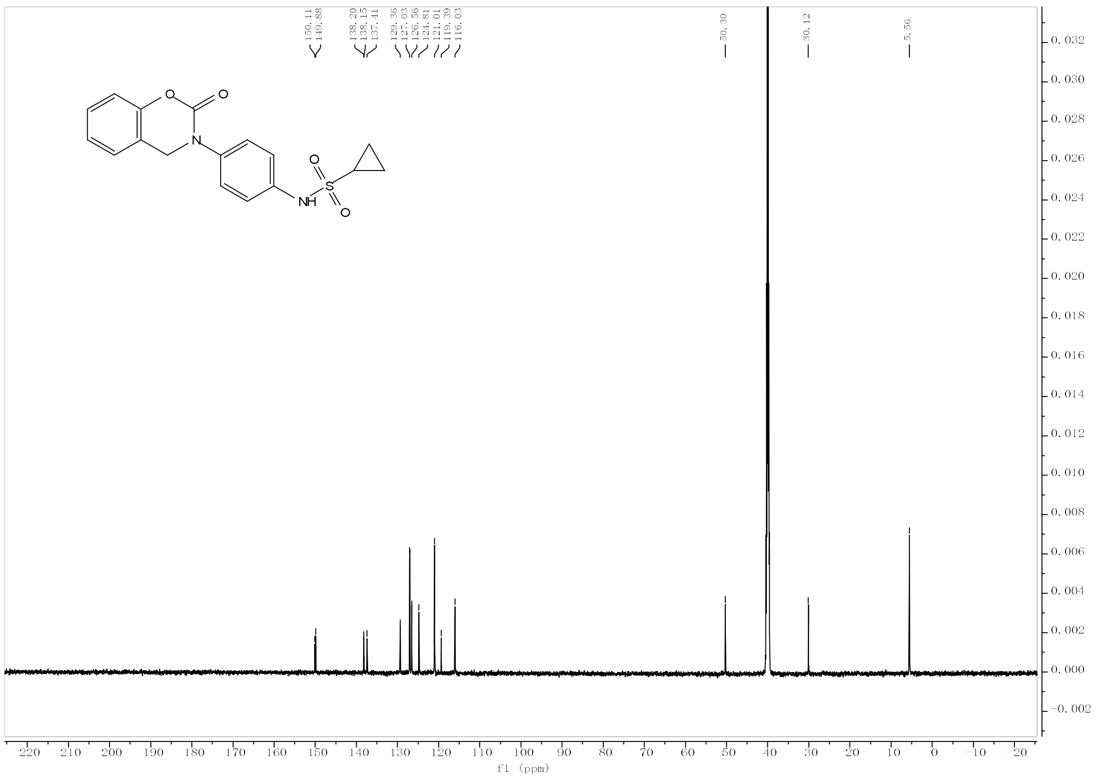

Figure 90 ^{13}C NMR (151 MHz, DMSO- d_6) spectrum of compound L29

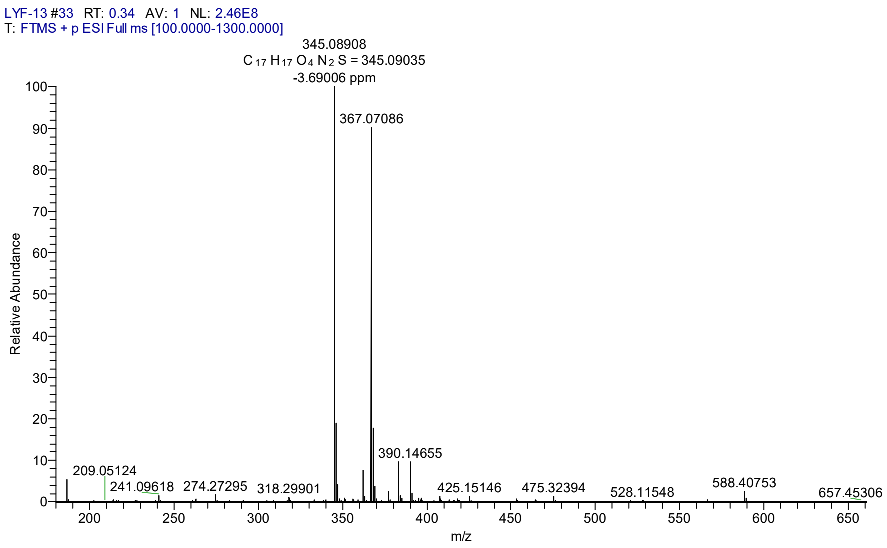

Figure 91. HRMS of compound L29.

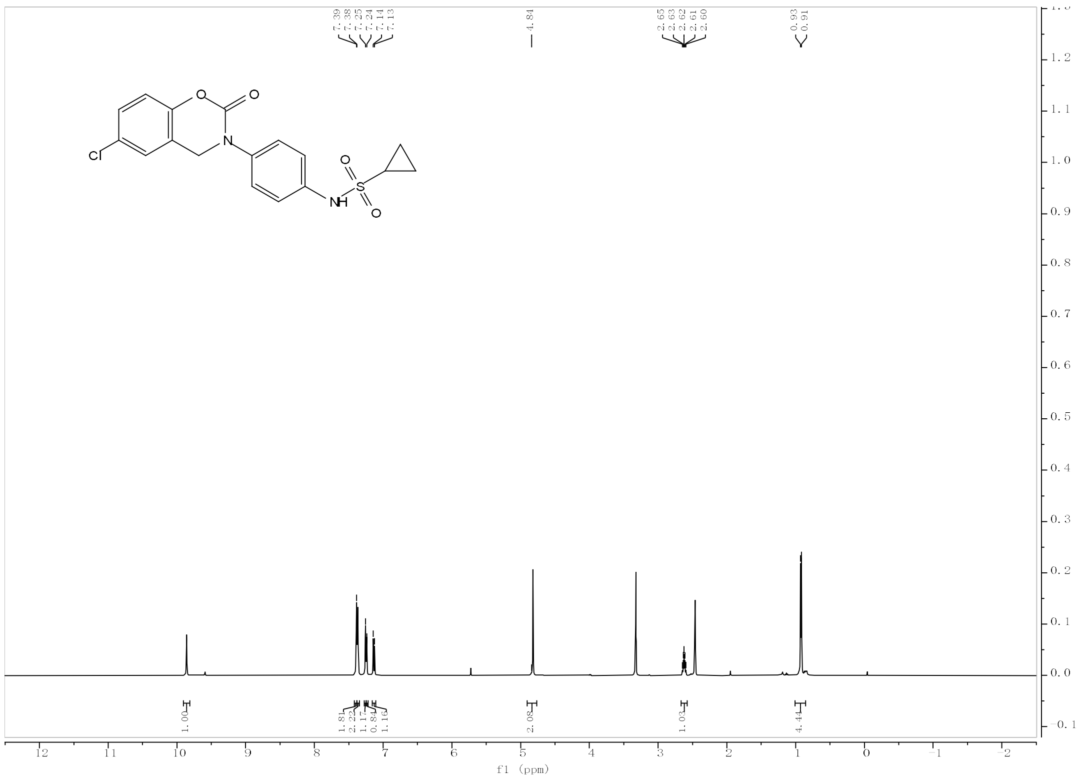

Figure 92 ^{1}H NMR (500 MHz, DMSO- d_6) spectrum of compound L30.

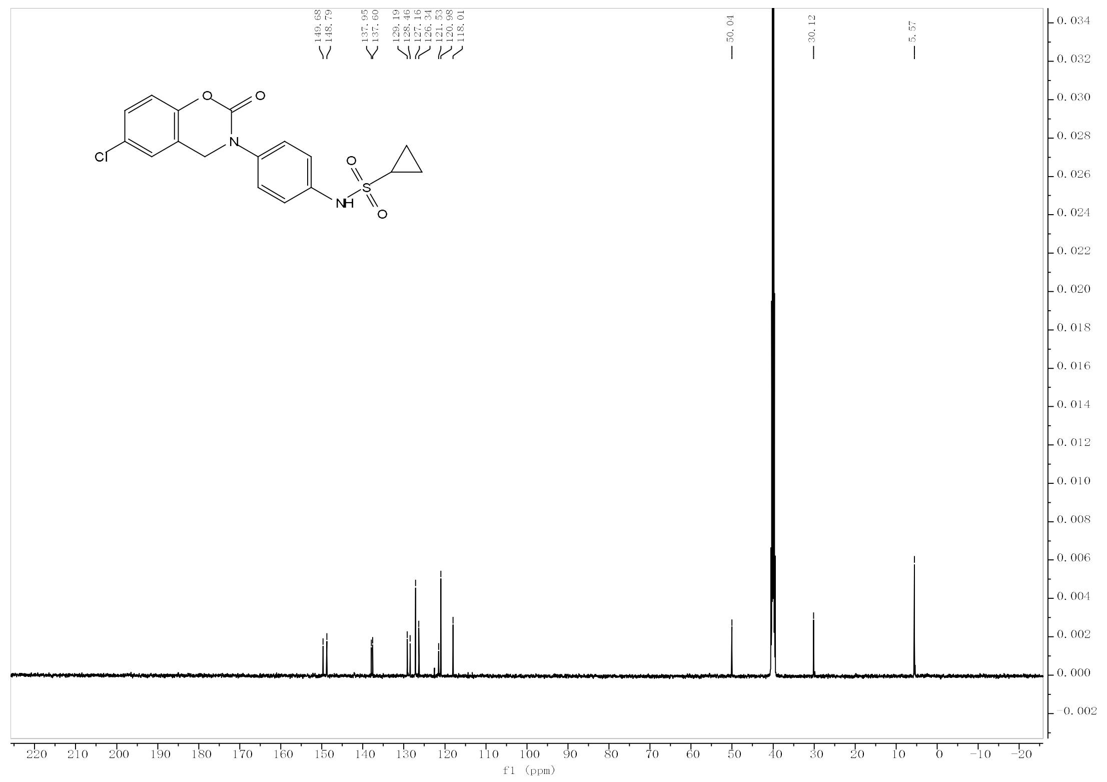

Figure 93 ^{13}C NMR (126 MHz, DMSO- d_6) spectrum of compound L30.

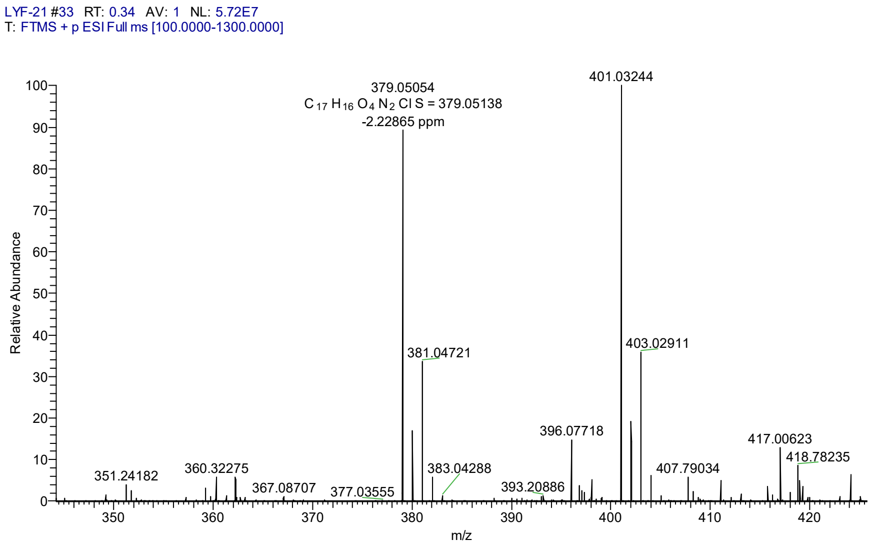

Figure 94. HRMS of compound L30.

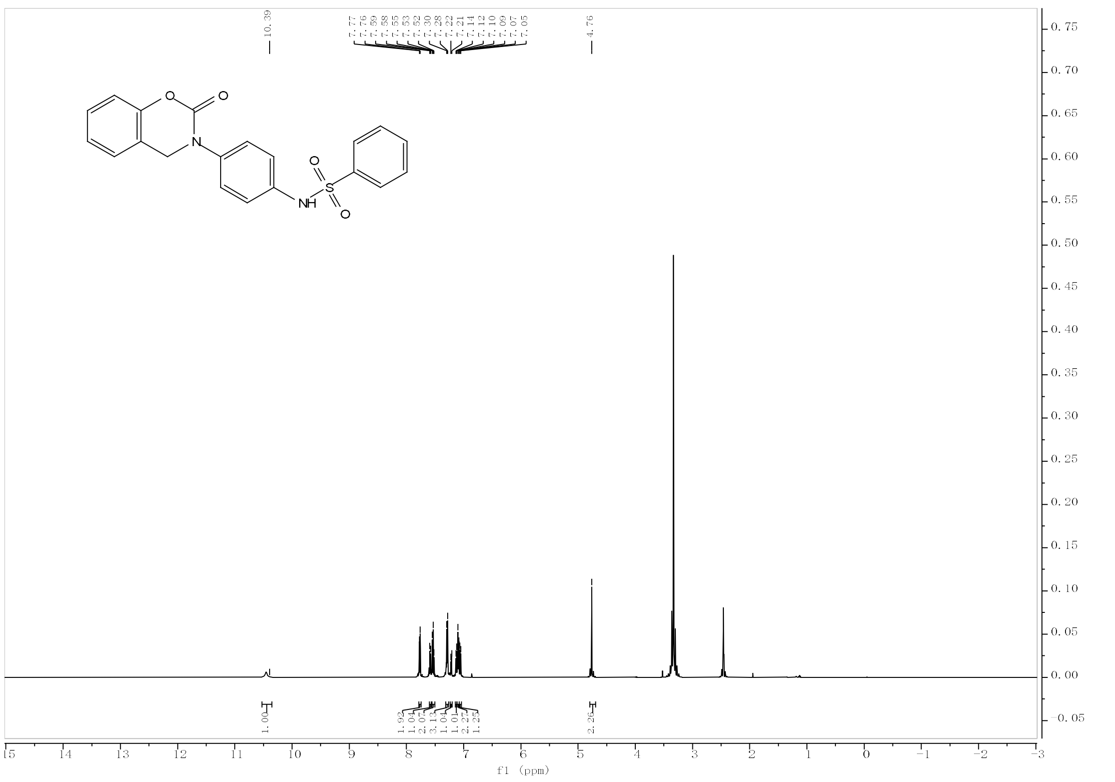

Figure 95. ^{1}H NMR (600 MHz, DMSO- d_6) spectrum of compound L31.

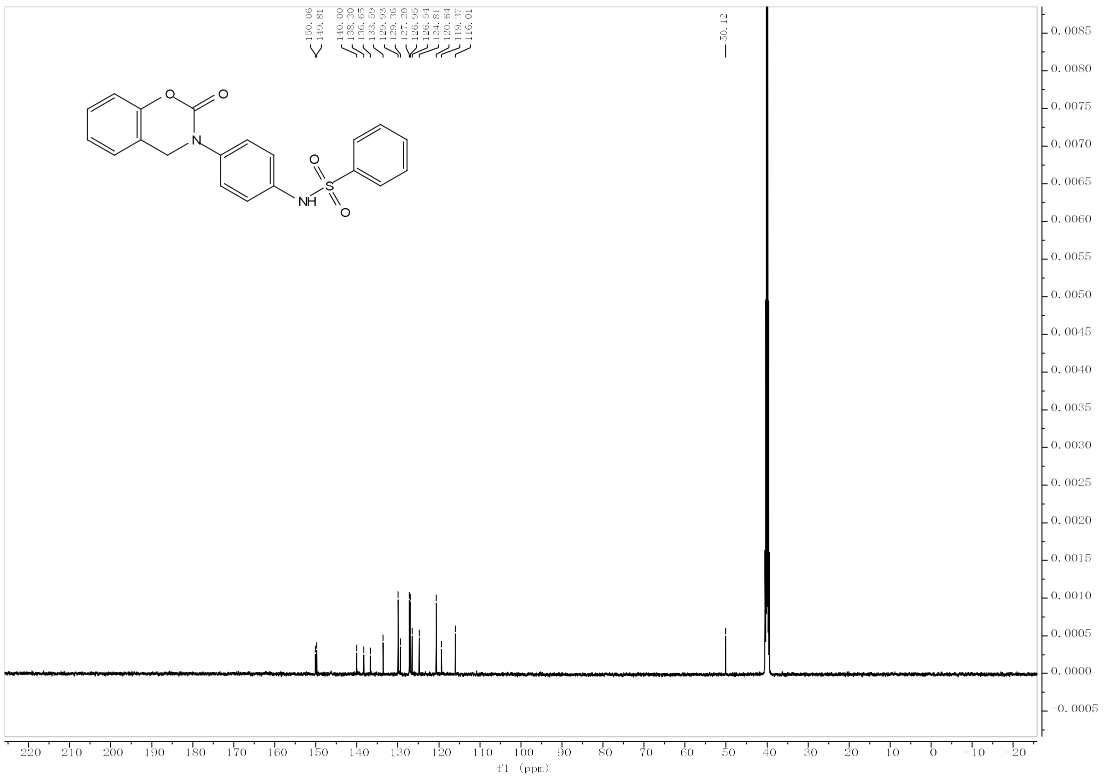

Figure 96 ^{13}C NMR (126 MHz, DMSO- d_6) spectrum of compound L31.

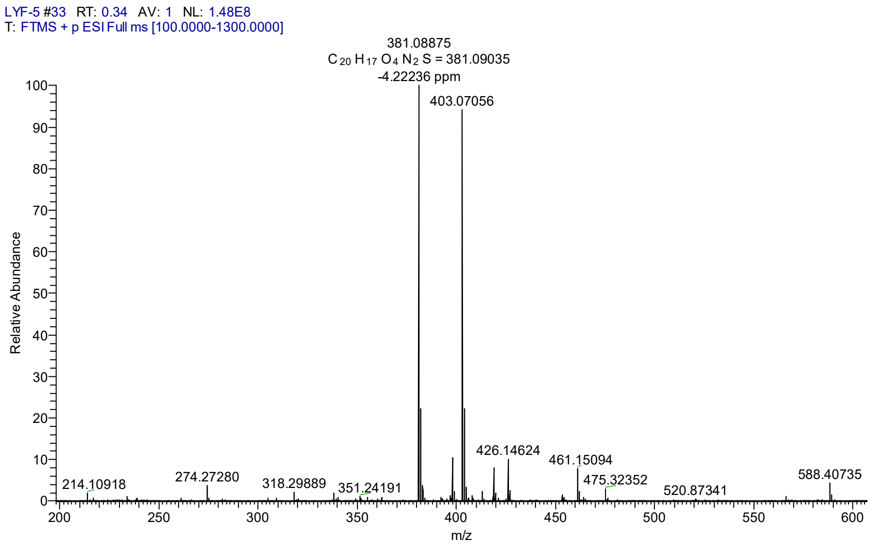

Figure 97. HRMS of compound L31.

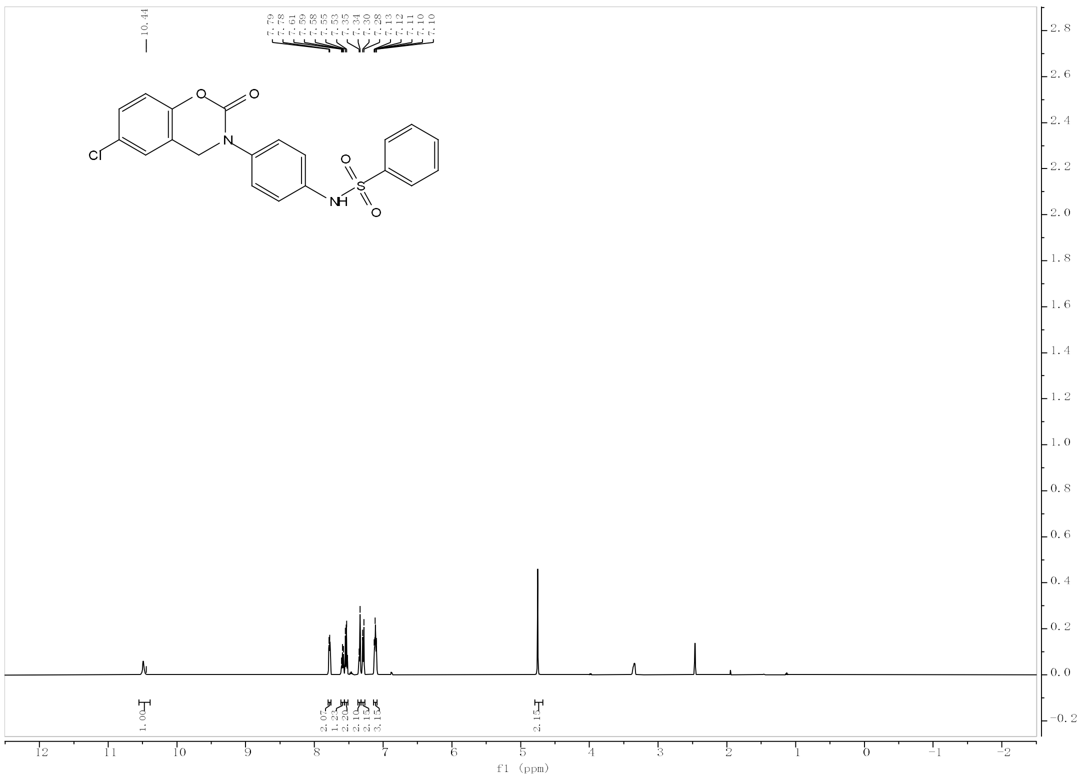

Figure 98 ^{1}H NMR (500 MHz, DMSO- d_6) spectrum of compound L32.

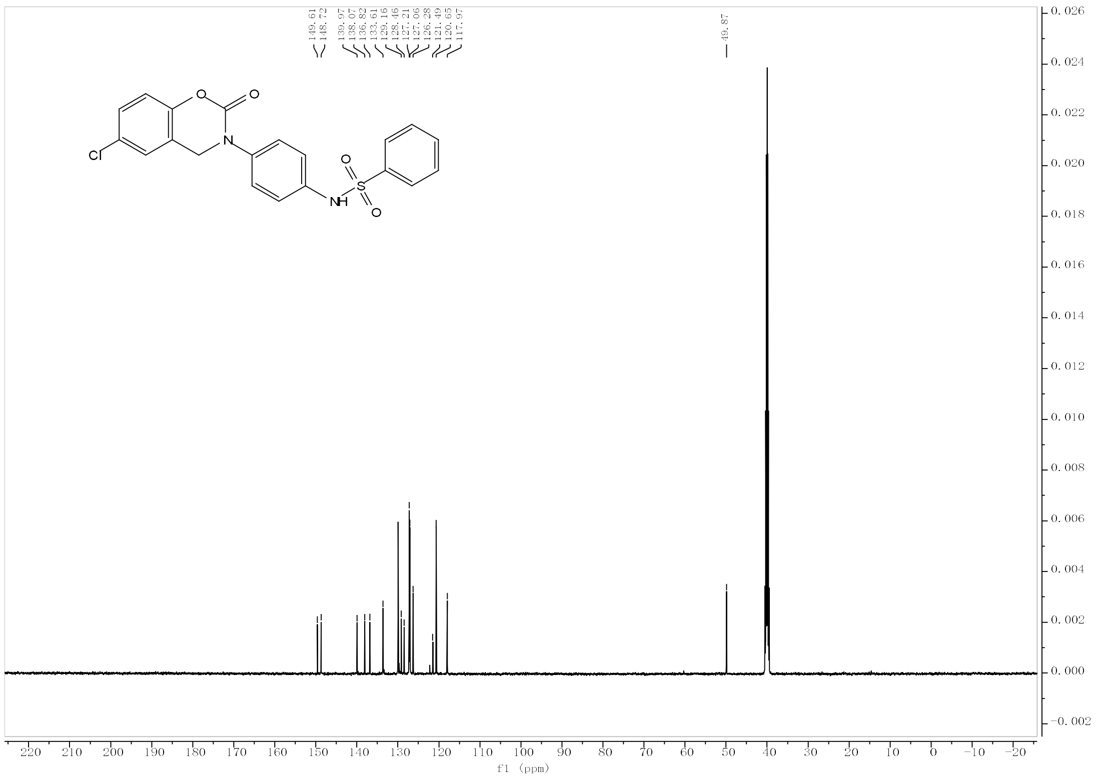

Figure 99 ^{13}C NMR (126 MHz, DMSO- d_6) spectrum of compound L32.

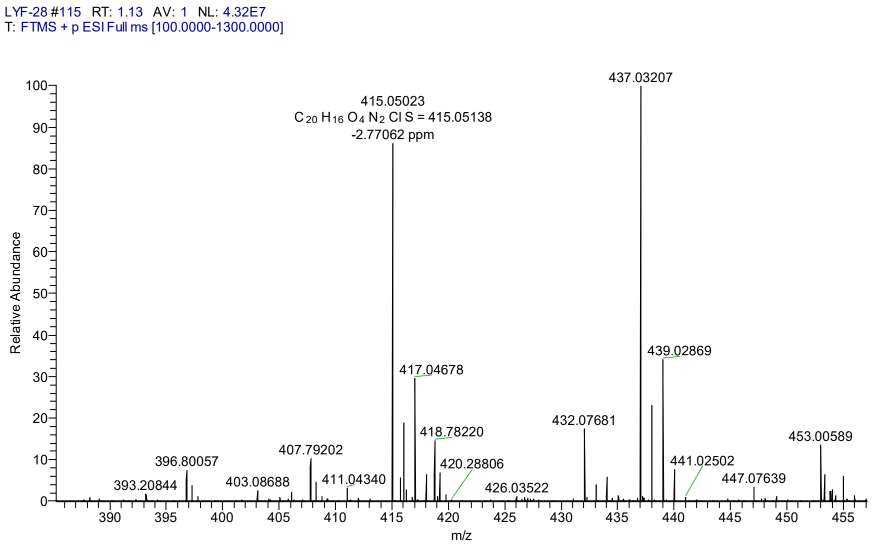

Figure 100. HRMS of compound L32.

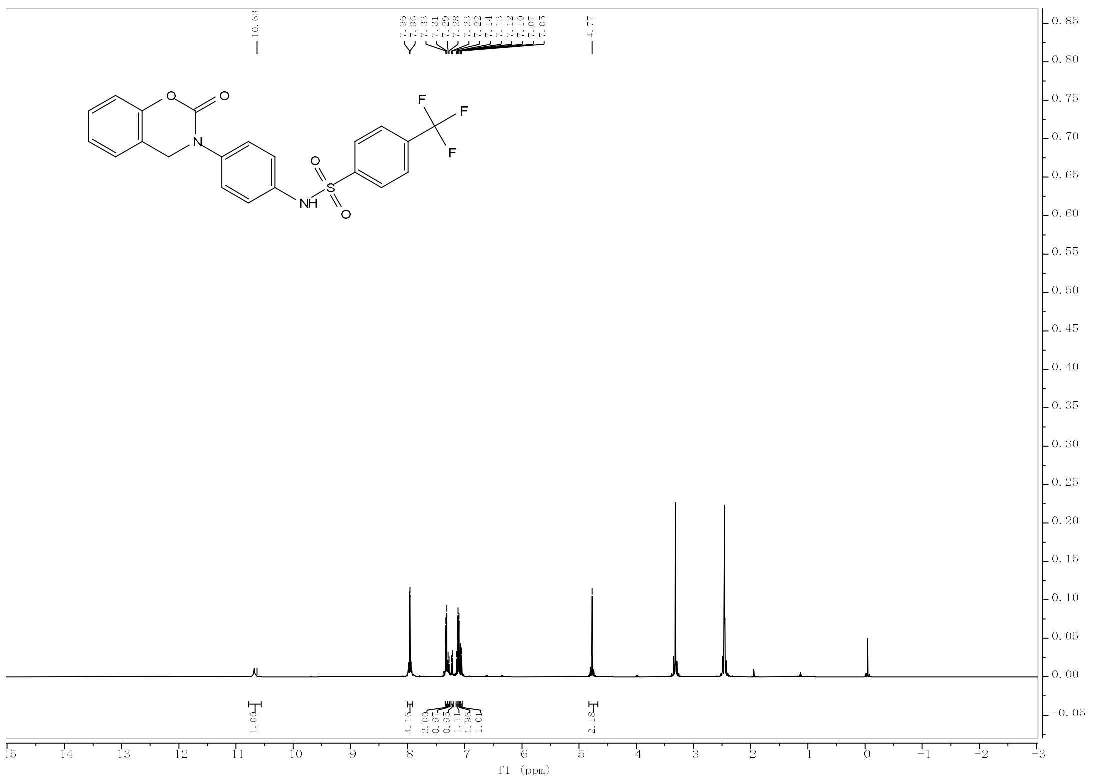

Figure 101. ^{1}H NMR (600 MHz, DMSO- d_6) spectrum of compound L33.

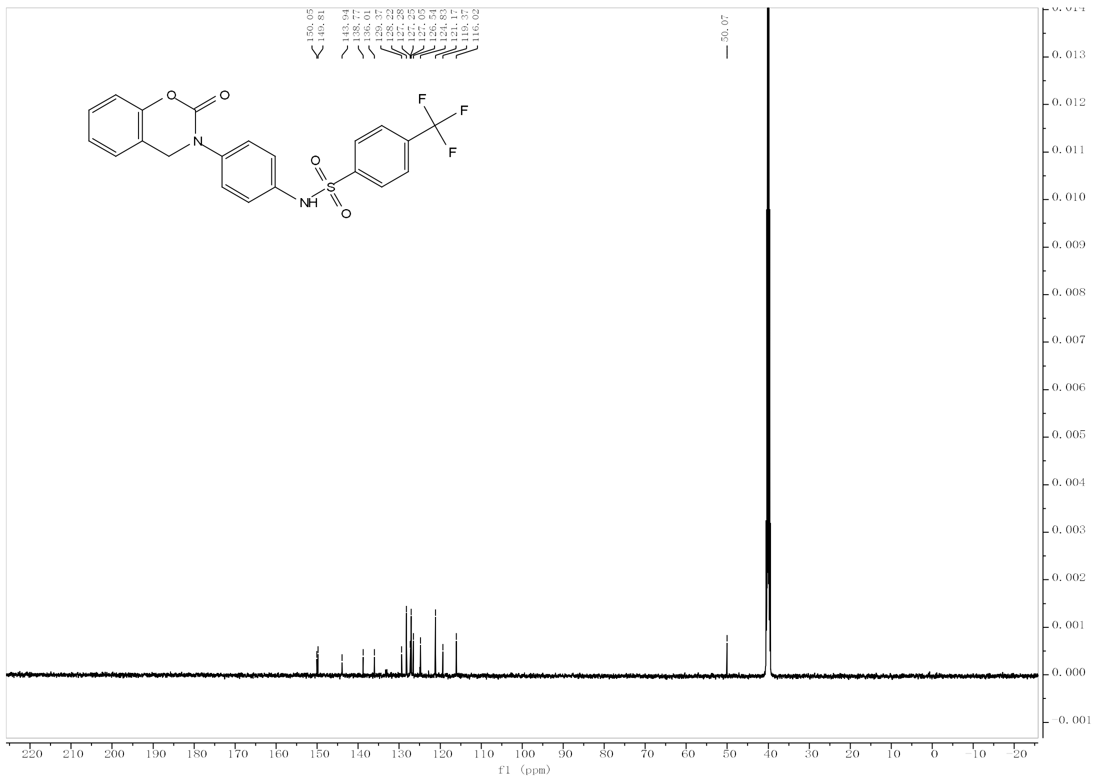

Figure 102 ^{13}C NMR (151 MHz, DMSO-d_6) spectrum of compound L33.

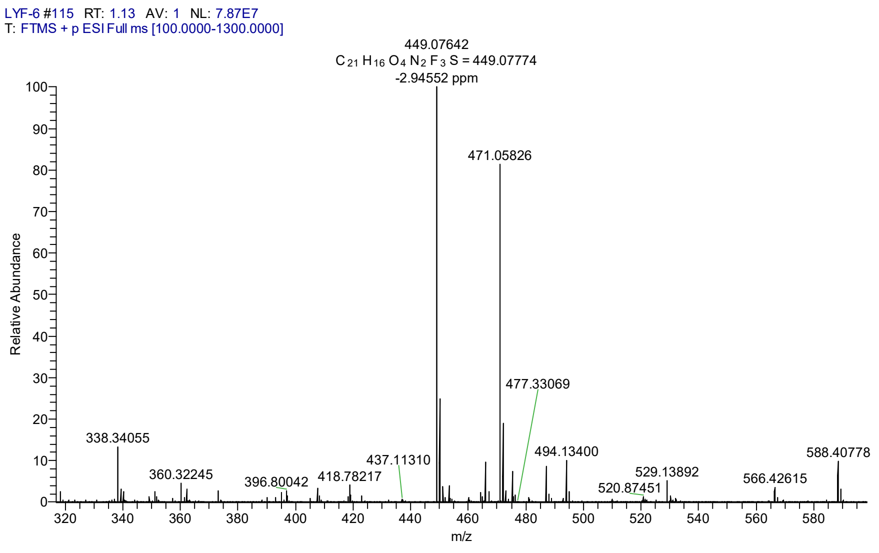

Figure 103. HRMS of compound L33.

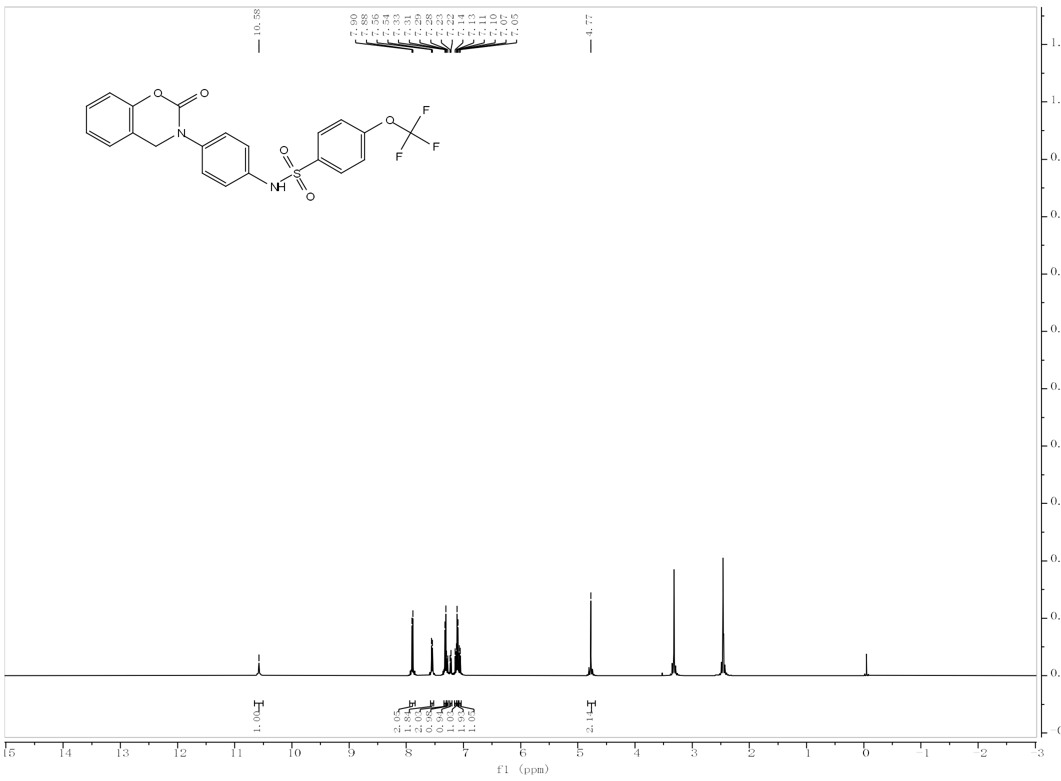

Figure 104 ^{1}H NMR (500 MHz, DMSO- d_6) spectrum of compound L34.

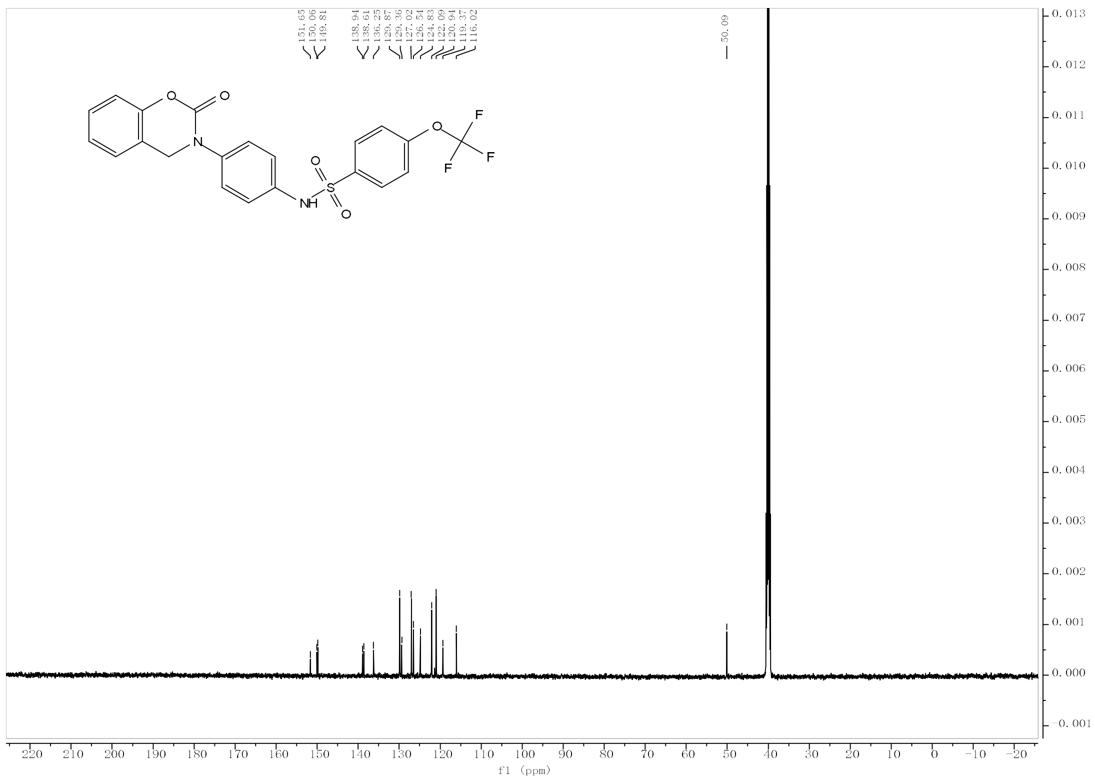

Figure 105 ^{13}C NMR (126 MHz, DMSO- d_6) spectrum of compound L34.

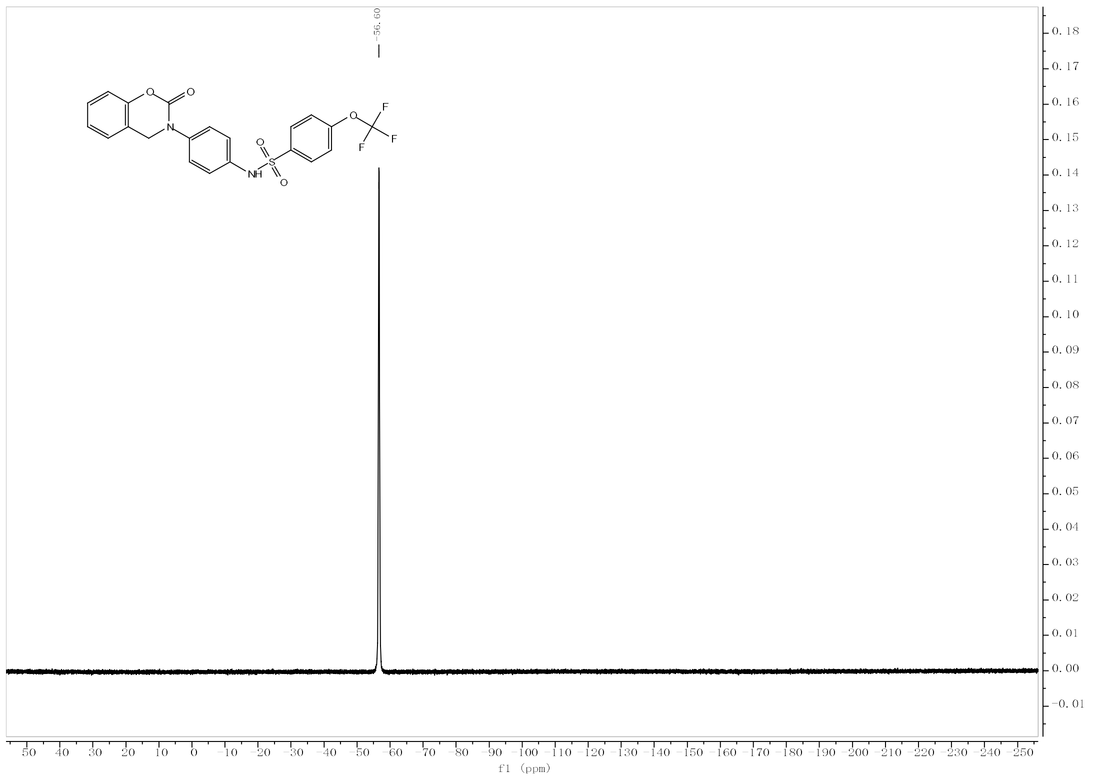

Figure 106 ^{19}F NMR (471 MHz, DMSO-d_6) spectrum of compound L34.

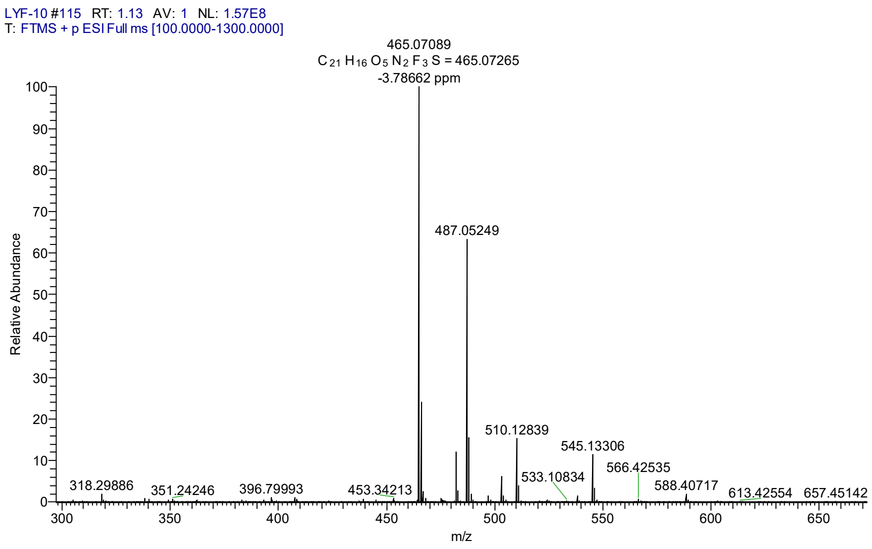

Figure 107. HRMS of compound L34.
